# AutonoMouse: High throughput operant conditioning reveals progressive impairment with graded olfactory bulb lesions

**DOI:** 10.1371/journal.pone.0211571

**Published:** 2019-03-06

**Authors:** Andrew Erskine, Thorsten Bus, Jan T. Herb, Andreas T. Schaefer

**Affiliations:** 1 The Francis Crick Institute, Neurophysiology of Behaviour Laboratory, London, United Kingdom; 2 Department of Neuroscience, Physiology & Pharmacology, University College London, London, United Kingdom; 3 Behavioural Neurophysiology, Max-Planck-Institute for Medical Research, Heidelberg, Germany; 4 Department of Anatomy and Cell Biology, Faculty of Medicine, University of Heidelberg, Heidelberg, Germany; Monell Chemical Senses Center, UNITED STATES

## Abstract

Operant conditioning is a crucial tool in neuroscience research for probing brain function. While molecular, anatomical and even physiological techniques have seen radical increases in throughput, efficiency, and reproducibility in recent years, behavioural tools have somewhat lagged behind. Here we present a fully automated, high-throughput system for self-initiated conditioning of up to 25 group-housed, radio-frequency identification (RFID) tagged mice over periods of several months and >10^6^ trials. We validate this “AutonoMouse” system in a series of olfactory behavioural tasks and show that acquired data is comparable to previous semi-manual approaches. Furthermore, we use AutonoMouse to systematically probe the impact of graded olfactory bulb lesions on olfactory behaviour, demonstrating that while odour discrimination in general is robust to even most extensive disruptions, small olfactory bulb lesions already impair odour detection. Discrimination learning of similar mixtures as well as learning speed are in turn reliably impacted by medium lesion sizes. The modular nature and open-source design of AutonoMouse should allow for similar robust and systematic assessments across neuroscience research areas.

## Introduction

The ultimate function of the brain is to orchestrate an organism’s behaviour appropriately according to its current environment. Behavioural techniques are therefore an important tool in neuroscience research [1–12]. Over the last decades, a number of technical advances have allowed for revolutionary improvements in efficiency and throughput in molecular biology [13], physiology [14], anatomy [15,16] and corresponding analysis techniques [14,17–20]. By contrast, with some notable exceptions [21,22] in particular in the analysis of movement patterns [12,23–28], behavioural techniques have not seen similarly radical advances in levels of standardisation and throughput, despite their importance to the field.

One core technique of behavioural analysis that has been used with great success to probe behavioural questions, operant conditioning [29], has seen advances in automation, but these approaches often still require an experimenter to be present throughout large parts of the experiment [27,30–33] and/or have limitations on the number of animals that can be trained simultaneously [12,34–37]. Furthermore, sessions of training often require frequent animal handling which can modulate stress in experimental subjects [38,39] and introduce additional sources of variability. Strikingly, it has been shown that the mere presence of an experimenter even without manual handling of the animals can affect experimental outcomes [40]. Animals may also need to be water restricted to motivate them to perform behavioural tasks which might lead to altered physiological state. This also requires a manual ongoing schedule of controlled water delivery and thus frequently is performed on animals housed individually (with social deprivation as a potential confound). To therefore improve the efficiency and throughput of this already powerful technique, we here describe the development of a fully automated operant conditioning system–AutonoMouse—for socially housed mice that allows simultaneous training and testing of large cohorts of mice continuously over periods of several months without water restriction.

To demonstrate the utility of AutonoMouse, we address a long-standing discussion of the function and mechanism of the early mammalian olfactory system. Results of lesioning experiments [41–43] in the mouse olfactory bulb and from knockout mice with OSN axon guidance defects [44] have been interpreted as evidence that relatively large disruptions to the olfactory bulb have little effect on olfactory function [45–48]. By contrast, other studies have contributed to opposing interpretations [49], for example that major disruptions cause deficiencies in odour recognition and discrimination, whilst even minor disruptions can affect recognition [50]. One explanation for these apparently divergent lines of evidence is that the parameter space of both olfactory system disruption and olfactory behaviour are not sufficiently explored.

Thus, we apply AutonoMouse to systematically analyse performance in a range of olfactory tasks before and after lesions of the olfactory bulb of varying size. Furthermore, we provide components lists, layouts, construction drawings, and step-by-step instructions for its construction as well as software and manuals in the appendix (S1 Appendix) to facilitate setup in other labs.

## Results

### AutonoMouse design

**Fig 1:**
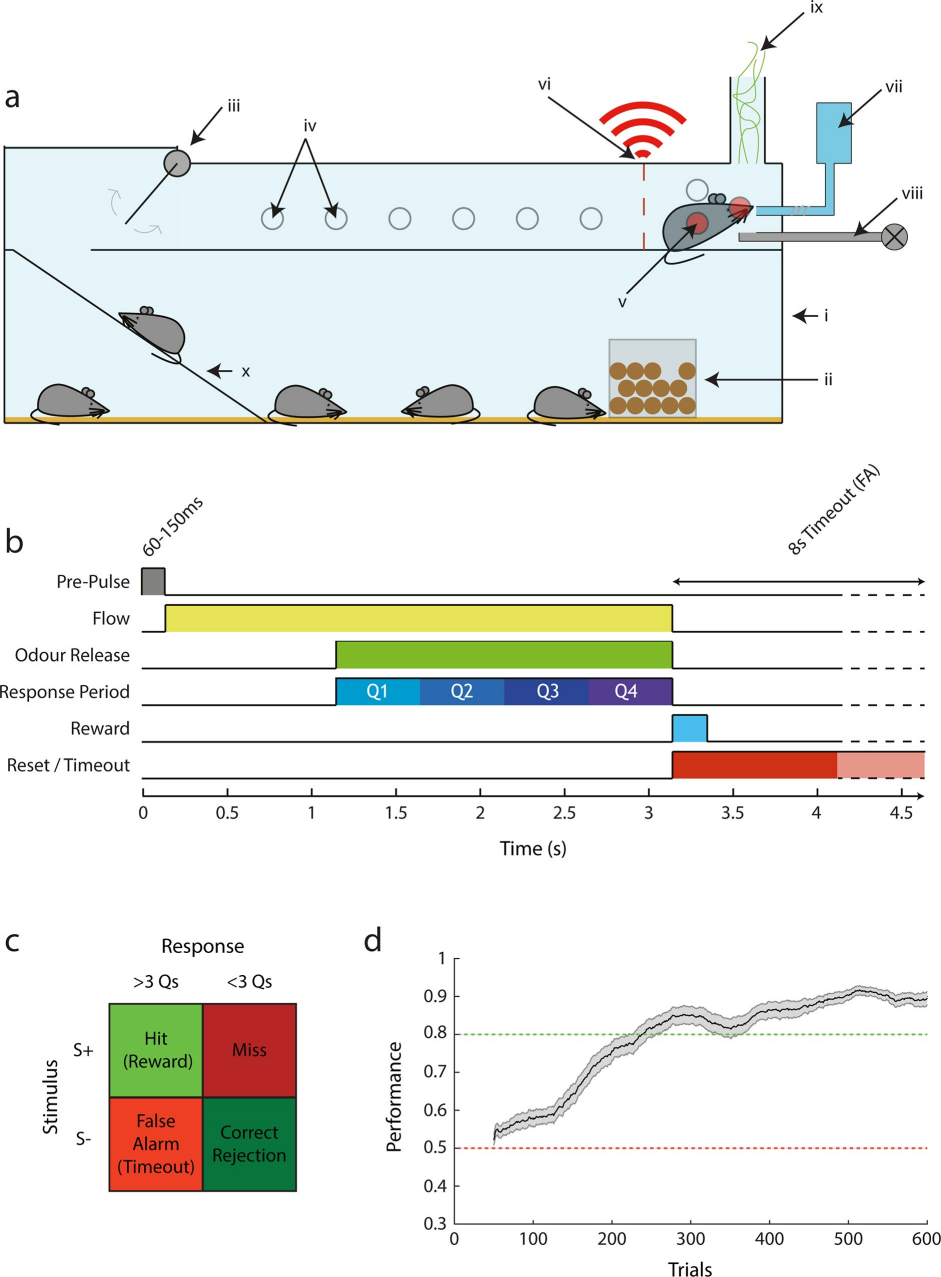
AutonoMouse schematic. (a) Basic design of the AutonoMouse system showing the link between the common home cage and the upper behavioural staging area. (i) Main home cage. (ii) Food hopper. (iii) Access door controlled by IR beam detectors. (iv) IR beam detectors, inactive as not blocked by animal. (v) Active IR beam detectors blocked by animal. (vi) Unique RFID readout. (vii) Water reservoir, pump and lickometer. (viii) Odour stimulus production. (ix) Odour exhaust. (x) Access ramp. (b) Time course of a typical olfactory go/no-go stimulus in the system. (c) Response/reward table showing trial outcomes depending on stimulus type and whether animal licks in ≥3 (+ve response) response period quarters or <3 (-ve response) (Q1-Q4 in (b). (d) Performance over trials in the first introduced olfactory discrimination task (n = 29, mean +/- sem; sliding average with 100 trial window).

AutonoMouse (Fig 1A, Figs A-E in S1 Appendix) houses cohorts of experimental mice within a common home cage. Cohorts of up to 25 mice can be simultaneously housed and trained within the system successfully (S1 Fig). Each mouse is individually tagged with a radio-frequency identification (RFID) chip such that individual performance can be monitored [51–54]. The home cage (Fig 1A, Figs A, B, F, G in S1 Appendix), contains various forms of environmental enrichment including bedding, chew-blocks, shelters and running wheels. The home cage also contains *ad libitum* access to solid diet. An upper chamber contains the behavioural staging area where water can be accessed (Fig 1A, Figs G and H in S1 Appendix). On entry to this area, an infra-red beam detector linked to a door-close mechanism is triggered (Fig G in S1 Appendix), isolating the animal within the chamber and preventing other animals from interfering with ongoing behaviour. The amount of power used to close the door could be adjusted on the door control module (see Methods: Door module) such that the force of door closing was small enough so as not to affect any animals that might enter quickly behind another animal. The access door itself was designed to leave a small gap (~0.5cm) between the bottom of the door and the floor to avoid trapping of tail/feet on entry to the staging area. In the staging area, the animal can automatically initiate a behavioural trial by blocking an IR sensor which triggers the control software to decode the animal’s RFID tag via the RFID coil also present in the chamber (Fig H in S1 Appendix). Detailed photographs of the staging area are shown in Figs B, C, D and E in S1 Appendix and a CAD drawing is given in Fig H in S1 Appendix. The software reads out the animal identity and can deliver appropriate sequences of behavioural trials specific to the behaving animal. Trials can be initiated at any time on entry to the staging area. These trials are assigned a particular valence (rewarded / unrewarded; Fig 1B and 1C) where successful completion of rewarded trials will result in the delivery of a small water reward, such that animals can gain their daily water intake by performing a set of trials per day. It is important to note, that all aspects of the system were designed with the goal of operation with minimal oversight for extended periods of time. This meant that, for example, the water reward delivery system was designed from a micro-pump that allowed precision delivery of small water doses (minimum 0.25μl) with CV 1% accuracy from an arbitrarily large reservoir with delivered volumes independent of usage (see methods). The housing chamber was designed to allow for bedding exchange without having to remove animals, again minimizing human interference (see Figs F, J in S1 Appendix).

In summary, this design means that AutonoMouse can socially house a large experimental cohort, provide daily living requirements, and train them simultaneously in a high-throughput manner to a high performance level (Fig 1D, throughout this work we define performance as a weighted sum of correct responses and rejections in response to conditioned vs. unconditioned stimuli respectively, see Methods: Analysis and Statistics). The approach to house a large group of animals socially with a single conditioning chamber further allows the conditioning chamber itself including stimulus delivery to be designed without compromising on quality, yet being cost-effective (as only one system is needed for up to 25 animals). As a result of the complete automation of the system, minimal experimenter presence or intervention is required for training. Furthermore, group-housing in a social environment from shortly after weaning (see methods) allowed us to use all-male cohorts (as well as all-female ones) without any notable display of aggressive behaviour [55,56]. In general, this design is expected to have a significant effect on the stress levels of animals housed in the system, and therefore improve the reliability of behavioural results [57]. Water dispense rewards in the conditioning chamber could be made conditional on the animals’ behaviour and task structure, according to the profile of sensors installed in the chamber (e.g. go/no-go, 2-alternative forced choice, motor pattern [58]). Though AutonoMouse could potentially be applied to operant tasks in any sensory modality, we here focus on olfactory go/no-go tasks with lick rate as the response measure to demonstrate its utility (Fig 1B and 1C, note the use of auditory tasks as control in Fig 6).

### Consistency and reliability of training in AutonoMouse

**Fig 2:**
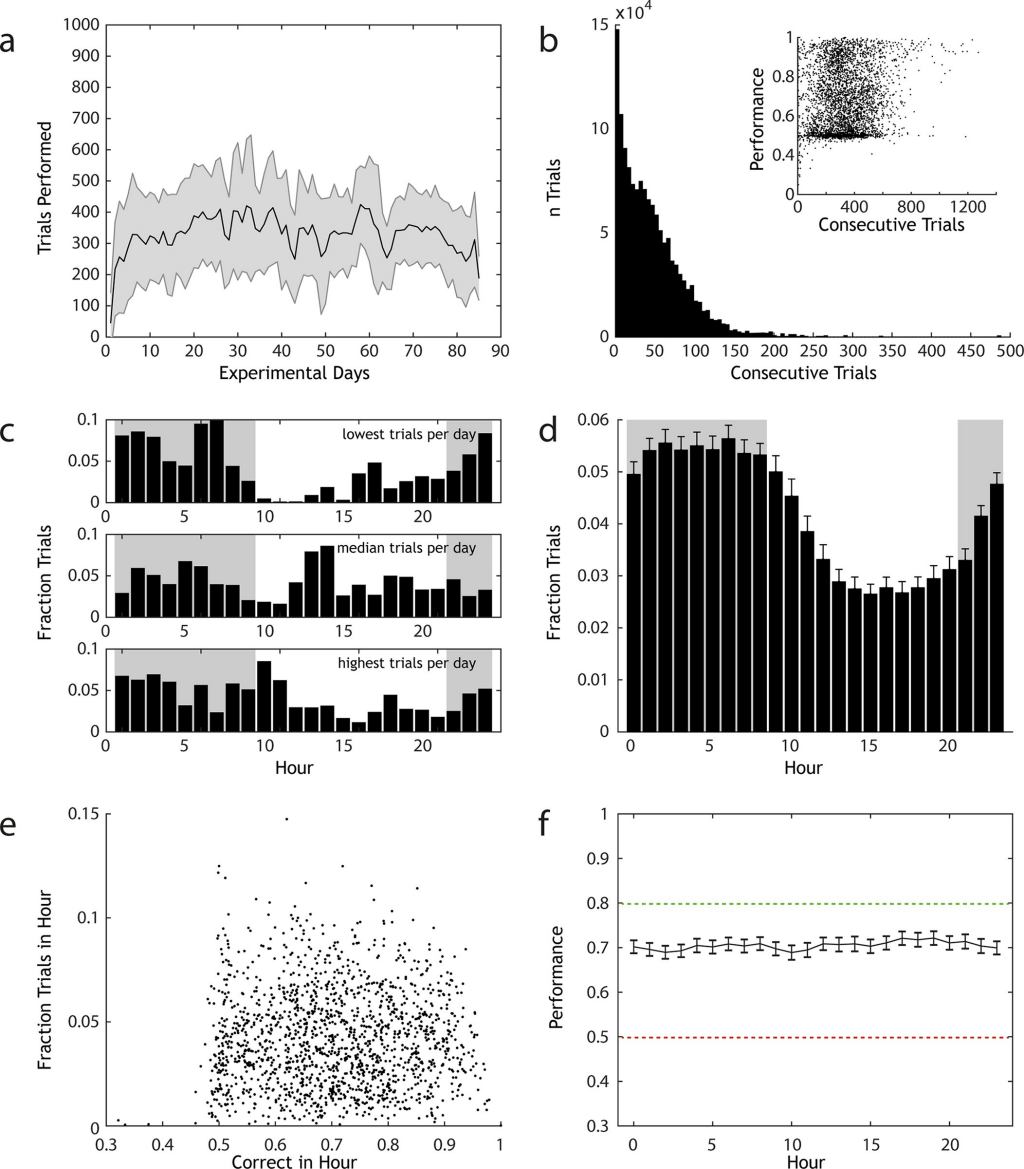
AutonoMouse gives high volumes of reliable behavioural data. (a) Number of trials performed per day by animals housed in AutonoMouse (n = 67, mean +/- std, total trials = 1,351,320). (b) Number of trials that are performed in sessions of continuous trial sequences (<20s between trials, mean session length = 38). Inset: performance in each set of consecutive trials plotted against the number of trials within the set. (c) Fraction of trials performed in each hour of the day for 3 representative animals that performed the least, median and most trials per day. (d) Mean fraction trials performed each hour for all animals (mean +/- sem). (e) For each animal, the overall fraction of trials performed in each hour vs. the average accuracy in that hour. There is no appreciable correlation between these variables (R = 0.006, p > 0.05). (f) Average performance accuracy in each hour of the day, averaged over all animals (mean +/- sem).

In olfactory go/no-go discrimination tasks, animals performed between 150 and 560 trials per 24 hours (mean 333 trials per day +/- 166, n = 67 animals, 1,351,320 total trials), with 50% of these trials performed in continuous stretches of 38–490 trials (Fig 2A and 2B). The number of trials performed in a continuous stretch was weakly but significantly correlated to performance accuracy (Fig 2B, inset, Pearson correlation coefficient R = 0.15, p = 6.5x10^-21^). This can be interpreted in a number of ways. One interpretation is that animals that are generally accurate in the behavioural task tend to perform more trials than animals that have not sufficiently learned the task. Another interpretation is that performance tends to increase over continuous stretches of trials, and increases sufficiently that long stretches of trials will inevitably have higher mean performance scores, regardless of the initial behavioural ability of the animal.

If the former interpretation were correct, one would expect to observe a positive correlation between animals’ mean overall performance and their mean daily number of trials; as well as the absence of a correlation between mean performance and the mean length of consecutive trials performed. In the case of the latter interpretation, one might still observe a positive correlation between mean performance and mean daily trials but this would be accompanied by a strong positive correlation between mean performance and mean length of consecutive trials performed; as if performance always increases over continuous stretches of trials then one would expect animals that tend to perform longer stretches to have generally higher performance. We found that animals with higher average performance across all experiments tended to perform more trials per day (S2A Fig), but there was no statistically significant relationship between average performance and mean of consecutive trial lengths for each animal. Therefore, the observation that performance is generally higher in longer stretches of consecutive trials (Fig 2B, inset) is best interpreted as evidence that animals that are more accurate in the behavioural task tend to perform more trials than other animals.

Mice are crepuscular animals and their activity patterns while housed in AutonoMouse closely followed the internal day-night cycle of the system (Fig 2C and 2D). Activity reached its minimum during the 7^th^ hour of the light phase and peaked 15 hours later in the 10^th^ hour of the dark phase. Total activity was significantly higher during the dark phase when compared to activity in the light phase (night: 21:00–09:00, day: 09:00–21:00. Fraction trials night: 0.61 +/- 0.12, fraction trials day: 0.39 +/- 0.12, t-test t = 16.658, p = 1.35x10^-57^, df = 1606). Although activity patterns of AutonoMouse housed animals changed during the course of the day, accuracy in the performed task did not. Average accuracy within a particular hour of the day was uncorrelated to the fraction of total trials performed in that hour (Fig 2E, Pearson correlation coefficient R = 0.006, p = 0.81), and average performance across all mice binned by hour showed no significant difference between hours (Fig 2F, 1-way ANOVA, F = 0.34, p > 0.99).

### Odour delivery

**Fig 3:**
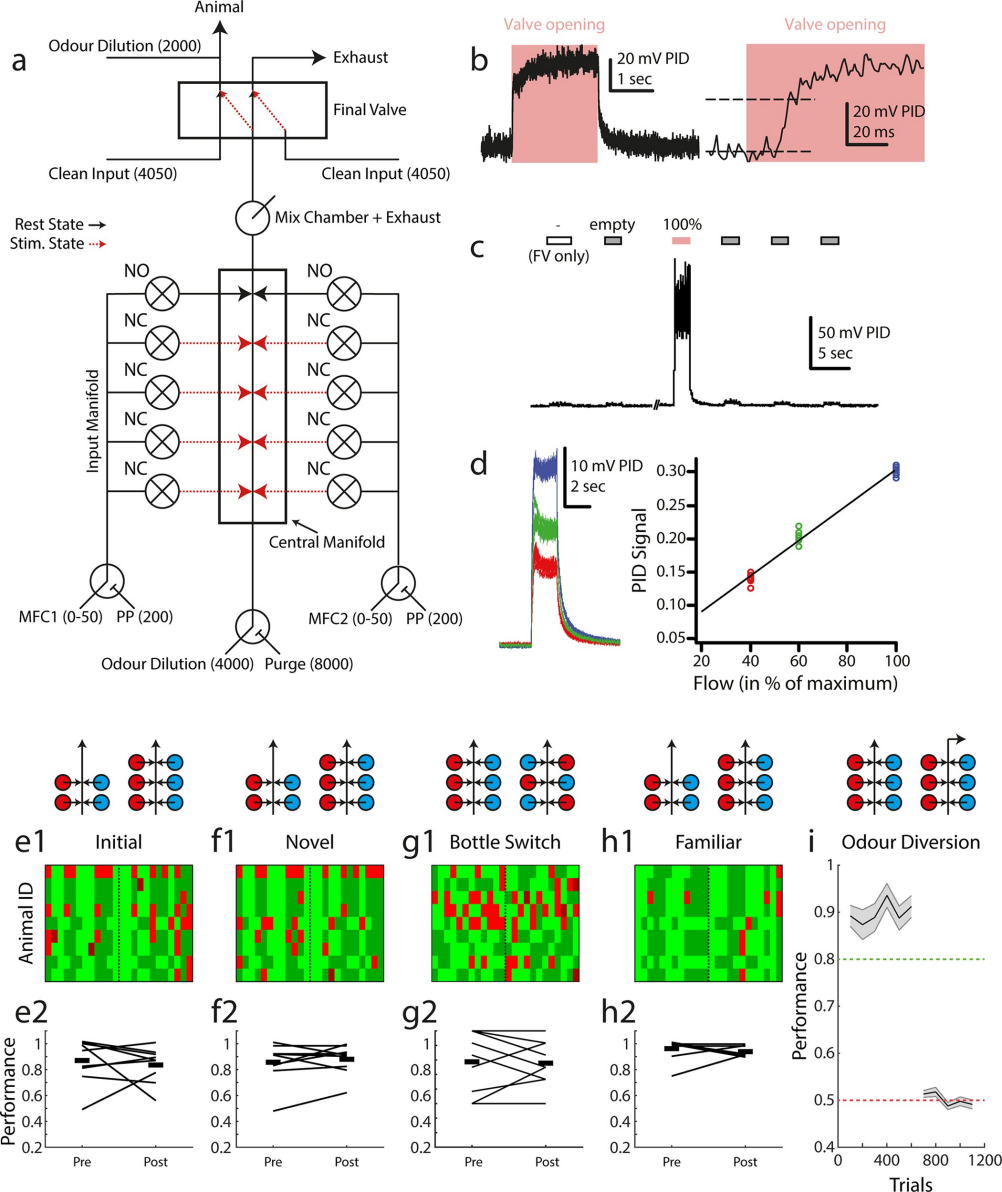
Odour delivery. (a) Olfactometer schematic. Numerical values shown indicate supplied air flow in cubic centimeters per minute. Black / red lines indicate resting state (between trials) and odour delivery state air pathways respectively. (b) Example PID recorded odour trace. Left: entire recorded pulse, right: at higher temporal resolution. (c) Example recording from the olfactometer switching between final valve only (FV only), empty (non-odourised) input and odourised input (100%, red). (d) Output odour concentration is reliably controlled by airflow dilution. Left: 10 overlaid odour pulses during maximum MFC input (blue), 60% MFC input (green) and 40% MFC input (red). Right: summary of PID recorded odour signal in the three conditions. (e1) Map of trial performance before and after introduction of an extra valve set (as schematised above: red = odour 1, blue = odour 2) into the odour stimulus production, during the first odour pair discrimination learnt by this set of animals (n = 9). Each row corresponds to an animal, with each column in the row corresponding to a trial (pre-switch n = 12, post-switch n = 12). The vertical dashed line indicates the point at which new valves were introduced. Light green: hit, dark green: correct rejection, light red: false alarm, dark red: miss. (e2) Summary of data shown in (e1) showing mean performance before and after for each animal in the group (connecting black line, start and end values jittered for ease of visualisation). Thick black lines indicate the mean of the group pre- and post- new valve introduction. (f1), (f2), (g1), (g2), (h1), (h2) Same analysis as in (e1), (e2) but for novel and familiar odour pair discrimination respectively. (g1), (g2) shows performance on the novel odour pair task before and after a full bottle switch with randomised placement. (i) Performance in a standard odour pair discrimination (EB vs. AA) followed by diversion of the odour stream in the olfactometer final valve (mean +/- sem). Performance analyses in 100 trial bins for each animal.

In order to run AutonoMouse on olfactory conditioning for long-term experiments with minimal human interference, we required a highly stable olfactometer with minimal inter-channel contamination and reliable signal output. We therefore designed an all-air-dilution olfactometer which used pure, undiluted chemicals in individual glass vials for multiple separate odourised channels with consecutive stages of airflow dilution for concentration control (Fig 3A). Square-pulse stimuli could be reliably generated with rapid rise time (Fig 3B, rise from baseline to 90% of maximum in 20ms). Contamination between odour channels was minimal and only release of odourised channels produced any appreciable odour signal (Fig 3C). Signal amplitude was reliably controlled by the air-dilution method and input flow rate was linearly related to odour output level (Fig 3D).

It is crucial that any stimulus delivery device provides salient behavioural cues for the stimulus of interest only. Any extraneous variables must not be informative of the reward condition of the stimulus. To achieve this, in particular during initial training we trained animals on (pure) odours delivered through combinations of valves (mixing e.g. 20 ml/min odour A from valve 1 with 80 ml odour A from valve 2 and changing those ratios and valves from trial by trial). This was to assure that whilst valve clicking, possible flow idiosyncracies and potential contaminations varied from trial to trial, the intended cue– 100 ml/min odour A–remained constant. We confirmed that odour was indeed the only salient cue in our olfactometer by training animals on a subset of available odour channels, then introducing new odour channels (never used before with this specific odour for the given animal) after above chance performance was reached (similar as we had done previously in semi-manual settings [59–61]). If animals were learning cues other than odour identity (e.g. valve noise, flow differences, contamination etc.) then performance accuracy would significantly drop on introduction of new channels. Performance, however, was indistinguishable before and after introduction of new channels (Fig 3E, 3F and 3H; paired t-test pre vs. post performance, initial: mean ± sd = 0.83 ± 0.16 vs. 0.77 ± 0.22, t = 0.6568, p = 0.52, df = 16; novel: 0.85 ± 0.14 vs. 0.89 ± 0.16, t = -0.5505, p = 0.59, df = 16; familiar: 0.97 ± 0.05 vs. 0.92 ± 0.09, t = 1.5947, p = 0.13, df = 16), showing that the intended odour stimulus information was the only cue being learnt. We also tested whether input-level contaminants of the olfactometer could contribute to learning by performing a control in which odour bottles were physically switched in the olfactometer (Fig 3G). If animals were able to learn input-level contaminants as a salient behavioural cue then performance should drop after this bottle switch (since odour delivery is constant, but pattern of contaminants has changed), but we again observed no significant difference in performance (paired t-test pre vs. post performance, mean ± sd = 0.7870 ± 0.2129 vs. 0.7778 ± 0.1559, t = 0.1818, p = 0.8602, df = 8). Consequently, completely removing odour stimulus information by diverting odour release (final valve always diverting odour lines to exhaust port) reduced GNG performance to chance levels (Fig 3I, t-test final odour diversion performance vs. chance t = -0.9363, p = 0.38, df = 8).

### Quality of conditioning in AutonoMouse

**Fig 4:**
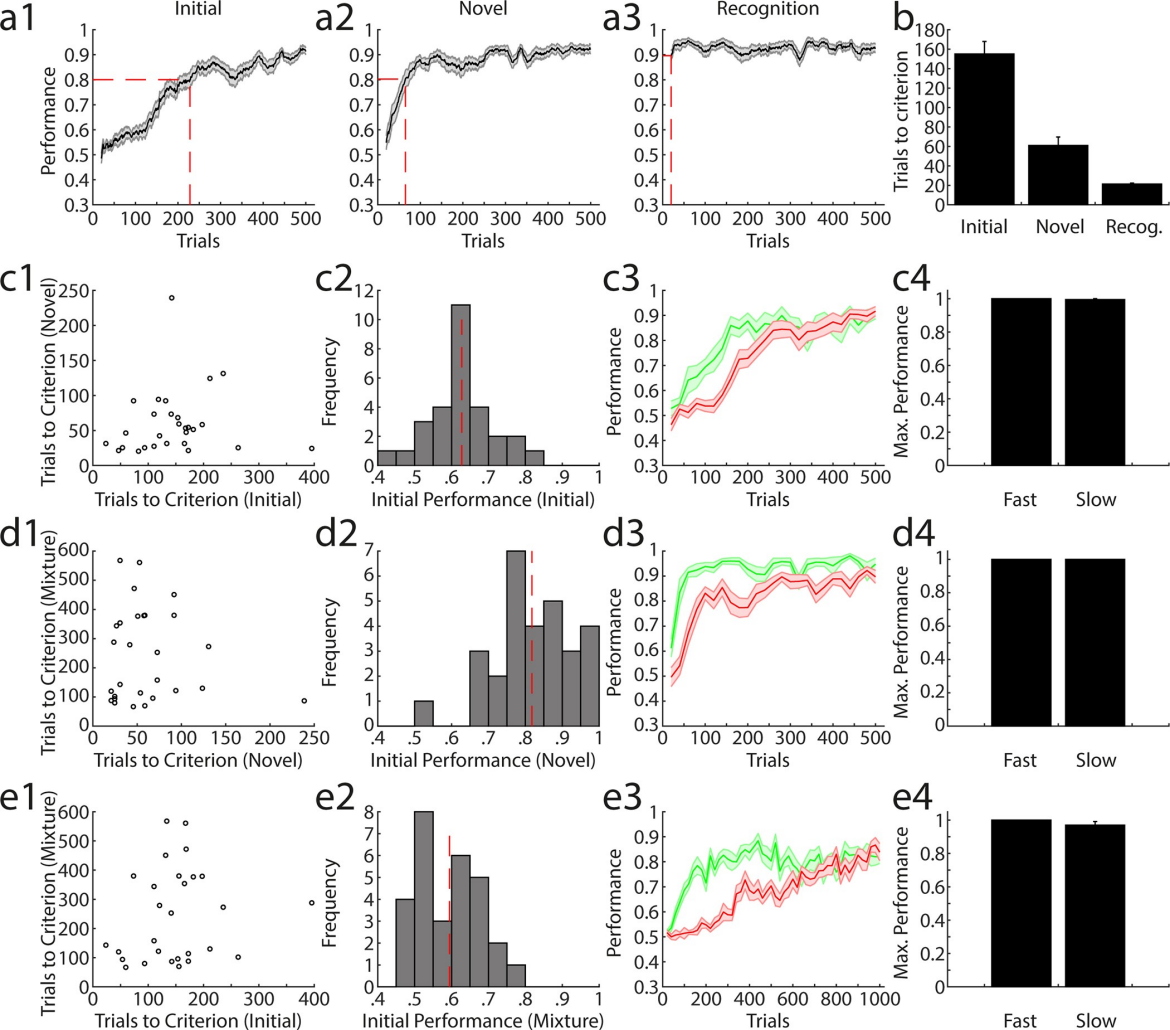
Quality of learning during olfactory discrimination in AutonoMouse. (a1) Average performance in the initial encountered odour pair discrimination (n = 29, mean +/- sem) calculated in a 20 trial moving average. (a2) Same as in (a1) for a novel odour pair discrimination (n = 29). (a3) Same as in (a1) for a previously learned odour pair. (b) Number of trials needed to reach criterion (0.8) over animals and tasks shown in (a1), (a2), (a3) (mean +/- sem). (c1) Trials needed to reach criterion (TTC) for a novel odour pair vs. TTC on the initial odour pair discrimination for all animals. (c2) Histogram of performance in the first 200 trials of the initial odour pair discrimination. Dashed red line indicates mean performance across animals. (c3) Average performance separated by whether accuracy level was greater than the mean performance (fast, green) or lower than the mean performance (slow, red) in the first 200 trials of the initial odour pair discrimination (mean +/- sem). (c4) Maximum performance levels reached for animal in the fast and slow groups (mean +/- sem). (d1) as in (c1) with trials to criterion in mixture discrimination vs. trials to criterion in novel odour pair discrimination. (d2) as in (c2) for novel odour pair discrimination. (d3) as in (c3) for novel odour pair discrimination. (d4) as in (c4) for novel odour pair discrimination. (e1) as in (c1) with trials to criterion in mixture discrimination vs. trials to criterion in initial odour pair discrimination. (e2) as in (c2) for mixture discrimination. (e3) as in (a3) for mixture discrimination. (e4) as in (c4) for mixture discrimination.

Similar to comparable conditioning experiments with a more manual component [30,32,62], mice rapidly learned to discriminate between two odours in the AutonoMouse system (Fig 4A1, 4A2 and 4A3). After 7 days of (automated) habituation and pre-training (see Methods for protocol), the first odour pair was learned in 1–2 days (performance >80% correct) or 54–398 trials (Fig 4A1 and 4AB; performance averaged over 20 trials, trials to criterion indicates the first trial point at which performance averaged over the preceding 20 trials was equal to or exceeded 80%). The second, subsequent odour pair was learned in approximately half the time / number of trials (20–246 trials; “20” implies >80% performance already within the first 20 trials) (Fig 4A2 and 4B). Recognition of the initially learned odour was virtually instantaneous (20–46 trials) (Fig 4A3 and 4B cf. [50]).

We asked whether there was an appreciable difference in the learning quality of different animals housed in the system, based on the observation that learning rates in the initial stages of various odour tasks were variable across animals (see Tables 1–3 for the detailed training schedules). We first analysed the number of trials needed for animals to reach a criterion level of discrimination performance to determine whether this was a constant feature for individual animals across different olfactory tasks. Over three tasks—initial odour pair learning (Fig 4C), novel odour pair learning (Fig 4D) and a binary mixture discrimination (Fig 4E)–there was no appreciable correlation in trials to criterion (Fig 4C1, 4D1 and 4E1), suggesting that although animals varied in their learning rates, they were not necessarily consistently poor or exemplary in their ability to reach criterion level over all tasks.

**Table 1:** Injection sites.

Coordinate ID	1	2	3	4	5	6	7	8	9	10
**X:**	0.85	0.85	0.85	0.85	0.85	0.85	0.85	0.85	0.65	0.65
**Y:**	0.85	0.85	0.85	1.15	1.15	1.15	1.45	1.45	0.85	0.85
**Z:**	0.7	1.1	1.4	0.7	1.1	1.4	0.7	1.1	1.1	1.4
**n Injections (2.3nl)**	66	33	33	66	33	33	66	33	66	33
**Solution (sham)**	PBS	PBS	PBS	PBS	PBS	PBS	PBS	PBS	PBS	PBS
**Solution (S)**	NMDA	PBS	PBS	PBS	PBS	PBS	PBS	PBS	PBS	PBS
**Solution (M)**	NMDA	PBS	PBS	PBS	PBS	PBS	NMDA	PBS	PBS	PBS
**Solution (L)**	PBS	PBS	NMDA	NMDA	NMDA	NMDA	NMDA	NMDA	PBS	PBS
**Solution (XL)**	NMDA	NMDA	NMDA	NMDA	NMDA	NMDA	NMDA	NMDA	PBS	PBS
**Solution (XXL)**	NMDA	NMDA	NMDA	NMDA	NMDA	NMDA	NMDA	NMDA	NMDA	NMDA

Table shows injection sites for each lesion group used in the experiment. For each coordinate (1–10) the x/y/z positions of injections are shown. X position refers to mm away from bregma in the rostro-caudal axis. Y position refers to mm away from bregma in the medio-lateral axis. Z position refers to depth from the surface of the brain. For each injection site, a number of 2.3nl injections were made, given by the n injections row. For each injection site, the solution injected is shown depending on the desired lesion extent (sham, S, M, L, XL, XXL). PBS was at 1% dilution. NMDA solution was 10mg/ml dissolved in 1% PBS.

After injection completion, the craniotomy was resealed using silicone elastomer (*KwikCast*, *World Precision Instruments*, *FL USA*) and the skin incision was sutured closed (*Silkam 7/0*, *Braun*, *Tuttlingen Germany*) and cleaned with 1% clorhexidine scrub. Animals were given meloxicam (Metacam; 2mg/kg) injected sub-cutaneously for post-operative analgesia. Mice were removed from the stereotaxic apparatus and placed in a warm recovery chamber (*Thermo Scientific*, *MA USA*) (36°C) until recovery from anaesthesia was observed (righting reflex regained). Following surgery, animals were singly housed for 3 days, and then returned to the AutonoMouse home cage.

**Table 2:** Training schedules (group 1).

Task (Group 1)	Odours / Hz	Task type
1	Cinn. vs. ACP	Initial
2	EB vs. AA	Novel
3	EB vs. AA	Mixture
4	Time delay (25 days)	N/A
5	EB vs. AA	Familiar
6	V vs. P	NTS
7	V vs. P	NTM
Lesion		
8	EB vs. AA	Familiar
9	CN vs. EU	Novel
10	V vs. P	NTS
11	V vs. P	NTM
12	CN vs. EU	Familiar
13	CN vs. EU	Mixture
14	EB vs. AA	Familiar

The sequence (numbered) of behavioural tasks for cohort 1 in the lesion study is shown (n = 6 female). The task type is shown for each, as well as the odour pair or auditory frequency used. ‘Lesion’ row indicates the point at which lesions were induced. Task 4 is a time delay–intended to investigate performance in a familiar odour task after a period of not performing odour discrimination.

**Table 3:** Training schedules (group 2).

Task (Group 2)	Odours / Hz	Task type
1	EB vs. AA	Initial
2	CN vs. EU	Novel
3	EB vs. AA	Familiar
4	EB vs. AA	Mixture
Lesion		
5	CN vs. EU	Familiar
6	EB vs. AA	Familiar
7	EB	S+ detection
8	EB	S- detection
9	0.3 vs. 3 kHz	Auditory
10	5 vs. 10 kHz	Auditory

As in Table 1 for cohort 2 (n = 14)

For each task we defined a group of ‘fast’ and ‘slow’ learners based on their performance within the first 200 trials of the task (Fig 4C2, 4D2 and 4E2), where slow animals were those performing at less than the mean performance in this task period. These groups were defined separately for each task given the above finding that rate of learning was not consistent across tasks. Although performance in the slow group was worse than the fast group in the initial stages of each task (by construction; Fig 4C3, 4D3 and 4E3), final discrimination performance was comparable between the groups; and the maximum discrimination accuracy was indistinguishable between fast and slow learners (Fig 4C4, 4D4 and 4E4). Thus, in the AutonoMouse system, virtually all animals can be trained to effectively perform odour discrimination tasks, even if they are initially poor performers.

### Training without water restriction

**Fig 5:**
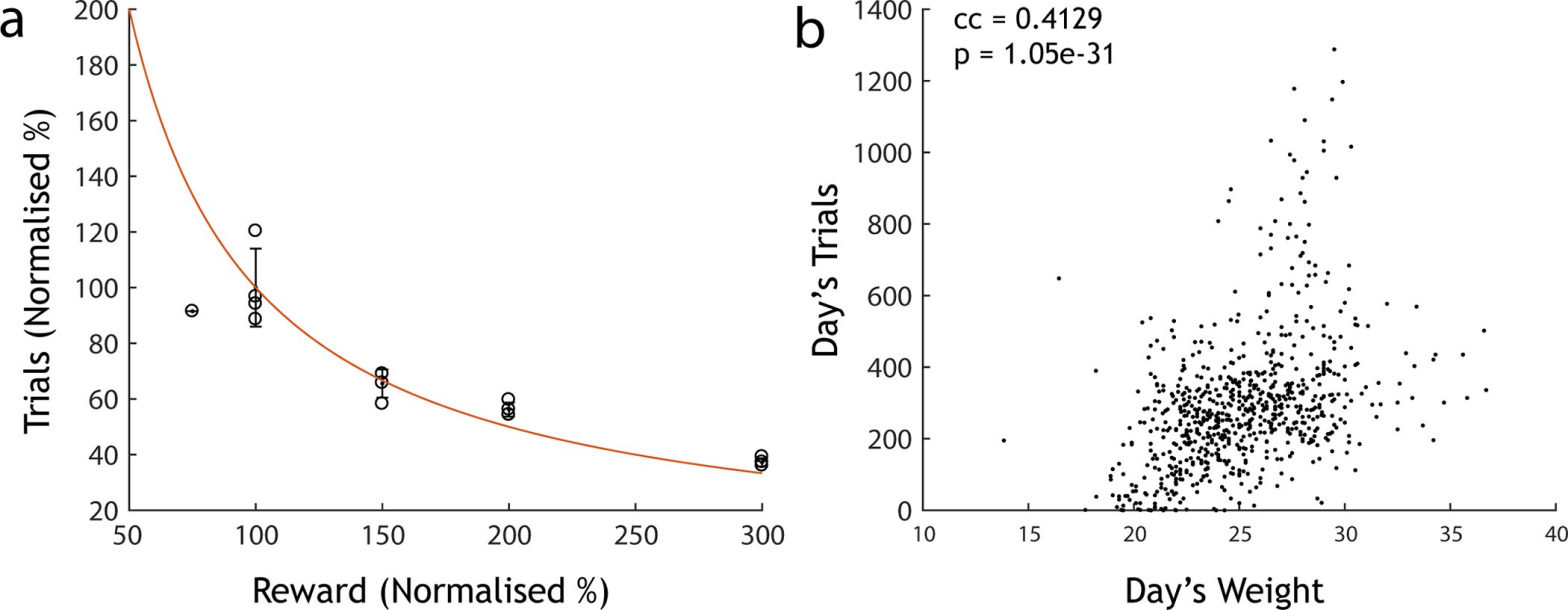
Mice are motivated but not water restricted. (a) Number of trials performed normalised to mean of trials performed across animals at 100% reward vs. the amount of reward delivered on each correct trial (n = 4 mice, 100% reward = 12μl). Mice perform fewer trials with larger reward volumes (roughly according to the red line of constant daily water intake (red: trials x reward = const line)). (b) Number of trials per vs. recorded day’s weight in a separate cohort (n = 20). There is a strong positive correlation between weight and number of trials performed, suggesting that animals are capable of regulating their own metabolic demands within AutonoMouse.

A key feature of AutonoMouse is that stable, reliable training can be achieved without using water restriction techniques. We demonstrate this by adjusting the amount of water each animal receives per trial. If animals are truly gaining water *ad libitum* in exchange for performing behavioural tasks, the number of trials performed should scale proportionally with the amount of water delivered per task. Indeed, increasing the water reward proportionally decreased the number of trials performed (Fig 5A). Thus, despite having the option to perform significantly more trials, animals only performed those trials needed to gain their required daily intake of water (number of trials x reward amount = constant). It should be noted, however, that decreasing water substantially below 12μl (<100% in Fig 5A) was not compensated sufficiently by additional activity. Furthermore, the average number of trials per day performed by an animal was related to its weight (Fig 5B). As trials in the system are initiated by the animals themselves, thissuggests that animals were capable of self-regulating their activity patterns to meet their metabolic demands within AutonoMouse (and that competition for access was not likely to be a confounding factor at least for reward sizes of 12 μl and above). This in turn allows the experimenter to adjust the number of trials that animals perform daily (e.g. equalize these numbers across animals) by adjusting individual water reward levels.

### Assessment of graded olfactory bulb lesions on olfactory discrimination

The large number of trials and tasks that can be acquired with AutonoMouse now allows us to tackle aforementioned behavioural questions more systematically. We investigated the extent of OB disruption required to produce complete anosmia, as well as phenotypes observed when OB challenge was below this threshold. We thus subjected a total of 29 animals in 3 cohorts to stereotaxically directed OB injections of N-methyl-D-aspartate (NMDA) in varying amounts to produce graded OB lesions. Volume microCT analysis confirmed that varying NMDA amount between 303 and 2125ng resulted in lesions of varying size up to an extent that only fragments of OB tissue were visible at the largest amount (S3 and S4 Figs). Quantifications of lesion size were made relative to the skull volume of each animal to control for bias based on skull size (S4 Fig).

**Fig 6:**
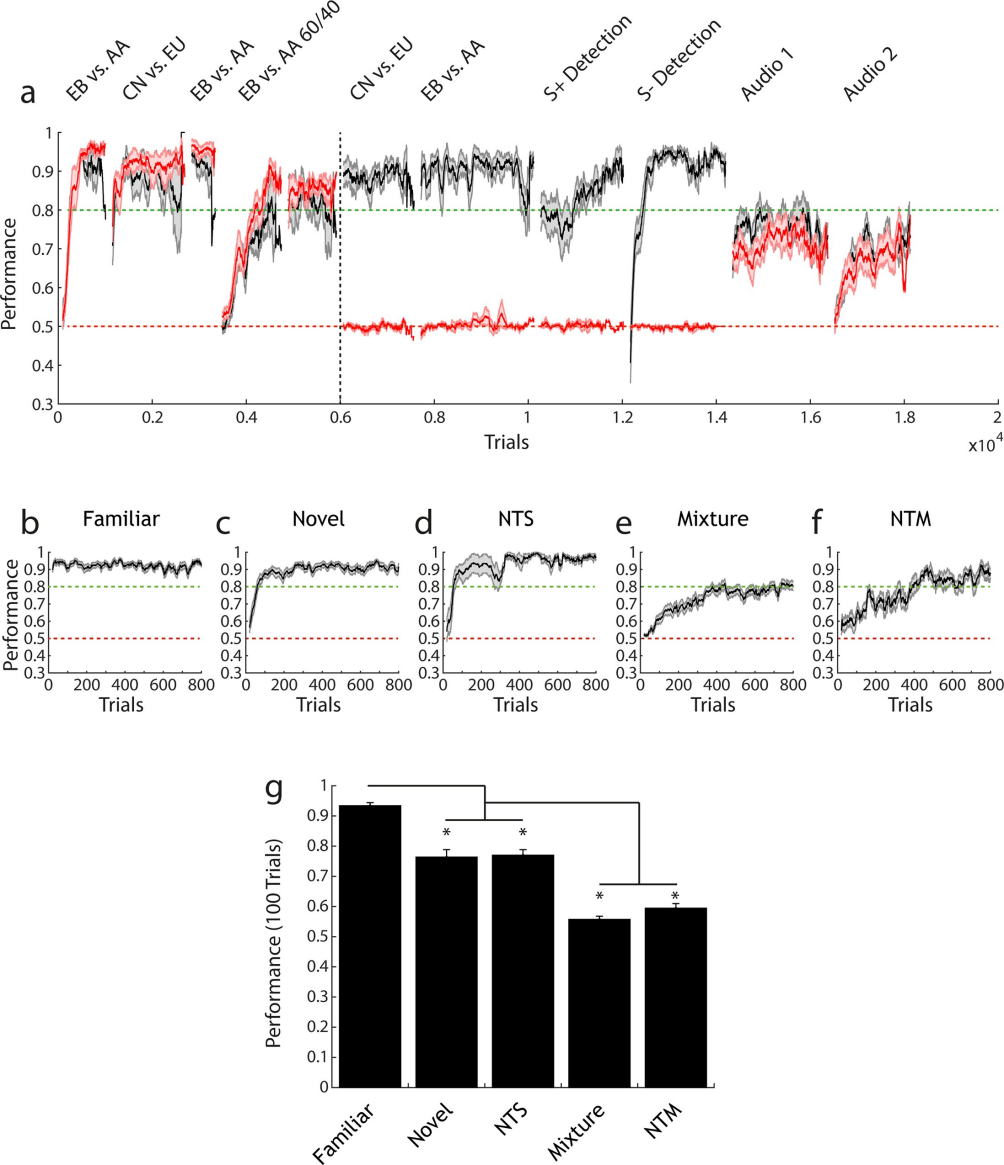
Large olfactory bulb lesions induce anosmia; task context affects performance rate (a) Performance (mean +/- sem) over several olfactory tasks for sham-injected (black, n = 6) and lesion animals (red, n = 8). Performance is calculated in a 100 trial moving average. Performance is shown before and after lesion induction (before and after black dotted line respectively). (b-g) Combined pre- lesion and sham performance for each distinct task context. All performance is shown calculated over a 20 trial moving average (mean +/- sem). (b) Familiar task: performing discrimination on a previously learnt odour pair (n = 38). (c) Novel task: odour pair has not been previously encountered (n = 32). (d) Non-trigeminal simple task: odour pair has not yet been encountered and both odours are non-trigeminally activating (n = 9). (e) Mixture task: discrimination between simultaneously presented odours in the ratio 60:40 vs. 40:60 (n = 31). (f) Non-trigeminal mixture task: same as in (e) but both odours are non-trigeminally activating (n = 9). (g) Performance in the first 100 trials (calculated over 20 trial sliding window) on each task type and statistically compared (1-way ANOVA with Tukey-Kramer correction for multiple comparisons, F = 65.13, p = 1.46x10^-28^). Novel and NTS task performance is significantly lower than familiar performance. Mixture and NTM task performance is significantly lower than all other tasks.

We first investigated lesion-induced anosmia in a cohort by training animals on a battery of odour discrimination tasks before and after OB excitotoxic (2125ng NMDA, n = 8) or sham lesions (1% PBS, n = 6) with a range of odour pairs (Cinn. = Cinnamaldehyde, ACP = Acteophenone, EB = Ethyl butyrate, AA = Amyl acetate, V = Vanillin, P = Phenylethyl alcohol, CN = Cineol, EU = Eugenol, 2H = 2-Heptanone). Both groups reached high levels of performance accuracy before lesion induction (Fig 6A). Sham injected mice recognized previously learned odour pair discriminations and quickly learned new odour pairs and detection tasks (Fig 6A). Mice with full NMDA induced OB lesions showed significantly reduced performance in all olfactory tasks (Fig 6A), with accuracy levels at no stage distinguishable from chance levels (t-test final task performance level vs. chance level, CN vs. EU; t = -1.2287, p = 0.26, df = 7; EB vs. AA: t = 0.2513, p = 0.81, df = 7). To confirm that lesions did not produce an inability to perform GNG tasks in general we assessed performance in a series of auditory discrimination tasks. Lesioned animals were able to perform auditory discrimination tasks as well as sham injected animals (t-test final performance level sham group vs. lesion group, Audio 1 0.3 vs. 3kHz: t = 0.2292, p = 0.82, df = 12; Audio 2 5 vs. 10kHz: t = 1.2998, p = 0.22, df = 12). Performance deficit was not limited to olfactory discrimination as lesioned animals also failed in odour *detection* tasks (Fig 6A, S+ detection / S- detection, t-test final performance level vs. chance, S+ detection: t = -0.0968, p = 0.93, df = 7, S- detection: t = -1.0037, p = 0.35, df = 7). Thus, extensive lesioning of both OB hemispheres resulted in seemingly complete anosmia.

It is presumed that certain tasks in the olfactory discrimination set should be more behaviourally demanding than others (e.g. learning novel odour pair vs. binary mixture discrimination [10,32]). To quantify this and rank-order different discrimination tasks, pre-lesion performance data for all animals was pooled according to task identity (Fig 6B–6G). Performance for a familiar odour pair was consistently higher than for other tasks. Novel general odour pair tasks (“Novel”, “NTS”) were performed with significantly lower accuracy in the first 100 trials (ANOVA with Tukey-Kramer correction for multiple comparisons, F = 65.13, p = 1.46x10^-28^); with binary mixture discrimination tasks performed at lower accuracy still. Thus, our battery of olfactory discrimination tasks were variably demanding to complete accurately.

**Fig 7:**
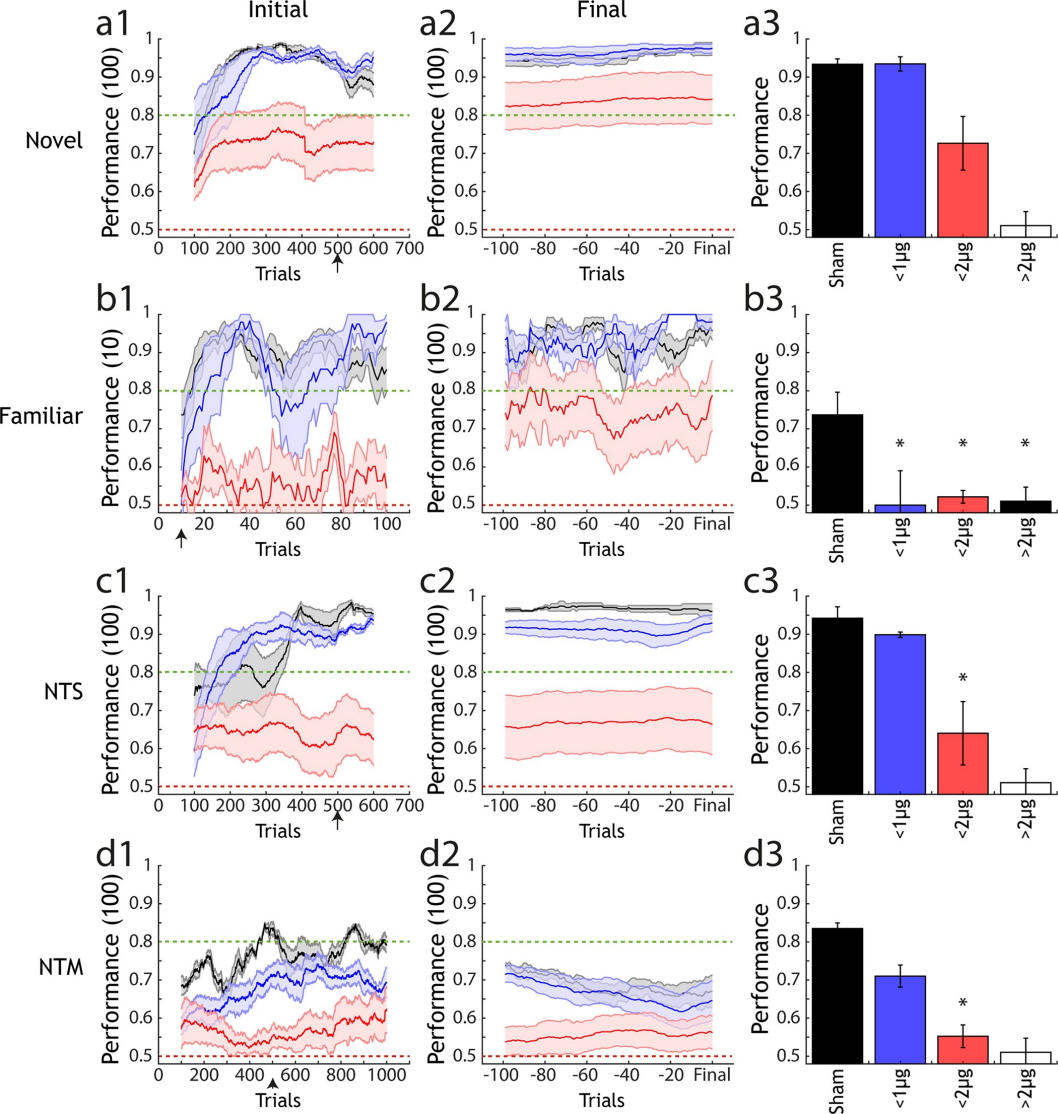
Analysis of performance across lesion groups and types of olfactory task. (a1) Performance of 3 lesion size groups (sham: black, n = 9; <1000ng NMDA: blue, n = 5; <2000ng NMDA: red, n = 7) in a novel odour discrimination task (mean +/- sem). Performance is calculated over a 100 trial moving average. (a2) Final performance in the same groups as (a1), performance is calculated for each animal with a sliding window of 100 trials from 100 trials before- up to the final trial performed for each animal in the task. (a3) Average performance (mean +/- sem) for each group after the number of trials indicated by the black arrow on the x-axis in (a1). Final unfilled bar indicates estimated performance for the anosmic group, based on data gathered for (d3). * indicates significantly different performance compared to sham under 1-way ANOVA with Tukey-Kramer correction for multiple comparisons. (b1)-(b3), (c1)-(c3) and (d1)-(d3) are as in (a1)-(a3) but for a familiar odour task, non-trigeminal simple task (NTS) and non-trigeminal mixture task (NTM). In (d1)-(d3) performance is calculated in a 10 trial sliding window as the crucial metric for a familiar task is performance in the first few trials, where animals must rely on recognition of the previously learned pair rather than ongoing task learning.

We next asked what odour discrimination capability remained in animals with less extensive lesions than those used to produce complete anosmia. Animals administered with smaller NMDA amounts (303.6–607.2ng NMDA), and therefore presumptively smaller OB lesions, readily learned to discriminate a novel odour pair (Fig 7A1). Both asymptotic performance as well as learning rate were indistinguishable from sham injected animals (Fig 7A3). Animals with larger lesions (1214–1669.8 ng NMDA) also showed above chance performance (Fig 7A1) but attained criterion level performance at a slower rate. Final performance was marginally less than the sham and small lesion groups though statistically indistinguishable (Fig 7A3).

Although all lesion groups (except “full lesion” animals that were anosmic, Fig 6A) were capable of performing simple binary discriminations of odours, when groups were presented with an odour pair learned prior to lesion induction (Fig 7B), a more subtle phenotype was observed. Performance was generally similar to the novel odour case with the small lesion group reaching comparable accuracy to sham animals and the large lesion group reaching consistent above-chance performance. In the early stages of the task where re-learning of the odour pair was unlikely to be a contributing factor on performance a substantial reduction in performance was already observed for the small lesion groups (relative to sham) (Fig 7B3). This difference was significant relative to sham animals in the first 10 trials of the task where performance of the small lesion group was also not statistically larger than chance. Moreover, for novel odour pair performance in the same trial window (first 10 trials) there was no significant difference in performance between the sham and lesion groups (ANOVA with Tukey-Kramer, F = 0.65, p = 0.54) suggesting that the deficit observed for a familiar odour task was specific to that context and not a result of different initial learning rates between the groups. The small lesion group then quickly regained comparable performance to sham animals within the first 20–40 trials of the task. This suggests that, for a relatively small OB lesion, the ability to quickly learn a new odour pair discrimination is largely unaffected but recognition of a previously learned pair is significantly diminished.

Mice were also trained to perform an additional simple binary odour discrimination with odours considered purely olfactory in that they activate the olfactory system alone and do not stimulate the trigeminal nerve [63–65]. This allowed us to determine the extent to which lesion group discrimination might be based on differential trigeminus activation between odours (Fig 7C). As with the other simple discrimination tasks, there was little difference between the small lesion group relative to sham after a sufficient learning period (Fig 7C2 and 7C3). However, in contrast to the case of a trigeminal-activating odour discrimination (Fig 7C) performance was significantly reduced for the large lesion group relative to sham (Fig 7C2 and 7C3) suggesting that there may have been a small contribution of trigeminal activation in task acquisition by the large lesion group in other simple odour tasks.

For non-trigeminal mixture discrimination (NTM) (Fig 7D), the large lesion group again performed significantly worse than sham controls (Fig 7D3). This suggests that although simple odour discrimination is unimpaired across all non-anosmic lesion groups, the additional complexity of mixture discrimination poses a significant challenge for an impaired olfactory bulb

These results indicate that a damaged OB can cope relatively easily with simple odour discrimination tasks and that tasks of this nature are not sufficient to reveal the phenotype change associated with this damage. By looking in more detail at odour pair recognition, and ability in the case of increasing task demands such as mixture discrimination, significant impairments can be observed.

## Discussion

### AutonoMouse

The design of AutonoMouse enables large-scale, systematic behavioural experiments through high-throughput, fully automated training of multiple animals simultaneously. Our results show that the system can train large cohorts of mice, producing 1000s of trials per day across these animals and motivating them to perform without resorting to methods such as severe water restriction. Crucially, the automated nature of the system largely eliminates the need for experimenter presence and intervention during behavioural trials. Virtually all behavioural methods (including this one) require an experimenter to be present at some point, however in our implementation this is restricted to a few touch points during a several month long study. Consequently, mice housed in the system are subject to fewer external stressors such as manual handling. For experimenters, this means that relatively little time is needed for monitoring ongoing experiments and it is thus completely feasible to run experiments on several systems in parallel. One potential downside of this approach (minimal on-line experimenter monitoring of animal presence / performance) is that scenarios may occur where animals are present in the behaviour port and are triggering trials but are not engaged in the task. This does appear to happen occasionally in our data, for example where animals appear to perform >1000 consecutive trials in a single session (Fig 2B, inset). However, given that large stretches of trials such as these are performed relatively infrequently and that reliable, accurate performance was observed in almost all discrimination tasks it is not expected that this scenario has a major effect on the utility of AutonoMouse in efficient operant conditioning. In future experiments, this scenario could be avoided by halting trial progression completely in cases where animals cease to lick entirely for a prolonged stretch of trials.

Animals in the system quickly and reliably acquired the ability to perform olfactory discrimination tasks with accuracy levels generally comparable or well above criterion levels commonly used in neuroscience research with similar behavioural tasks [30,50,66,67] (S6 Fig). Overall, experimenter-animal interactions are minimal and could be eliminated completely if e.g. automatic weighing is integrated [26].

Beyond direct behavioural analysis, AutonoMouse could also be used to prepare animals for head-fixed behavioural paradigms. Head-fixed behaviour is an essential technique in systems neuroscience that permits simultaneous circuit interrogation with quantitative behavioural readouts. A limitation of this technique as it is commonly implemented is that is can be highly time-consuming to habituate and train animals in head-fixed apparatuses (7–14 days to criterion per mouse in whisker behaviour:[9]; >4 days in olfactory discrimination including habituation: [68]). While voluntary head fixation experiments [21,69] can partially alleviate these challenges for imaging experiments, AutonoMouse could increase the efficiency of this process by training animals in the intended behavioural task, building up a ‘stock’ of trained animals through simultaneous training. These animals could then be transferred to a head-fixed setting on achieving a reliable criterion level, circumventing the laborious task of manual training.

The general design principle of AutonoMouse can be applied to a range of experimental requirements, giving it some advantage over current RFID based mouse behaviour systems generally designed for specific tasks (e.g. IntelliCage, [51]). The open-source design is compatible with operant conditioning in any number of sensory modalities. Olfactory stimulus generation could be replaced with, for example, a screen for visual training; or—as introduced in Fig 6 –a speaker for auditory training. Introduction of a second lick port would allow for implementation of 2-alternative forced choice paradigms. The behavioural staging area of AutonoMouse could also be modified to allow for different training paradigms. For example, the access tunnel could open into a wide-field arena or maze for testing navigational ability [52]. Furthermore, the 24/7 operating nature of the system means that it could be suitable in studies in which long-term monitoring is crucial to the experimental findings, e.g. sleep studies.

The control software for AutonoMouse allows for installation of and acquisition from extra sensors with relative ease. In future experiments, a respiration monitor (such as a pressure sensor or infra-red camera) could be installed to monitor sniffing during olfactory discrimination. Recent technical advances have seen the advent of a number of neurophysiological techniques moving to compact wireless technology platforms, e.g. head-mounted optogenetic stimulation [70,71] and neural recording [72–74]. Using these devices in conjunction with the high-throughput nature of AutonoMouse's behavioural data collection would comprise a powerful technique for general neuroscience research. Moreover, as the system itself is adaptable to a number of behavioural tasks, and the software generated schedules can easily be shared between groups AutonoMouse and systems like it also have the potential to increase standardisation of behavioural experiments across labs. To promote this we have provided a complete description and construction manual in the appendix.

### Assessment of graded olfactory bulb lesion effects

In this study we demonstrate the utility of AutonoMouse by applying it to a systematic investigation into the effect of lesions of the OB on olfactory discrimination performance. The results of this investigation address a recurring interpretation in the literature regarding the effects of OB lesions and the relevance of their results in understanding the mechanism of olfactory perception. The results of a number of lesion studies [41–44] have indicated that there remains a significant ability to discriminate odours after extensive damage to the OB. These results and their interpretations [45–48] have therefore questioned the prominence of spatial / indentity coding in mammalian olfaction. The use of AutonoMouse allowed us to revisit these experiments in an efficient manner—probing a range of OB lesion sizes, task contexts and odour pairs. The largest bulbar lesions tested resulted in anosmia (Fig 5A), though performance in simple discrimination tasks remained intact with large but less extensive lesions (Fig 7A and 7B). Significant reductions in performance were observed for the largest non-anosmic group only for non-trigeminal discrimination tasks (Fig 7C and 7D). For small lesions, significant deficits in performance were observed only for familiar odour tasks in which odour recognition was the tested variable (Fig 7B).

That odour recognition is the only behaviour consistently affected for all lesion extents suggests that retention of odour identity perception is particularly sensitive to OB disruption. The reduction of performance in this task was not due to inability to perform general odour discriminations as all groups with odour recognition deficits were largely still able to learn novel odour pair discriminations (Fig 7B). This is in agreement with previous findings [50] where it was also reported that transient decreases in performance accuracy occur for odour recognition tasks (after nasal epithelium lesioning) are followed by rapid re-learning. Our results indicate that the same effect is observed for bulbar lesions, reiterating that odour recognition is based on stimulus input matching to previously learned perceptual ‘templates’. Representation of these templates is a function of the olfactory bulb and they are therefore degraded by lesioning—resulting in perception of a previously learned odour as novel. The ability to re-learn this apparently novel odour is largely unaffected, thus the rapid increase in performance accuracy within only a few 10s of trials.

Simple odour discrimination was only significantly impaired once non-trigeminal odour pairs were introduced, suggesting some odour pairs might be discriminable in part due to differential activation of the trigeminal nerve. This could account for some of the discrepancies in previous studies that observe no loss of discrimination ability even with extensive lesions. Intact performance in these cases could be based on trigeminal rather than olfactory processing. It should be noted, however, that the largest OB lesions did result in complete anosmia suggesting that trigeminal processing is not sufficient for odour discrimination. We did not image the trigeminal nerve after lesion induction but given that the spread of tissue damage was relatively local in our lesions (S1 Fig) it is unlikely that our method induced damage in the trigeminal pathway. Furthermore, this nerve is well separated anatomically from the OB in rodents [75] although we cannot exclude effects on the trigeminal nerve through ethmoid collaterals in the olfactory bulb [76].

Regarding the interpretation that odour discrimination in general is left intact after major disruption of the OB [45–48]: it is true that relatively large lesions of the OB do not impair simple olfactory behaviours; but more complex tasks involving recognition, mixture discrimination and discrimination of non-trigeminal stimuli are readily affected by even minor disruption of the OB. This was revealed in this study by a systematic approach to analysing behaviour over a range of tasks. The results suggest that OB circuitry required to discriminate between pure odours is relatively redundant, but the failure of animals with small lesions to instantly recognise previously learned odours suggests that retention of odour identity is non-redundant in the olfactory system. We have focused here primarily on perception of odour identity, but it is possible that OB damage also affects olfactory sensitivity and the ability to detect low concentrations of certain odours. Perceptual thresholds for odours can be determined using the same go/no-go paradigms that we employ here [77] and it is feasible that AutonoMouse could be used in future to extensively probe these thresholds before and after OB challenge.

Since mice are capable of detecting extremely low levels of some odorants [77] we endeavoured to ensure that perception of any olfactometer contaminants was not responsible for behavioural performance. In Fig 3C, a small residual odour signal was observed during opening of clean air valves. It is very likely that this residual signal arose at the output level of the olfactometer and was therefore common between trials and could not be used to discriminate S+ from S- stimulus. This is because opening of the final valve alone (without any input level valves) also produced a small PID signal comparable to that of when empty input valves were added. The design of the olfactometer (Fig 3A) also ensured constant airflow through the normally open valves of the input so any input-level contamination should have been constantly purged to some degree.

Nevertheless, we implemented a number of controls to ensure residual odour signals could not account for any of the behavioural performance observed. In addition to minimizing any contamination through extensive washing / purging steps (see Methods), an ongoing control throughout training was to begin each new odour discrimination task with a subset of odour valves and then introduce new, previously unused odour valve pairs after some 100s of trials of training. If animals were using unintended cues to learn the discrimination tasks then performance should drop after introduction of these new valves, but we observed no significant changes in performance (Fig 3E, 3F and 3H). In some cases we also repositioned the odour bottles within the olfactometer as a further control. After this repositioning, odour input could be remapped at the software level in order to deliver the same stimuli as before–but the physical switch of odour bottles would cause any existing olfactometer contaminants to be delivered in a different pattern. Therefore, if animals were using olfactometer contaminants to learn the tasks then performance should drop after this control, but again we observed no difference in performance (Fig 3G).

### Conclusion

Here we have presented a fully automated, high-throughput system for self-initiated conditioning of group-housed mice over periods of several months. We have used this system to demonstrate that odour discrimination in general is indeed robust to even most extensive disruptions; already small olfactory bulb lesions, however, impair odour detection, and generally increasing lesion size gradually impairs olfactory performance.

The modular nature and open-source design of AutonoMouse, as described in detail in the accompanying appendix, should allow for similar systematic assessments across neuroscience research areas and increase the robustness and efficiency of large-scale behavioural phenotyping efforts.

## Materials and methods

### Ethics statement

All animal experiments were prospectively approved by the local ethics panel of the Francis Crick Institute (previously National Institute of Medical Research) and UK Home Office under the Animals (Scientific Procedures) Act 1986 (PPL: 70/7827). All mice were C57BL/6 and obtained from Charles River (Basel, Switzerland) or by in house breeding. Both male and female mice were used (see below), starting transfer into AutonoMouse from 4–6 weeks of age. All reagents were obtained from Sigma-Aldrich unless noted otherwise. For minor procedures (RFID implant), mice were briefly anaesthetised with isoflurane. For major procedures (lesion induction, perfusion), mice were anaesthetised with a ketamine/xylazine mix. At experiment termination, where perfusion was not performed, mice were sacrificed via carbon dioxide inhalation followed by cervical dislocation as a secondary method.

### AutonoMouse structure

A detailed manual for the construction and operation of the AutonoMouse system can be found in the appendix. A repository containing design files for the system hardware can be downloaded from https://github.com/RoboDoig/autonomouse-design. The main control software can be found at https://github.com/RoboDoig/autonomouse-control, and the schedule generation program at https://github.com/RoboDoig/schedule-generator.

In brief, the home cage chamber of AutonoMouse was constructed from aluminium profiles (*MayTec Aluminium Systemtechnik GmBH*, *Dachau*, *Germany*) and walled with clear acrylic panels. The cage dimensions were 52x62x17cm. The cage contained floor-bedding (*Alpha Dri*, *LBS Biotechnology*, *UK*), environmental enrichment (running wheels, tunnels, soft bedding, ‘homes’, chew blocks) and a metal cage containing diet. A pre-chamber area constructed from acrylic panels was connected to the home cage by a wooden ramp. The pre-chamber was connected to the behaviour port via an acrylic tunnel. Access to the tunnel/behaviour port was controlled by a swing door, actuated by a rotary magnet (*GDRX 050 X20 D02 24V 100%*, *Magnet-Schultz*, *Woking*, *UK*) and controlled with custom electronics. Infra-red (IR) beam sensors lined the walls of the access tunnel to detect animal presence. All behaviour was monitored in the behaviour port, which consisted of a custom plastic open faced enclosure housing an IR beam emitter/detector (*PIE310/PID310D*, *Kodenshi*, *Nagoya*, *Japan*), an RFID detector coil, a lick port, and some stimulus delivery device installed according to the desired behavioural task (e.g. odour port, speaker).

### AutonoMouse control modules

#### Lick module

Animal licking and water delivery was via a lick port housed in the behaviour port. The lick port was a hollow metal tube, open on the side facing the animal and connected to a water reservoir and gear pump (*MZR-2521*, *Harton Anlagentechnik GmBH*, *Alsdorf*, *Germany*) on the other side. The gear pump was controlled with a micro-controller (*S-ND*, *Harton Anlagentechnik GmBH*, *Alsdorf*, *Germany*) which could receive analog input via the AutonoMouse software to drive speed and duration of water delivery. Lick contact with the port was detected with custom electronics (see lick-detector.sch in the ElectronicsModules section of the autonomouse-design repository and Fig K in S1 Appendix).

#### IR module

Inputs from the IR beams were managed with custom electronics (see ir-logic.sch in the ElectronicsModules section of the autonomouse-design repository and Fig L in S1 Appendix). This module powered and received input from IR beam detectors and relayed the on-off logic to other modules.

#### Door module

Actuation of the door was controlled with custom electronics (see door-close.sch in the ElectronicsModules section of the autonomouse-design repository and Fig M in S1 Appendix). This module received input from IR sensors and actuated the rotary magnet according to sensor input. When an animal was present in either the access tunnel or behaviour port, an IR beam was broken and the door was closed ensuring that only 1 animal had access to the behaviour port at a time. IR beams did not cover the portion of space closest to the access door and animals could therefore leave the tunnel by moving close to the door. In this position, no IR beams can be activated and the door would release and open via a spring that held the door normally open.

#### RFID module

The identity of the animal in the behaviour port was read out with an RFID detector and decoder (*Trovan LID-665 OEM Single Coil Compact Decoder*, *RFID Systems Ltd*., *Yorkshire*, *UK*). The decoded RFID was relayed to the software via a serial port.

#### Flow control

Olfactometer flows for input lines were controlled with a mass-flow controller (*MKS 1179C Mass-Flo*, *MKS*, *Andover MA*, *USA*). Purge of the carrier stream was controlled with an air-pressure regulator (*Air-regulator*, *Sigmann Electronik GmBH*, *Hüffenhardt*, *Germany*).

#### Digital / analog control and acquisition

All sensor data, digital I/O control and analog I/O control was via a peripheral component interconnect (PCI) data acquisition (DAQ) device (*PCI-6229*, *National Instruments*, *Austin TX*, *USA*) with a Bayonet Neill-Concelman (BNC) interface (*BNC 2090A*, *National Instruments*, *Austin TX*, *USA*), except for RFID reading and day-night cycle control which was via direct serial interface between an LED strip and a PC.

### Animal preparation

All animals taking part in a particular AutonoMouse cohort were immediately housed together in a group cage after weaning to avoid disruption of social hierarchy and aggression later in the experiment [55,56]. Animals (either male or female cohorts) underwent RFID implant surgery and were transferred to AutonoMouse at 4–6 weeks of age.

### RFID implant

Before being housed in AutonoMouse, all mice underwent an RFID implant surgery such that they could be individually identified by the system. Mice were anaesthetised under isoflurane (induction: 5% in O_2_ 2l/min, maintenance: 2%) and placed on a heat pad for maintenance of body temperature during the surgery. The fur around the base of the neck and scruff was shaved away and the skin cleaned with chlorhexidine (1%) and then dried with a sterile swab. A pre-sterilised needle (*IM-200*, *RFID Systems Ltd*., *Yorkshire*, *UK*) containing an RFID chip (*ID-100B*, *RFID Systems Ltd*., *Yorkshire*, *UK*) was then loaded onto a plunger and inserted into the loose skin at the base of the neck. The plunger was used to push the chip out of the needle before removing the needle, leaving the RFID chip implanted under the skin. Forceps were then used to pinch shut the incision made by the needle and medical superglue (*Vetbond*, *3M*, *Maplewood MN*, *USA*) was applied to seal the wound. Animals were returned to an individual cage for 10 minutes following the surgery to recover from anaesthesia and for the superglue to dry. Once righting reflex was regained and the wound was confirmed as properly sealed the mouse was returned to the group cage with its cohort. Very rarely (1/67 of mice undergoing the surgery) an animal might display some skin irritation over the RFID implant wound. In this case topical ointment (*Dermisol*, *Zoetis*, *Surrey*, *UK*) was applied daily until the irritation receded.

### Lesion induction

Prior to surgery all utilised surfaces and apparatus were sterilised with 1% trigene. Surgical instruments were sterilised in an autoclave. Surgery was carried out with standard aseptic technique.

A glass injection pipette, pulled on a capillary tube puller (*P-1000*, *Sutter Instrument*, *CA USA*) and broken off to approx. 15μm diameter was back-filled with either NMDA (*Sigma-Aldrich*, *St*. *Louis MO*, *USA*) (10mg/ml diluted in 1% PBS) or 1% PBS and inserted into the injector apparatus (*Nanoject II*, *Drummond Scientific*, *PA USA*). Mice were anaesthetised with ketamine/xylazine solution via intraperitoneal injection (Vetalar/Rompun; 80mg/kg / 10mg/kg) and placed on a warm heat pad. Depth of anaesthesia was monitored throughout the procedure by testing toe-pinch reflex. The fur on the skull extending from the base of the head to the tip of the nose was shaved away and cleaned with 1% clorhexidine scrub. Mice were then placed on a thermoregulator (*DC Temp*. *Controller*, *FHC*, *ME USA*) heat pad controlled by a temperature probe inserted rectally. While on the heat pad, the animals were inserted into a stereotaxic frame (*900LS*, *Kopf Instruments*, *CA USA*) and a sterile surgical cover (*Buster op cover*, *Kruuse*, *Langeskov*, *Denmark*) was placed over the body of the animal. The scalp was incised and held away from the skull with arterial clamps and two craniotomies were made with a dental drill (*Success 40*, *Osada*, *Tokyo*, *Japan*) above the 2 olfactory bulb hemispheres. The craniotomies were covered with 1% phosphate-buffered saline (PBS) to prevent drying of brain tissue during the surgery. Depending on the desired lesion size, injections of either N-Methyl-D-aspartic acid (NMDA, *M3262*, *Sigma-Aldrich*, *St*. *Louis MO*, *USA*) or PBS were made to several injection sites in the bulbs (see Table 1).

### Odour delivery

Odour stimuli were delivered with a custom-built 8 channel olfactometer (see Fig 3) with two parallel input lines. Parallel lines were controlled separately and one odour input from each line could therefore be delivered to the odour carrier air stream simultaneously. Odour concentration delivered to the main odour carrier air stream was controlled by varying the flow and pressure levels in the parallel input lines. The stimulus given to the behaving animal was controlled by switching between a clean air and odourised air flow line via a 5-way solenoid valve (*VK3210*, *SMC*, *Tokyo*, *Japan*).

Where pure odours were delivered to the animal (e.g. in a pulse of EB), the final odour stimulus was generated by first generating a ‘pre-pulse’ from the input mass-flow controllers (short burst of pressure at high flow to pressurise the system) and then triggering (at random) a set of valves from each parallel input line connected to the odour source of choice. Where binary mixtures of odours were delivered (e.g. in an EB/AA 6/4 pulse) the valve choice was also randomised but each input line delivered a separate odour. Each input line contained two S+ sources and two S- sources. Therefore, the sequence of valves used to deliver either an S+ or S- stimulus had 4 possible combinations for pure odour stimuli, and 8 possible combinations for binary mixtures. Chosen at random, these combinations ensured that animals were unlikely to learn to discriminate the noise of valve opening rather than odour stimulation.

To ensure that the odour stimuli were the only salient signals that were learned in the discrimination task, control stimuli were designed in which the number of active valves was gradually increased (Fig 3E–3H). Initially, animals would be trained on only 4 valves (1 odour 1, 1 odour 2, 2 blank), typically for several hundred trials. At some point during training, 2 new valves were introduced to stimulus production and training continued. Finally another 2 valves were added and the full set of 8 was used to generate stimuli. The transition between valve numbers was automated so there was no additional time delay from one case to the other. By comparing performance before and after introduction of new valves, we could confirm that mice were truly using only the odour signals to discriminate. If performance dropped after introduction of the new valves it was an indication that some extraneous cue to do with e.g. the noise of valve switching was being learned in addition to or instead of the odour signal.

### Experiment initiation and maintenance

After being implanted with an RFID chip, animals were weighed and transferred into the common home cage of AutonoMouse. In general, the first behavioural task assigned to all animals was a pre-training task designed to train animals to reliably gain their water intake from the behavioural port, and in which reward could be gained on all trials:

1. Water delivered as soon as animal detected in behaviour port (10 trials)
2. Animal must lick at least once to gain water reward once detected in behaviour port (50 trials)
3. The percentage of total trial time (2s) that the animal must lick to gain a water reward is increased (up to 10% of trial length) (100 trials)

Each water reward was initially 15μl. This was adjusted to 10–30μl depending on animal performance (to ensure all mice performed roughly the same number of trials per day). During performance of these trials, animal weight was monitored daily, in addition to number of trials performed, to ensure that animals were indeed gaining their necessary daily water from the water rewards in the behaviour port. If any animal dropped more than 5% in weight from the previous day, it was removed from the system and given water *ad libitum* for 10 minutes before being returned to the system. Any animal that consistently performed <100 trials per day or consistently dropped in weight (more than 2 days in a row) was isolated in the behaviour port and manually given water rewards from the lick port. Any animal that still dropped in weight or performed <100 trials per day after this treatment was removed from the cohort (<10% of all animals were removed due to low performance).

For the first two weeks of any AutonoMouse experiment, animal weights were checked daily to ensure health status of the. After two weeks, weight was manually checked more infrequently (every 4–5 days) but total trials performed was monitored daily to ensure animals had all performed >100 trials in the last 24 hours. Any animals not meeting this criterion were given water *ad libitum* for 10 minutes and then returned to the system.

The system was designed for bedding exchange without direct human-animal contact: A panel beneath the cage was removed to allow loose bedding to fall through a mesh into a removable drawer. This was routinely performed when bedding was soiled (<1 per week). Meanwhile, bedding in nests inside mouse houses could be left unperturbed. Afterwards, the panel was replaced and bedding refilled from the top. During this procedure–typically occurring during the day time–mice would either sleep in their nests or reside in the upper behavioural area. Thus minimal disturbance and no direct human-mouse contact were needed. Mice could be confined to the home cage via an access panel (Fig G in S1 Appendix) to allow cleaning of all parts of the upper chamber without human-animal contact.

For “deep cleaning” the AutonoMouse system animals were transferred to a temporary group cage along with any loose bedding. Any areas with animal contact were removed and soaked in disinfectant (*Trigene*, *Ceva*, *Glenorie NSW*, *Australia*), cleaned and dried. The (AutonoMouse) cage floor bedding was removed and replaced using the quick-removable bedding tray (Figs F, J in S1 Appendix). Animals were then transferred back into the system along with loose bedding.

### Task structure

All tasks following the pre-training phase followed a standard go/no-go training paradigm. Animals were presented with either S+ rewarded odour or S- unrewarded odour (reward is reversed for roughly half the experimental group, e.g. in a group of 20 learning an EB (ethyl butyrate) vs. AA (amyl acetate) task, 10 are trained on EB as the S+ stimulus and 10 are trained on AA as the S+ stimulus) triggered by animal presence in the behavioural port. A ‘response’ was defined as a detected lick in 3 or more equally sized time-quarters (response quarters) during stimulus presentation, whereas a ‘rejection’ was defined as licking that fell in 2 or fewer response quarters (including no licking detected). A water reward could be gained by licking in at least 3 of the response period quarters following S+ odour presentation. Licking in at least 3 of the response period quarters during S- presentation resulted in an increased ‘timeout’ inter-trial interval (8-12s), in all other response cases the inter-trial interval was 4s and no water reward was delivered. All task sequences were designed such that no more than 3 S+ or S- trials could occur sequentially, and the ratio of S+ to S- trials in each block of 100 trials was 1:1. Various kinds of discrimination tasks were presented to the experimental cohort. The terminologies, structure and primary purposes of these tasks are listed below:

#### Initial

The “initial” task was the first olfactory discrimination task presented after pre-training was complete. The purpose of this task was primarily to determine that all animals were capable of olfactory discrimination, and served as an initial version of the “novel” task.

#### Novel

A “novel” task was any olfactory discrimination between two pure odours in which the odours had never been previously presented to the animal. The purpose of this task was to determine the speed of task acquisition and confirm ability to perform discrimination for multiple odour pairs.

#### Familiar

A “familiar” task was any olfactory discrimination between two pure odours in which the animal had previously performed a discrimination task with the same two odours. The purpose of this task was to probe recognition and memory of acquired task learning.

#### Non-trigeminal simple (NTS)

An “NTS” task was any olfactory discrimination between two pure odours in which the two odours were non-trigeminally activating (vanillin and phenylethyl alcohol, [65]). The purpose of this task was to dissect out any contribution to learning and odour detection from stimulation of the trigeminal nerve.

#### Mixture

A “mixture” task was an olfactory discrimination in which animals were asked to discriminate between mixture ratios of two odours. For example, S+ might be odour 1 and odour 2 mixed together in a 60%:40% ratio, and S- might be the same odours in a 40%:60% ratio. The purpose of this task was to be a more behaviourally demanding version of olfactory discrimination.

#### Non-trigeminal mixture (NTM)

An “NTM” task was the same as a mixture discrimination task, but both odours were non-trigeminally activating. The purpose of this task was both to be a more behaviourally demanding version of olfactory discrimination while dissecting out any contribution to learning and detection from stimulation of the trigeminal nerve.

#### Auditory

In an “auditory” task, animals were asked to discriminate between two pure audio sine waves at different frequencies. The purpose of this task within this experimental context was to ensure that any changes in olfactory discrimination performance were due to changes in olfactory ability rather than changes in general ability to perform go/no-go (GNG) tasks.

#### S+ / S- detection

In a “detection” task, animals were asked to discriminate between an odour and clean air. This discrimination was either performed with the odour as S+ (S+ detection), or with the clean air as S+ (S- detection). The purpose of this task was to determine an animal's ability to simply detect an odour, rather than discriminate between two odours.

#### Task switching

When switching between tasks or changing installed odours, new odour bottles were used and the olfactometer was purged of any remaining contaminants by performing several hundred ‘dummy’ trials with empty bottles in which air was continually flushed through the olfactometer. Furthermore, on the switch to a new task S+ and S- odour positions were always changed such that if any residual odour from the previous task was present it could not be immediately informative of discrimination choice in the new task. Generally, residual odour that despite extensive purging might still be present in the system (e.g. Fig 3C) would be accounted for by residual odour in the *output* of the olfactometer (since signal is seen for opening of the final valve without any input-level valves open) and thus common for all stimuli and not informative about the discrimination task. Most importantly, switching from used valves to previously unused valves within a training paradigm (Fig 3F–3I) tests whether for a given discrimination tasks animals do use any unintended cues.

#### Training schedules

Over the course of the lesion study, 3 different cohorts (1: n = 6 female; 2: n = 14 male; 3: n = 9 male) underwent a set of behavioural tasks shown in Tables 2, 3 and 4.

**Table 4:** Training schedules (group 3).

Task (Group 3)	Odours / Hz	Task type
1	EB vs. AA	Initial
2	CN vs. EU	Novel
3	EB vs. AA	Familiar
4	N/A	Odour block
5	EB vs. AA	Mixture
Lesion		
6	CN vs. EU	Familiar
7	ACP vs. 2H	Novel
8	V vs. P	NTS
9	V vs. P	NTM

As in Table 1 for cohort 3 (n = 9). Task 4 is an odour diversion task (see Fig 3H) intended as a control to ensure animals were truly using odour information to perform discrimination

### MicroCT imaging

In some cases, the brains of mice in the experimental cohort were imaged using x-ray CT imaging to determine the extent of OB disruption induced by the lesion / sham surgery. The CT imaging method was based on a previously described protocol [78].

Mice were deeply anaesthetised with ketamine/xylazine solution via intraperitoneal injection (Vetalar/Rompun; 80mg/kg / 10mg/kg) and sacrificed by transcardial perfusion using 1% PBS clearant and 7.5% paraformaldehyde (PFA) perfusative (diluted with 1% PBS). The head was separated from the body and left to soak in a 40ml container containing 20ml Iodinated PFA solution (150mg/ml iodine–(*Niopam 340*, *Bracco*, *Milan*, *Italy*) diluted in 7.5% PFA).

After a minimum of 15 days soaking at 4°C the heads were transferred to custom made holders with attachments for placement in a microCT scanner (*SkyScan 1172*, *Bruker*, *Kontich*, *Belgium*). A scan of the olfactory bulb area was made using 70kV x-ray source power with an aluminium and copper filter at pixel resolution of 8.6μm. Ring artefacts were reduced by introduction of random movement into the head rotation during the scan. Coronal image sections were reconstructed from the scan using the SkyScan NRECON software.

### Software

AutonoMouse was controlled with custom Python software for building trial schedules, designing experiments and delivering these experiments to mice housed in the system. The main codebase and dependencies are available from the following repositories:

https://github.com/RoboDoig/autonomouse-control

https://github.com/RoboDoig/schedule-generator

https://github.com/RoboDoig/pypulse

https://github.com/RoboDoig/daqface

All analyses and figures were produced with MATLAB (*Mathworks*, *Natick MA*, *USA*) with custom written code.

### Analysis and statistics

Where behavioural performance is referenced, this was calculated as ((S+ correct / total S+) + (S- correct / total S-)) / 2. This calculation was used in order to weight all performance measures by the ratio of S+ vs. S- trials in an analysis window, to avoid biasing performance upward in cases where e.g. the analysis window was small and contained a higher fraction of S+ trials.

Unless otherwise specified in the test, all ANOVA tests were 1-way with Tukey Kramer multiple comparison tests. Correlation coefficients and significance levels were determined with the Pearson correlation coefficient test. All t-tests were 2-tailed. Unless otherwise specified, all specified confidence intervals in the main text (+/-) refer to standard deviation around the mean.

## Supporting information

S1 Fig Differences in performance for AutonoMouse cohort sizes. (a) Average trials per day for each animal plotted against the group size (number of animals) in which the animal performed. There is a significant negative correlation between group size and daily trials performed for each animal. (b) Fraction of trials performed each hour analysed as in Fig 2D for a cohort of n = 9 mice (top) and n = 24 mice (bottom).
(TIF)

S2 Fig Performance is correlated with daily trials performed, but not with average stretch length of those trials (a) Mean performance of all lesion study animals (n = 29) against average daily trials performed. Average daily trials is significantly positively correlated with mean performance (R = 0.5329, p = 0.0029). (b) Mean performance of the same animals against mean of length of consecutive trials performed. There is no significant correlation between mean performance and mean consecutive trials (R = -0.0787, p = 0.6847).
(TIF)

S3 Fig Excitotoxic olfactory bulb lesions. MicroCT images from mice injected with varying amounts of NMDA into the olfactory bulb (Sham: 0ng, S: 303.6ng, M: 607.2ng, L: 1214ng, XL 1669.8ng, XXL: 2125ng). Images are reconstructed coronal sections from a whole mouse head, starting at 0mm from bregma, to 1–1.3mm anterior from bregma (roughly the olfactory bulb injection site). Images are inverted such that darker regions correspond to more x-ray absorbent areas (e.g. skull, teeth, soft tissue absent areas where contrast agent has pooled). Bottom row: codes in brackets indicate RFID of animal used as representative example.
(TIF)

S4 Fig Example of the procedure used to characterise intact bulb volumes from CT images relative to skull volume. (a) Row of images shows example reconstructed coronal CT slices in rostral to caudal direction (left to right) in the olfactory bulb region caudal to bregma. (b) Same example images as in (a) after skull volume has been manually defined by tracing the outline in the stack of slices. Grey volume shows reconstructed skull volume around the bulb. (c) Same images as in (a) after bulbar volume has been defined manually. Pink volume is reconstructed bulbar volume. (d) After manually defining intact bulb volume and skull volume, bulb volume relative to skull volume is plotted against injected NMDA for 9 CT imaged mice. There is a significant (and with one exception very tight) negative correlation between relative bulbar volume and injected NMDA (Pearson correlation coefficient R = -0.7005, p = 0.0356), showing that larger volumes of injected NMDA have a graded, progressive effect on olfactory bulb damage.
(TIF)

S5 Fig Quality of learning during olfactory discrimination in AutonoMouse related to time taken to perform trials. (a1) Number of hours taken to perform a target number of trials (1^st^ 500) during initial odour pair learning vs. novel odour pair learning (n = 29). Hours to target are significantly correlated across the two task types (R = 0.84, p = 1.22x10^-8^). (a2) Performing animals are classified according to the rate at which they perform trials. For 4 task types (initial, novel, mixture, familiar) the time taken to perform the 1^st^ 500 trials in each was averaged for each animal. Fast (green, n = 17) animals are those with mean time to target completion greater than the mean time to completion over all animals and slow (red, n = 12) animals are those with mean time to target completion less than this average. Performance is shown for both groups on initial odour pair discrimination. (a3) Mean maximum performance in the initial odour pair discrimination for the same groups in (a2). (b1) Hours to target for novel odour pair vs. mixture learning (R = 0.85, p = 5.48x10^-9^). (b2) Performance for the fast and slow groups in a novel odour pair task. (b3) Average maximum performance in the novel odour pair task. (c1) Hours to target for initial vs. mixture learning (R = 0.86, p = 1.54x10^-9^). (c2) Performance for the fast and slow groups in a mixture discrimination task. (c3) Average maximum performance in the mixture discrimination task.
(TIF)

S6 Fig Comparison of AutonoMouse behavioural performance with previous semi-automated approaches. a) Learning progression for different experimental groups from different previous studies on the first odour pair discrimination introduced (initial). Performance calculated in bins of 100 trials. AutonoMouse n = 29; Shimshek, 2005 n = 9; Abraham, 2004 n = 6; Abraham, 2010 group 1 n = 9; Abraham, 2010 group 2 n = 13. b) Top: Boxplots of maximum performance obtained in several odour discrimination tasks for the same studies and animals shown in a). Significance star indicates cases where maximum performance was significantly lower than all other performance cases (1-way ANOVA, Tukey Kramer, F = 8.1, p = 6.2x10^-12^). Bottom: Bar plots (mean +/- sem) of trials to criterion–defined as number of 100 trial blocks before 0.8 fraction correct performance reached. Significance star indicates where time to criterion on an initial odour pair was greater or less than for the initial odour discrimination in AutonoMouse, or where trials to criterion on a novel odour discrimination task was significantly different to the AutonoMouse counterpart (1-way ANOVA, Tukey Kramer, F = 14.34, p = 2.9901x10^-20^).
(TIF)

S1 Appendix The appendix gives a technical overview of the AutonoMouse system and instructions intended to allow other users to replicate it. Included are photographs of the system; design schematics; a construction guide and software manual.
(DOCX)

## References

[1] Aronov D, Nevers R, Tank DW. Nature 2017;543:719–22. 10.1038/nature21692 28358077 PMC5492514

[2] Ben Arous J, Tanizawa Y, Rabinowitch I, Chatenay D, Schafer WR. J Neurosci Methods 2010;187:229–34. 10.1016/j.jneumeth.2010.01.011 20096306

[3] Tzschentke TM. Addict Biol 2007;12:227–462. 10.1111/j.1369-1600.2007.00070.x 17678505

[4] Vorhees C V., Williams MT. ILAR J 2014;55:310–32. 10.1093/ilar/ilu013 25225309 PMC4240437

[5] Claridge-Chang A, Roorda RD, Vrontou E, Sjulson L, Li H, Hirsh J, et al Cell 2009;139:405–15. 10.1016/j.cell.2009.08.034 19837039 PMC3920284

[6] Crawley JN. Neuron 2008;57:809–18. 10.1016/j.neuron.2008.03.001 18367082

[7] Harvey CD, Collman F, Dombeck DA, Tank DW. Nature 2009;461:941–6. 10.1038/nature08499 19829374 PMC2771429

[8] Maimon G, Straw AD, Dickinson MH. Nat Neurosci 2010;13:393–9. 10.1038/nn.2492 20154683

[9] O’Connor DH, Clack NG, Huber D, Komiyama T, Myers EW, Svoboda K. J Neurosci 2010;30:1947–67. 10.1523/JNEUROSCI.3762-09.2010 20130203 PMC6634009

[10] Rokni D, Hemmelder V, Kapoor V, Murthy VN. Nat Neurosci 2014;17:1225–32. 10.1038/nn.3775 25086608 PMC4146660

[11] Seelig JD, Chiappe ME, Lott GK, Dutta A, Osborne JE, Reiser MB, et al Nat Methods 2010;7:535–40. 10.1038/nmeth.1468 20526346 PMC2945246

[12] Silasi G, Boyd J, Bolanos F, LeDue J, Scott SH, Murphy TH. J Neurophysiol 2017:jn.00115.2017. 10.1152/jn.00115.2017 29070625

[13] Reuter JA, Spacek D V., Snyder MP. Mol Cell 2015;58:586–97. 10.1016/j.molcel.2015.05.004 26000844 PMC4494749

[14] Harris KD, Quiroga RQ, Freeman J, Smith SL. Nat Neurosci 2016;19:1165–74. 10.1038/nn.4365 27571195 PMC5244825

[15] Helmstaedter M. Nat Methods 2013;10:501–7. 10.1038/nmeth.2476 23722209

[16] Begemann I, Galic M. Front Synaptic Neurosci 2016;8:1–12. 10.3389/fnsyn.2016.00001 27601992 PMC4993758

[17] Pachitariu M, Steinmetz N, Kadir S, Carandini M, Harris KD. BioRxiv 2016:061481.

[18] Berning M, Boergens KM, Helmstaedter M. Neuron 2015;87:1193–206. 10.1016/j.neuron.2015.09.003 26402603

[19] Staffler B, Berning M, Boergens KM, Gour A, van der Smagt P, Helmstaedter M. Elife 2017;6:1–25. 10.7554/eLife.26414 PMC5658066 28708060

[20] Pnevmatikakis EA, Soudry D, Gao Y, Machado TA, Merel J, Pfau D, et al Neuron 2016;89:299. 10.1016/j.neuron.2015.11.037 PMC4881387 26774160

[21] Aoki R, Tsubota T, Goya Y, Benucci A. Nat Commun 2017;8 10.1038/s41467-017-00021-9 29084948 PMC5662625

[22] Maor I, Elyada Y, Mizrahi A. BioRxiv 2018.

[23] Gilestro GF, Cirelli C. Bioinformatics 2009;25:1466–7. 10.1093/bioinformatics/btp237 19369499 PMC2732309

[24] Machado AS, Darmohray DM, Fayad J, Marques HG, Carey MR. Elife 2015;4:1–22. 10.7554/eLife.07892 PMC4630674 26433022

[25] Rihel J, Prober DA, Arvanites A, Lam K, Zimmerman S, Jang S, et al Science (80-) 2010;327:348–51. 10.1126/science.1183090 20075256 PMC2830481

[26] Schaefer AT, Claridge-Chang A. Curr Opin Neurobiol 2012;22:170–6. 10.1016/j.conb.2011.11.004 22119142 PMC3398388

[27] Scott BB, Brody CD, Tank DW. Neuron 2013;80:371–84. 10.1016/j.neuron.2013.08.002 24055015 PMC4068252

[28] Wiltschko AB, Johnson MJ, Iurilli G, Peterson RE, Katon JM, Pashkovski SL, et al Neuron 2015;88:1121–35. 10.1016/j.neuron.2015.11.031 26687221 PMC4708087

[29] Skinner BF. The behavior of organisms: an experimental analysis. Oxford, England: Appleton-Century; 1938.

[30] Bodyak N, Slotnick B. Chem Senses 1999;24:637–45. 10587496 10.1093/chemse/24.6.637

[31] Rinberg D, Koulakov A, Gelperin A. J Neurosci 2006;26:8857–65. 10.1523/JNEUROSCI.0884-06.2006 16928875 PMC6674368

[32] Abraham NM, Spors H, Carleton A, Margrie TW, Kuner T, Schaefer AT. Neuron 2004;44:865–76. 10.1016/j.neuron.2004.11.017 15572116

[33] Uchida N, Mainen ZF. Nat Neurosci 2003;6:1224–9. 10.1038/nn1142 14566341

[34] Bussey TJ, Padain TL, Skillings EA, Winters BD, Morton AJ, Saksida LM. Learn Mem 2008;15:516–23. 10.1101/lm.987808 18612068 PMC2505319

[35] Stirman JN, Townsend LB, Smith SL. Vision Res 2016;127:74–83. 10.1016/j.visres.2016.07.006 27497283 PMC5035629

[36] Francis NA, Kanold PO. Front Neural Circuits 2017;11:1–6. 10.3389/fncir.2017.00001 28298887 PMC5331059

[37] Vinueza Veloz MF, Zhou K, Bosman LWJ, Potters JW, Negrello M, Seepers RM, et al Brain Struct Funct 2015;220:3513–36. 10.1007/s00429-014-0870-1 25139623 PMC4575700

[38] Balcombe JP, Barnard ND, Sandusky C. Contemp Top Lab Anim Sci 2004;43:42–51. 15669134

[39] Meijer MK, Sommer R, Spruijt BM, van Zutphen LFM, Baumans V. Lab Anim 2007;41:161–73. 10.1258/002367707780378168 17430616

[40] Sorge RE, Martin LJ, Isbester KA, Sotocinal SG, Rosen S, Tuttle AH, et al Nat Methods 2014;11:629–32. 10.1038/nmeth.2935 24776635

[41] Lu X, Slotnick BM. Neuroscience 1998;84:849–66. 9579789 10.1016/s0306-4522(97)00520-4

[42] Slotnick B. Chem Senses 2007;32:173–81. 17151135 10.1093/chemse/bjl046

[43] McBride K, Slotnick B. J Neurosci 2006;26:9892–901. 10.1523/JNEUROSCI.0504-06.2006 17005853 PMC6674478

[44] Knott TK, Madany PA, Faden AA, Xu M, Strotmann J, Henion TR, et al Neural Dev 2012;7:17 10.1186/1749-8104-7-17 22559903 PMC3390285

[45] Laurent G. Science (80-) 1999;286:723–8. 10531051 10.1126/science.286.5440.723

[46] Lledo P, Gheusi G, Vincent J. Physiol Rev 2005;85:281–317. 10.1152/physrev.00008.2004 15618482

[47] Wilson RI, Mainen ZF. Annu Rev Neurosci 2006;29:163–201. 10.1146/annurev.neuro.29.051605.112950 16776583

[48] Zhang Y, Sharpee TO. PLoS Comput Biol 2016;12:1–15. 10.1371/journal.pcbi.1004850 PMC4827830 27065441

[49] Johnson BA, Leon M. J Comp Neurol 2007;503:1–34. 10.1002/cne.21396 17480025 PMC2213457

[50] Bracey EF, Pichler B, Schaefer AT, Wallace DJ, Margrie TW. Nat Commun 2013;4:2100 10.1038/ncomms3100 23820818 PMC3715885

[51] Voikar V, Colacicco G, Gruber O, Vannoni E, Lipp H, Wolfer DP. Behav Brain Res 2010;213:304–12. 10.1016/j.bbr.2010.05.019 20493907

[52] Winter Y, Schaefers ATU. J Neurosci Methods 2011;196:276–80. 10.1016/j.jneumeth.2011.01.017 21256865

[53] Weissbrod A, Shapiro A, Vasserman G, Edry L, Dayan M, Yitzhaky A, et al Nat Commun 2013;4:1–10. 10.1038/ncomms3018 23771126

[54] Bains RS, Cater HL, Sillito RR, Chartsias A, Sneddon D, Concas D, et al Front Behav Neurosci 2016;10:1–12. 10.3389/fnbeh.2016.00001 27375446 PMC4901040

[55] Van Loo PLP, Mol JA, Koolhaas JM, Van Zutphen BFM, Baumans V. Physiol Behav 2001;72:675–83. 11336999 10.1016/s0031-9384(01)00425-5

[56] Van Loo PLP, Van Zutphen LFM, Baumans V. Lab Anim 2003;37:300–13. 10.1258/002367703322389870 14599305

[57] Gouveia K, Hurst JL. Sci Rep 2017;7:1–12. 10.1038/s41598-016-0028-x 28322308 PMC5359560

[58] Poddar R, Kawai R, Ölveczky BP. PLoS One 2013;8:1–10. 10.1371/journal.pone.0083171 PMC3857823 24349451

[59] Abraham NM, Spors H, Carleton A, Margrie TW, Kuner T, Schaefer AT. Neuron 2004;44:865–76. 10.1016/j.neuron.2004.11.017 15572116

[60] Shimshek DR, Bus T, Kim J, Mihaljevic A, Mack V, Seeburg PH, et al PLoS Biol 2005;3:e354 10.1371/journal.pbio.0030354 16216087 PMC1255741

[61] Abraham NM, Egger V, Shimshek DR, Renden R, Fukunaga I, Sprengel R, et al Neuron 2010;65:399–411. 10.1016/j.neuron.2010.01.009 20159452 PMC6366558

[62] Lepousez G, Lledo P-M. Neuron 2013:1–15. 10.1016/j.neuron.2013.07.025 24139818

[63] Doty RL, Brugger WE, Jurs PC, Orndorff MA, Snyder PJ, Lowry LD. Physiol Behav 1978;20:175–85. 662939 10.1016/0031-9384(78)90070-7

[64] Cometto-Muñiz JE, Cain WS, Abraham MH. Chem Senses 2005;30:627–42. 10.1093/chemse/bji056 16141291

[65] Chen V, Halpern BP. Chem Senses 2008;33:107–18. 10.1093/chemse/bjm069 17962230

[66] Uchida N, Mainen ZF. Front Syst Neurosci 2007;1:3 10.3389/neuro.06.003.2007 18958244 PMC2526272

[67] Resulaj A, Rinberg D. J Neurosci 2015;35:11667–73. 10.1523/JNEUROSCI.4693-14.2015 26290243 PMC4540801

[68] Abraham NM, Guerin D, Bhaukaurally K, Carleton A. PLoS One 2012;7:e51789 10.1371/journal.pone.0051789 23272168 PMC3525655

[69] Murphy TH, Boyd JD, Bolaños F, Vanni MP, Silasi G, Haupt D, et al Nat Commun 2016;7. 10.1038/ncomms11611 PMC4909937 27291514

[70] Wentz CT, Bernstein JG, Monahan P, Guerra A, Rodriguez A, Boyden ES. J Neural Eng 2011;8:46021. 10.1088/1741-2560/8/4/046021 PMC3151576 21701058

[71] Park SI, Brenner DS, Shin G, Morgan CD, Copits BA, Chung HU, et al Nat Biotech 2015;33:1280–6. 10.1038/nbt.3415 PMC4880021 26551059

[72] Szuts TA, Fadeyev V, Kachiguine S, Sher A, Grivich M V, Agrochao M, et al Nat Neurosci 2011;14:263–9. 10.1038/nn.2730 21240274

[73] Hasegawa T, Fujimoto H, Tashiro K, Nonomura M, Tsuchiya A, Watanabe D. Sci Rep 2015;5:7853 10.1038/srep07853 25597933 PMC4297970

[74] Lu L, Gutruf P, Xia L, Bhatti DL, Wang X, Vazquez-Guardado A, et al Proc Natl Acad Sci 2018;115:E1374–83. 10.1073/pnas.1718721115 29378934 PMC5816195

[75] Bechara A, Laumonnerie C, Vilain N, Kratochwil CF, Cankovic V, Maiorano NA, et al Cell Rep 2015;13:783–97. 10.1016/j.celrep.2015.09.031 26489473

[76] Schaefer ML, Böttger B, Silver WL, Finger TE. J Comp Neurol 2002;444:221–6. 10.1002/cne.10143 11840476

[77] Dewan A, Cichy A, Zhang J, Miguel K, Feinstein P, Rinberg D, et al Nat Commun 2018;9:1–12. 10.1038/s41467-017-02088-w 30038239 PMC6056506

[78] Saito S, Murase K. Br J Radiol 2012;85:e973–8. 10.1259/bjr/13040401 22674712 PMC3500820

