# Supplementary figures and images for: AutonoMouse: High throughput operant conditioning reveals progressive impairment with graded olfactory bulb lesions
Source: PLoS One. 2019 Mar 6;14(3):e0211571. doi: 10.1371/journal.pone.0211571 (PMC6402634; doi:10.1371/journal.pone.0211571)

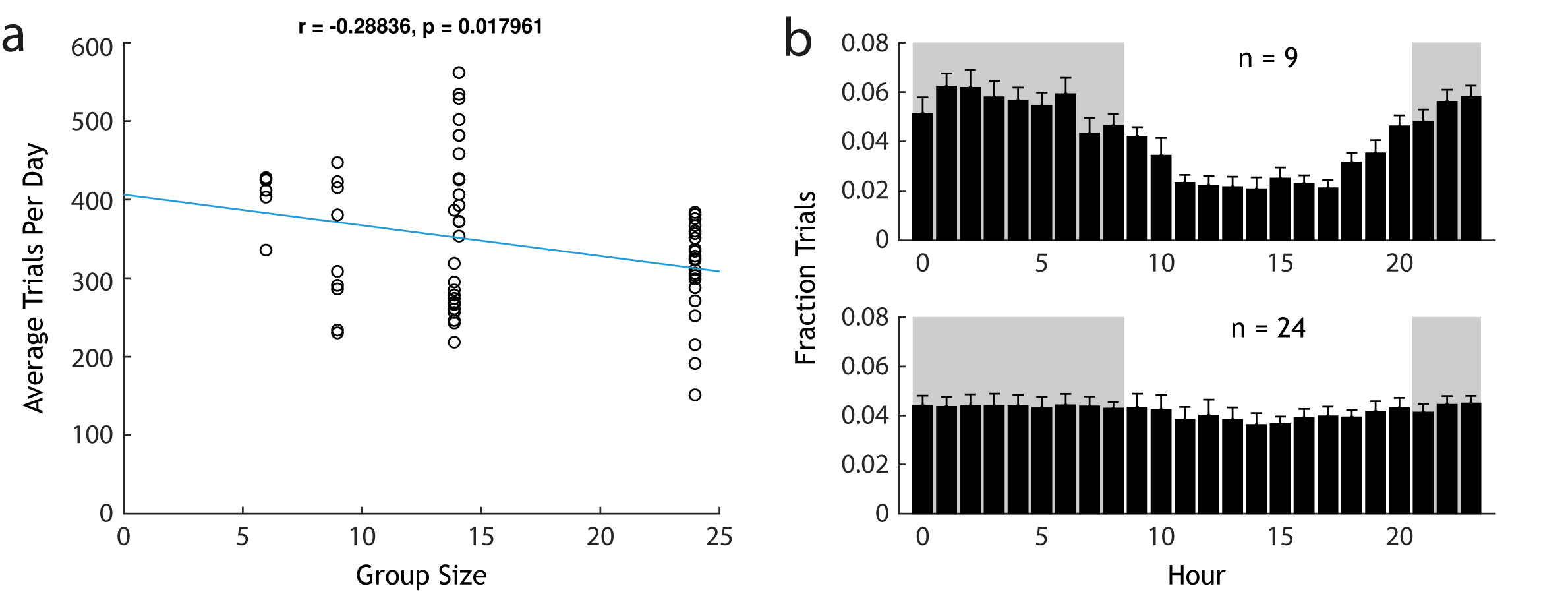

Supplement: S1 Fig — (a) Average trials per day for each animal plotted against the group size (number of animals) in which the animal performed. There is a significant negative correlation between group size and daily trials performed for each animal. (b) Fraction of trials performed each hour analysed as in Fig 2D for a cohort of n = 9 mice (top) and n = 24 mice (bottom). (TIF) [file pone.0211571.s001.tif]

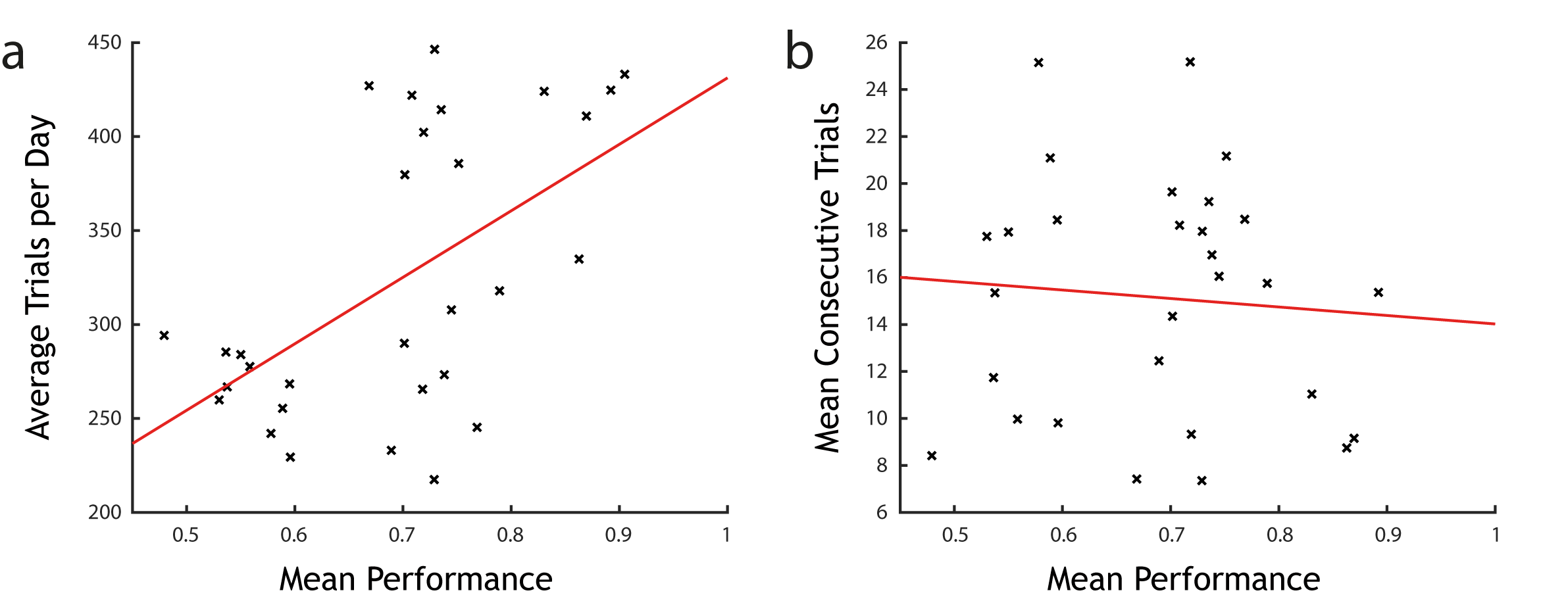

Supplement: S2 Fig — Performance is correlated with daily trials performed, but not with average stretch length of those trials (a) Mean performance of all lesion study animals (n = 29) against average daily trials performed. Average daily trials is significantly positively correlated with mean performance (R = 0.5329, p = 0.0029). (b) Mean performance of the same animals against mean of length of consecutive trials performed. There is no significant correlation between mean performance and mean consecutive trials (R = -0.0787, p = 0.6847). (TIF) [file pone.0211571.s002.tif]

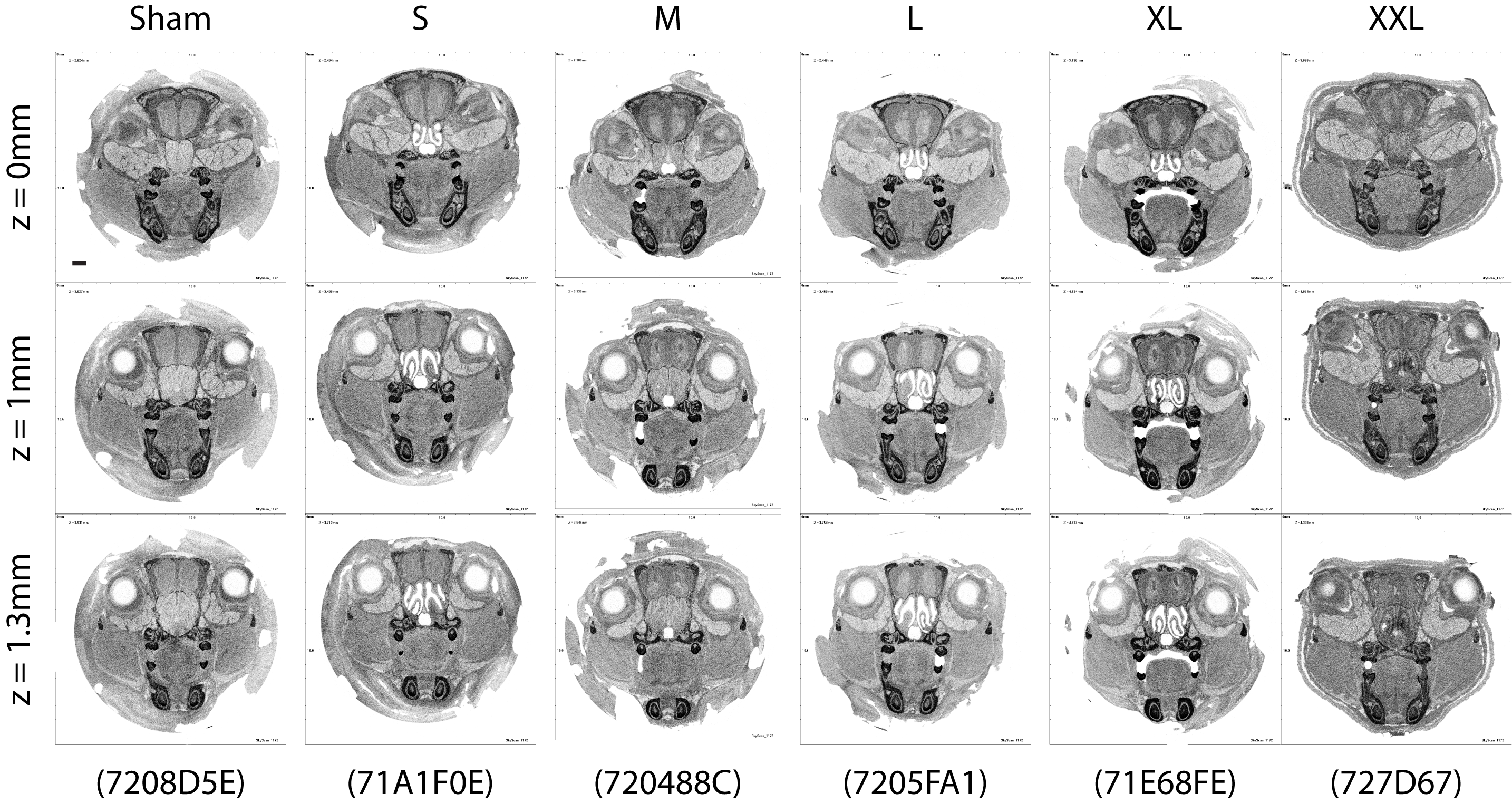

Supplement: S3 Fig — MicroCT images from mice injected with varying amounts of NMDA into the olfactory bulb (Sham: 0ng, S: 303.6ng, M: 607.2ng, L: 1214ng, XL 1669.8ng, XXL: 2125ng). Images are reconstructed coronal sections from a whole mouse head, starting at 0mm from bregma, to 1–1.3mm anterior from bregma (roughly the olfactory bulb injection site). Images are inverted such that darker regions correspond to more x-ray absorbent areas (e.g. skull, teeth, soft tissue absent areas where contrast agent has pooled). Bottom row: codes in brackets indicate RFID of animal used as representative example. (TIF) [file pone.0211571.s003.tif]

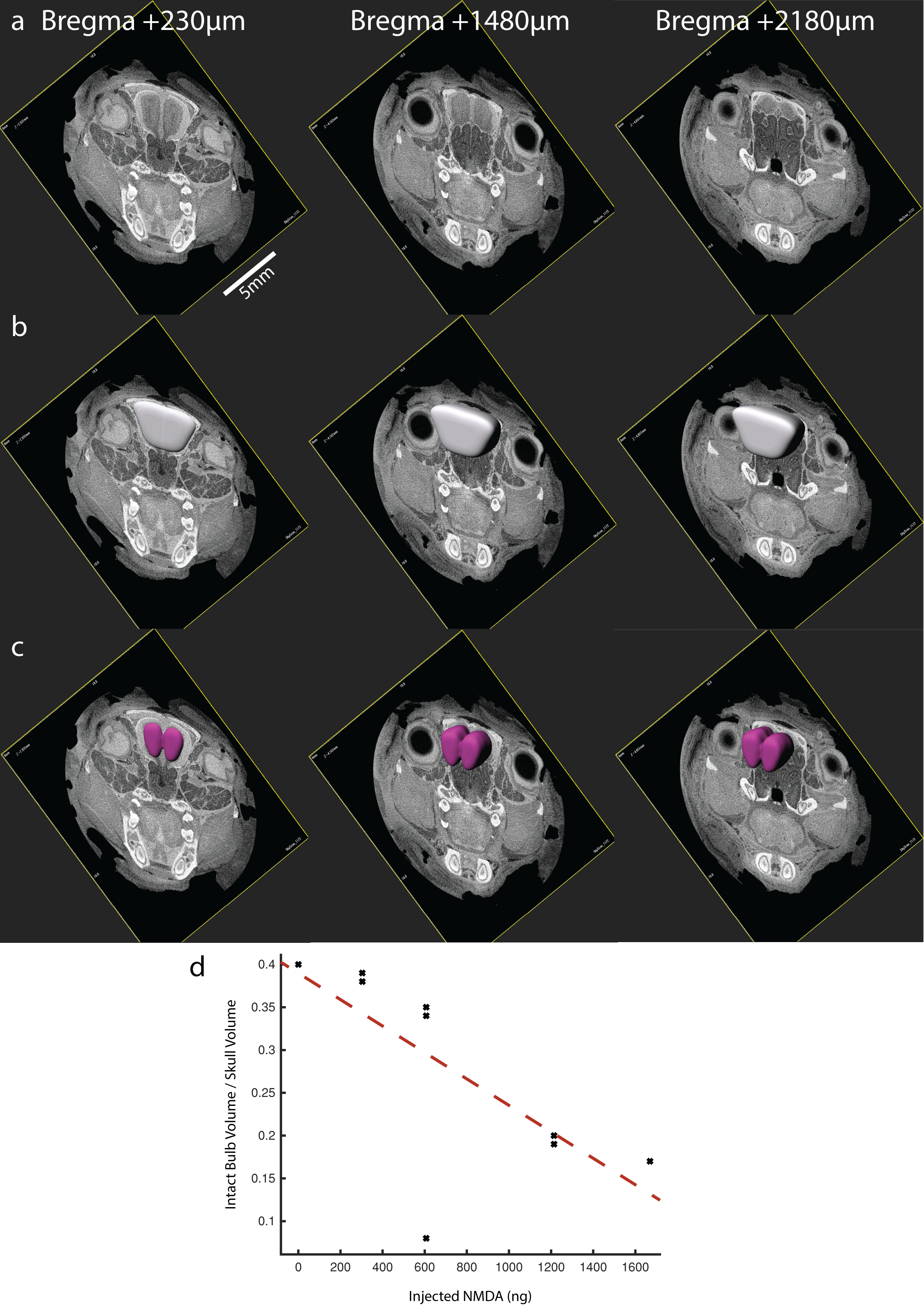

Supplement: S4 Fig — (a) Row of images shows example reconstructed coronal CT slices in rostral to caudal direction (left to right) in the olfactory bulb region caudal to bregma. (b) Same example images as in (a) after skull volume has been manually defined by tracing the outline in the stack of slices. Grey volume shows reconstructed skull volume around the bulb. (c) Same images as in (a) after bulbar volume has been defined manually. Pink volume is reconstructed bulbar volume. (d) After manually defining intact bulb volume and skull volume, bulb volume relative to skull volume is plotted against injected NMDA for 9 CT imaged mice. There is a significant (and with one exception very tight) negative correlation between relative bulbar volume and injected NMDA (Pearson correlation coefficient R = -0.7005, p = 0.0356), showing that larger volumes of injected NMDA have a graded, progressive effect on olfactory bulb damage. (TIF) [file pone.0211571.s004.tif]

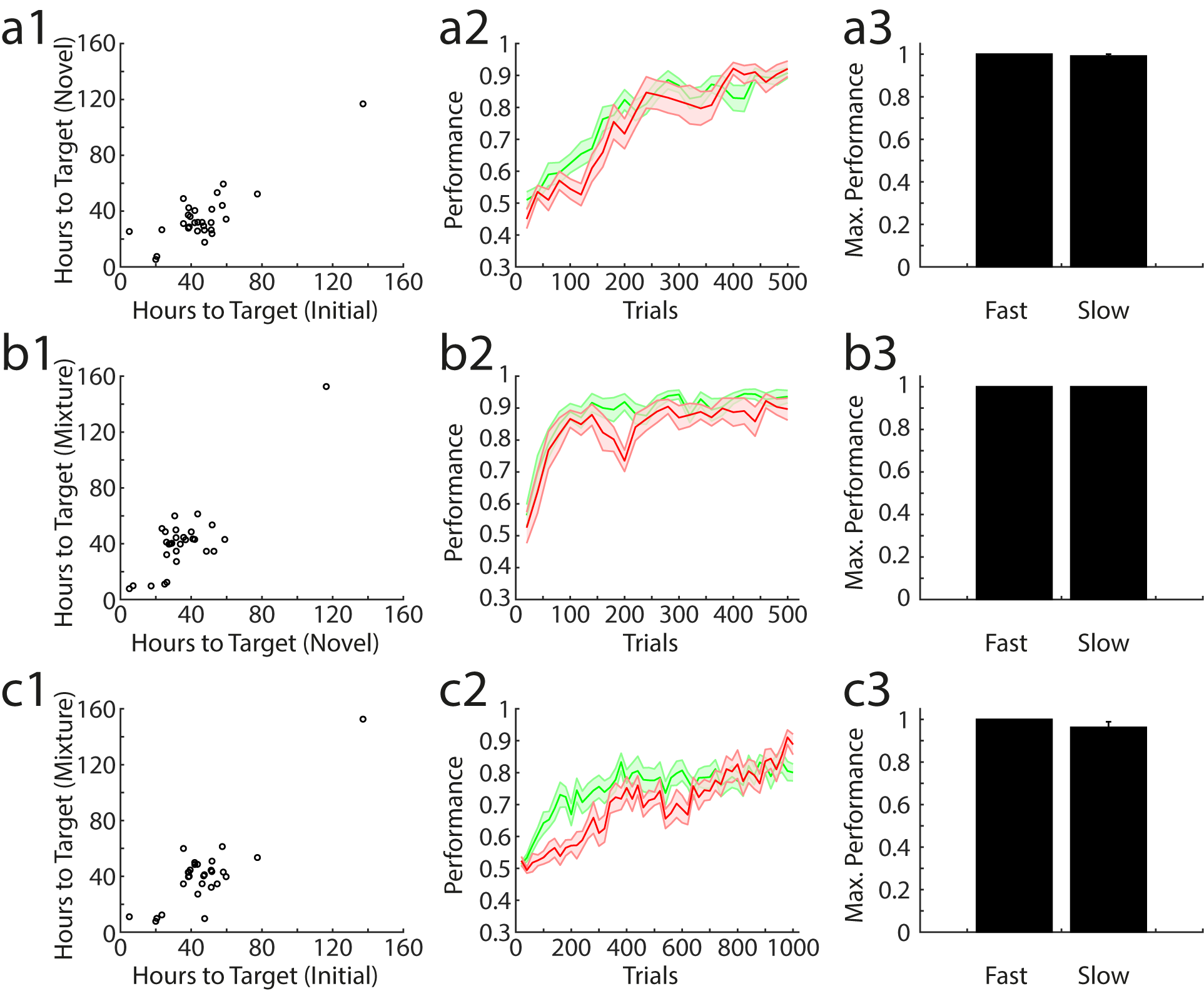

Supplement: S5 Fig — (a1) Number of hours taken to perform a target number of trials (1st 500) during initial odour pair learning vs. novel odour pair learning (n = 29). Hours to target are significantly correlated across the two task types (R = 0.84, p = 1.22x10^-8^). (a2) Performing animals are classified according to the rate at which they perform trials. For 4 task types (initial, novel, mixture, familiar) the time taken to perform the 1st 500 trials in each was averaged for each animal. Fast (green, n = 17) animals are those with mean time to target completion greater than the mean time to completion over all animals and slow (red, n = 12) animals are those with mean time to target completion less than this average. Performance is shown for both groups on initial odour pair discrimination. (a3) Mean maximum performance in the initial odour pair discrimination for the same groups in (a2). (b1) Hours to target for novel odour pair vs. mixture learning (R = 0.85, p = 5.48x10^-9^). (b2) Performance for the fast and slow groups in a novel odour pair task. (b3) Average maximum performance in the novel odour pair task. (c1) Hours to target for initial vs. mixture learning (R = 0.86, p = 1.54x10^-9^). (c2) Performance for the fast and slow groups in a mixture discrimination task. (c3) Average maximum performance in the mixture discrimination task. (TIF) [file pone.0211571.s005.tif]

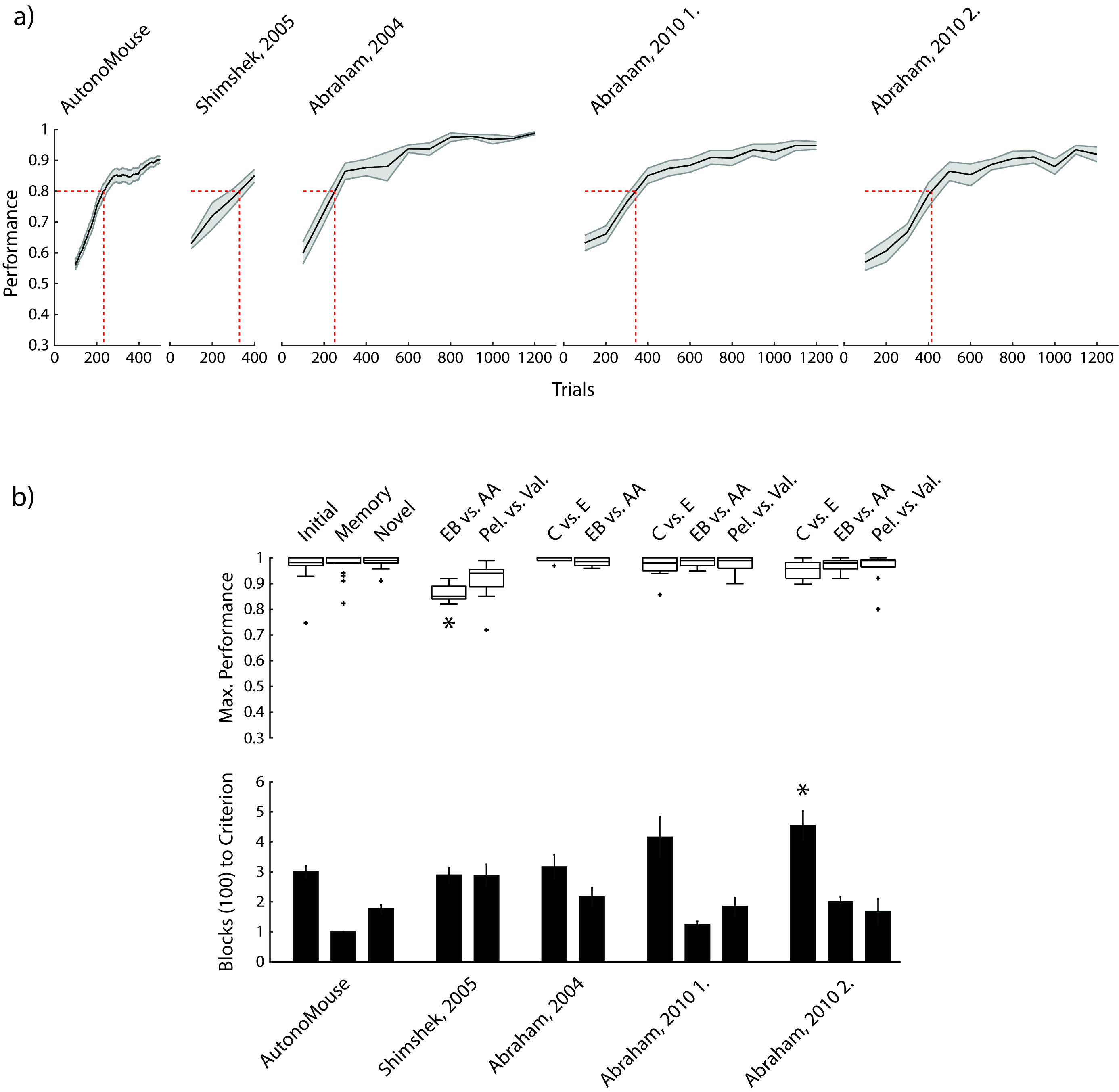

Supplement: S6 Fig — a) Learning progression for different experimental groups from different previous studies on the first odour pair discrimination introduced (initial). Performance calculated in bins of 100 trials. AutonoMouse n = 29; Shimshek, 2005 n = 9; Abraham, 2004 n = 6; Abraham, 2010 group 1 n = 9; Abraham, 2010 group 2 n = 13. b) Top: Boxplots of maximum performance obtained in several odour discrimination tasks for the same studies and animals shown in a). Significance star indicates cases where maximum performance was significantly lower than all other performance cases (1-way ANOVA, Tukey Kramer, F = 8.1, p = 6.2x10^-12^). Bottom: Bar plots (mean +/- sem) of trials to criterion–defined as number of 100 trial blocks before 0.8 fraction correct performance reached. Significance star indicates where time to criterion on an initial odour pair was greater or less than for the initial odour discrimination in AutonoMouse, or where trials to criterion on a novel odour discrimination task was significantly different to the AutonoMouse counterpart (1-way ANOVA, Tukey Kramer, F = 14.34, p = 2.9901x10^-20^). (TIF) [file pone.0211571.s006.tif]
