# Supplementary material for: AutonoMouse: High throughput operant conditioning reveals progressive impairment with graded olfactory bulb lesions
Source: PLoS One. 2019 Mar 6;14(3):e0211571. doi: 10.1371/journal.pone.0211571 (PMC6402634; doi:10.1371/journal.pone.0211571)
Supplement: S1 Appendix — Included are photographs of the system; design schematics; a construction guide and software manual. (DOCX) [file pone.0211571.s007.docx]

S1 Appendix

In this appendix we describe all mechanical (Appendix Fig A-J) and electronic (Appendix Fig K-M) parts. A construction manual for assembling the system is also provided, as well as a software manual with guidance on setting up and running behavioural experiments with AutonoMouse.

# Contents

[Cage design 2](#_Toc510602704)

[Electronic schematics 11](#_Toc510602705)

[Construction manual 14](#_Toc510602706)

[General notes 14](#_Toc510602707)

[Main frame construction 14](#_Toc510602708)

[Cleaning chamber construction 18](#_Toc510602709)

[Home cage construction 19](#_Toc510602710)

[Pre-chamber construction 21](#_Toc510602711)

[Door mechanism construction 21](#_Toc510602712)

[Access tunnel and behaviour port construction 22](#_Toc510602713)

[Olfactometer 23](#_Toc510602714)

[Wiring / sensor connections 25](#_Toc510602715)

[Software manual 26](#_Toc510602716)

[Introduction 26](#_Toc510602717)

[schedule-generator 26](#_Toc510602718)

[Installation 26](#_Toc510602719)

[Running the schedule generator 26](#_Toc510602720)

[The main window 27](#_Toc510602721)

[Generating a schedule 28](#_Toc510602722)

[Schedule generation example 28](#_Toc510602723)

[autonomouse-control 33](#_Toc510602724)

[Installation 33](#_Toc510602725)

[Running autonomouse-control 33](#_Toc510602726)

[The main window 33](#_Toc510602727)

[Hardware control 33](#_Toc510602728)

[Animal management 34](#_Toc510602729)

[Analysis 35](#_Toc510602730)

[Example experiment 36](#_Toc510602731)

[Exporting data 43](#_Toc510602732)

[Advanced schedule-generator usage 45](#_Toc510602733)

[Schedule widget creation example 45](#_Toc510602734)

[Pulse parameters 54](#_Toc510602735)

# Cage design


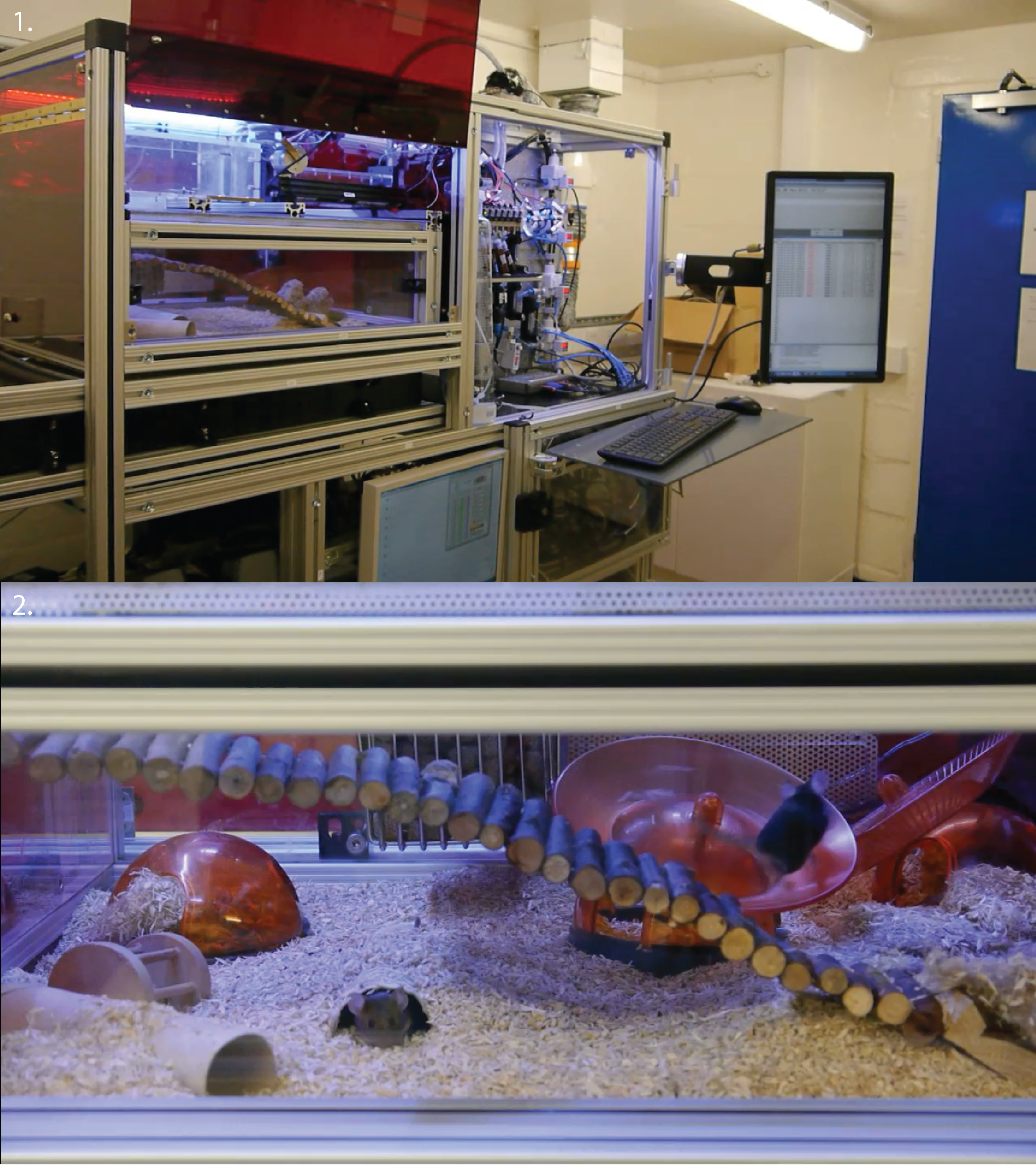


Figure A – 1. View of the main system with full frame and home cage. 2. View of the inside of home cage during an experiment.


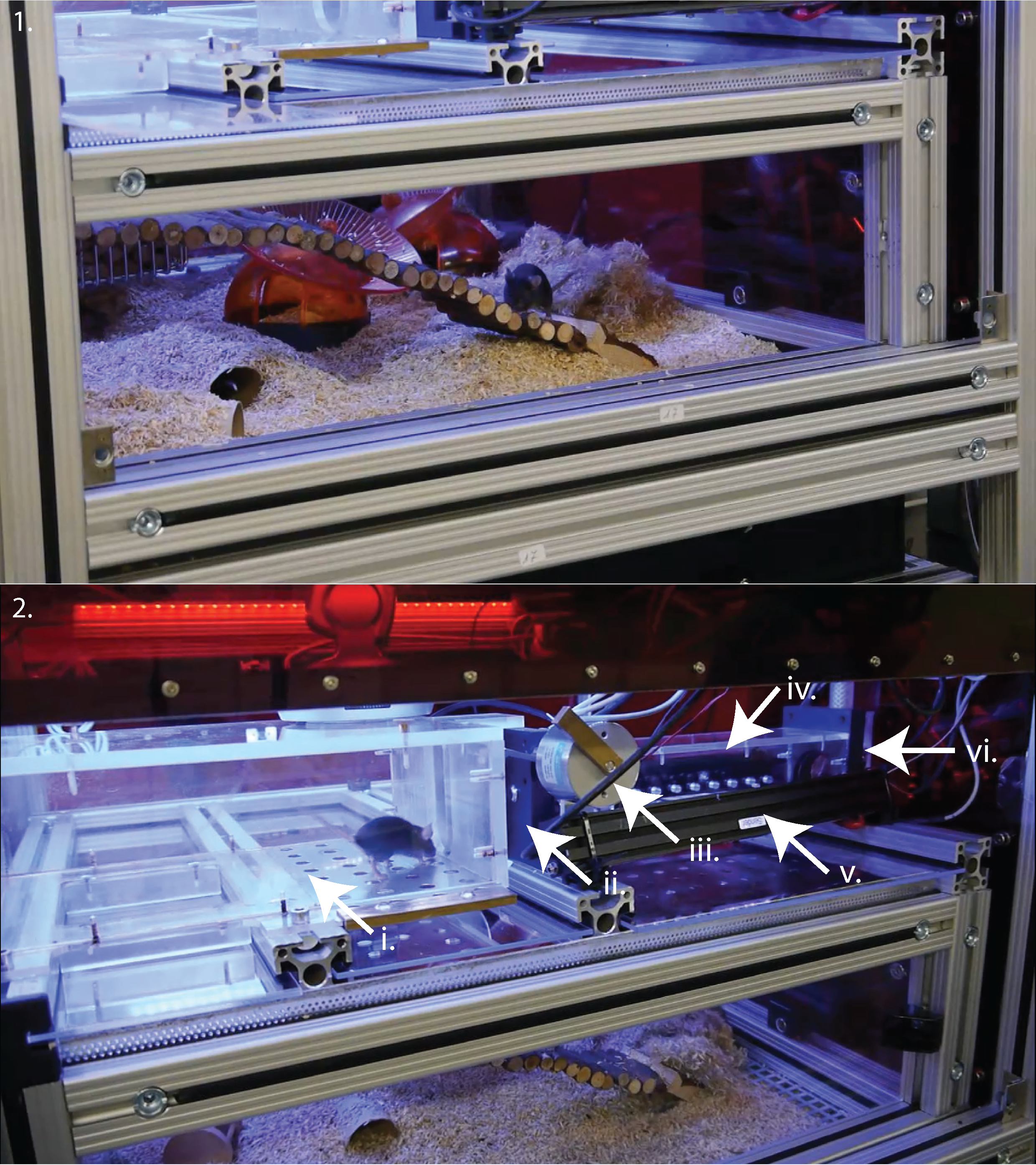


Figure B – 1. View of the home cage with mouse ascending to behavioural area. 2. View of behavioural area. **(i)** Pre-chamber. **(ii)** Door holder apparatus. **(iii)** Rotary motor controlling door in open position. **(iv)** Access tunnel. **(v)** IR beam sensors. **(vi)** Behaviour port.


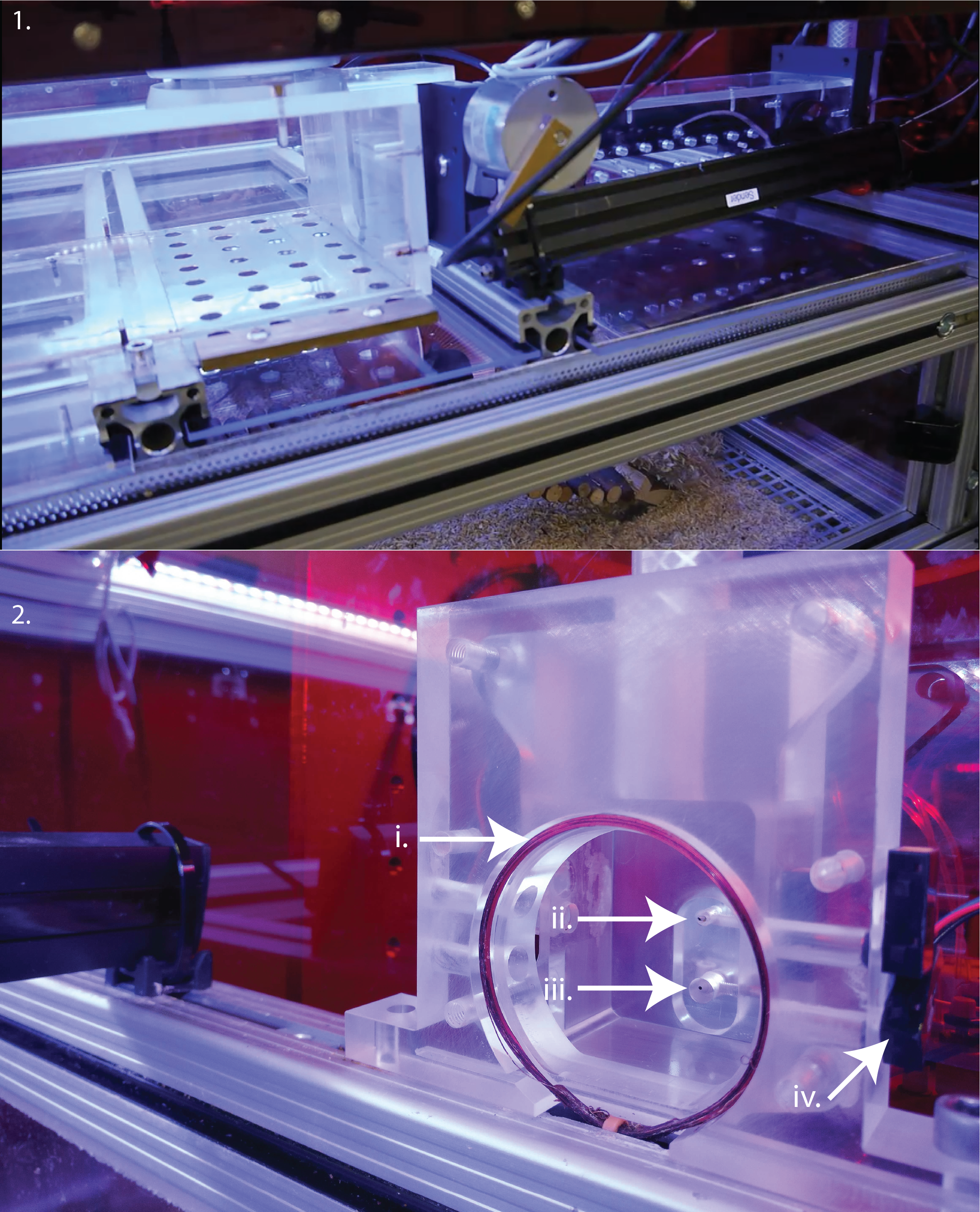


Figure C – 1. Closer view of the behavioural area after mouse has entered the access tunnel. The door is in the closed position. 2. View of the behaviour port, corresponding to CAD drawing shown in Figure H. **(i)** RFID coil and housing. **(ii)** Water delivery port and lick detector. **(iii)** Odour delivery port. **(iv)** IR beam sensors.


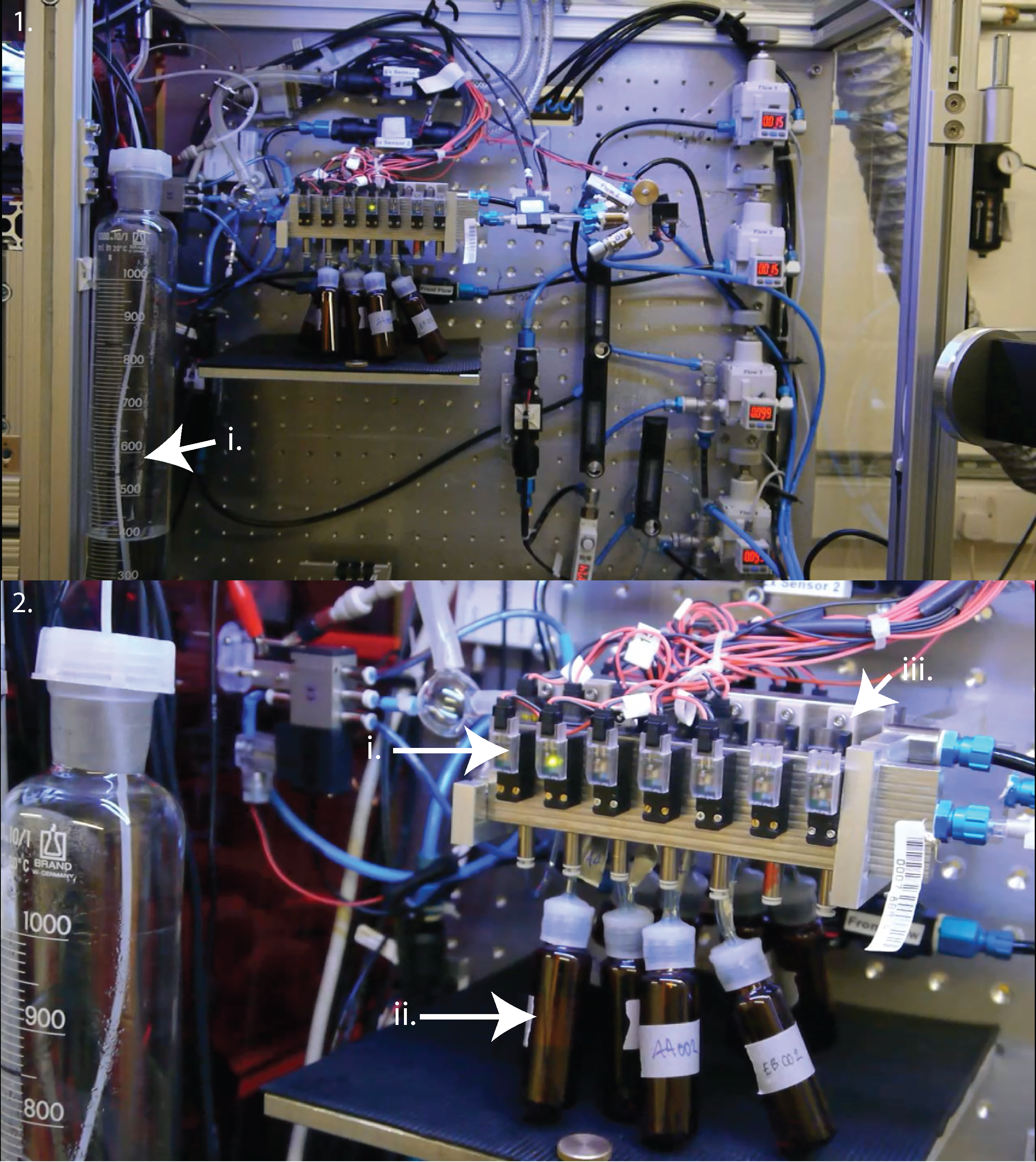


Figure D – 1. View of the stimulus generation area housing the olfactometer. **(i)** Water reservoir. 2. Closer view of the olfactometer. **(i)** Input valves. **(ii)** Odour bottles. **(iii)** Main block valves.


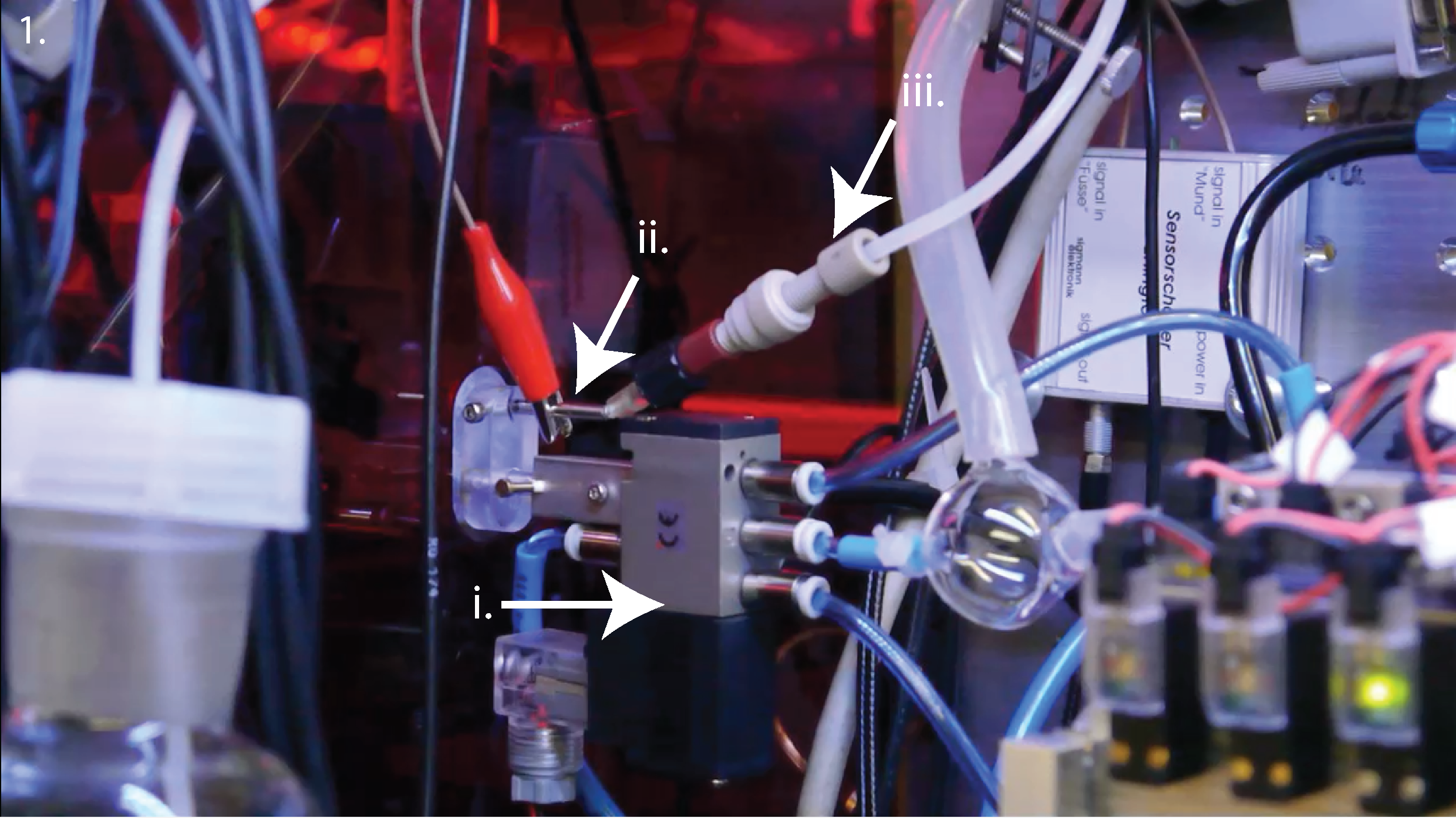


Figure E – 1. View of the olfactometer output into the behaviour port. **(i)** Final valve for delivering odour stimulus. **(ii)** Lick port / sensor. **(iii)** Connector between lick port and water reservoir.


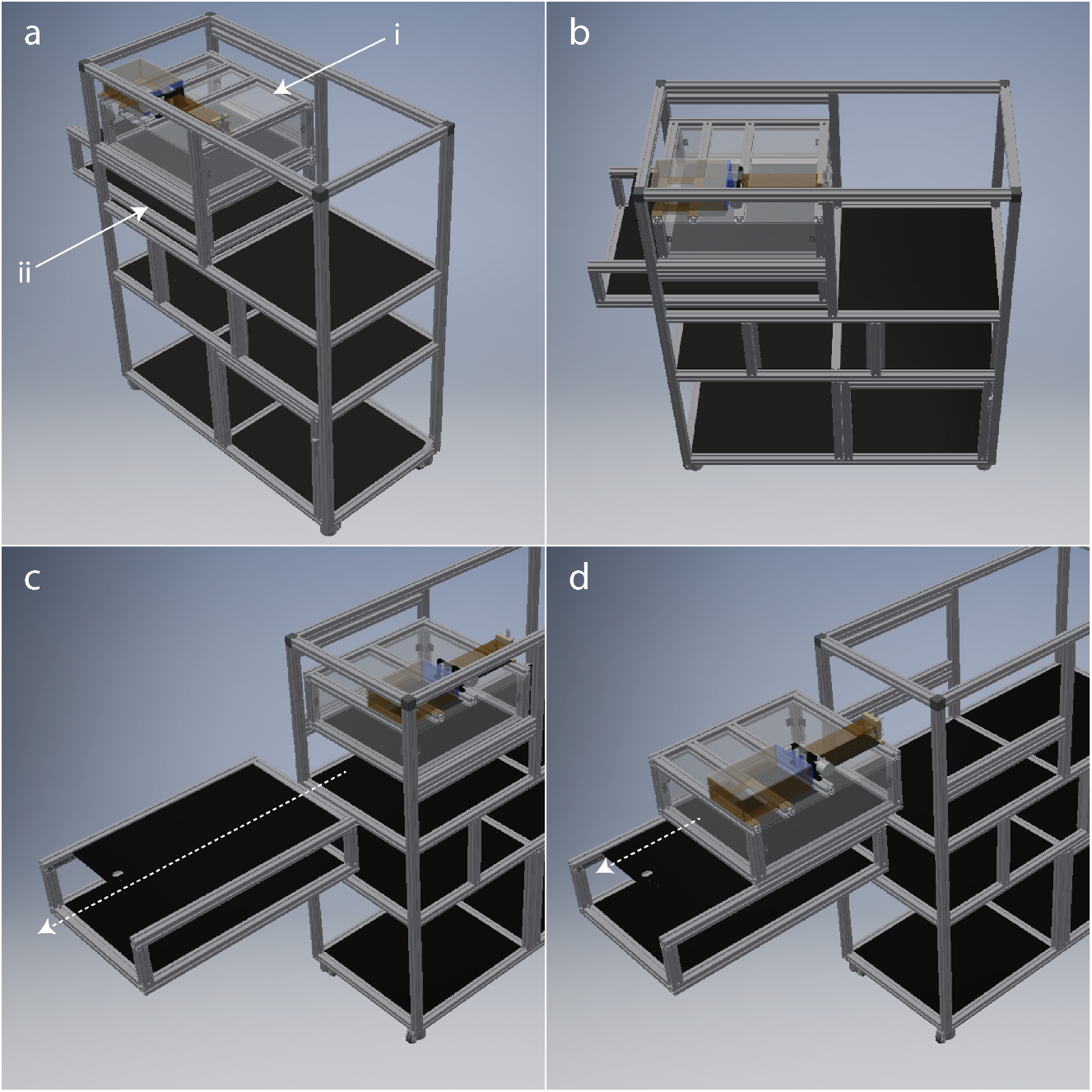


Figure F – Full system. **(a)** Top/front/side view of system. **(i)** Home cage system housing mice and installed with pre-chamber, access door and behaviour port. **(ii)** Lower bedding capture chamber. **(b)** Top/front view. **(c)** Illustration of removal of bedding chamber. **(d)** Removal of home cage chamber


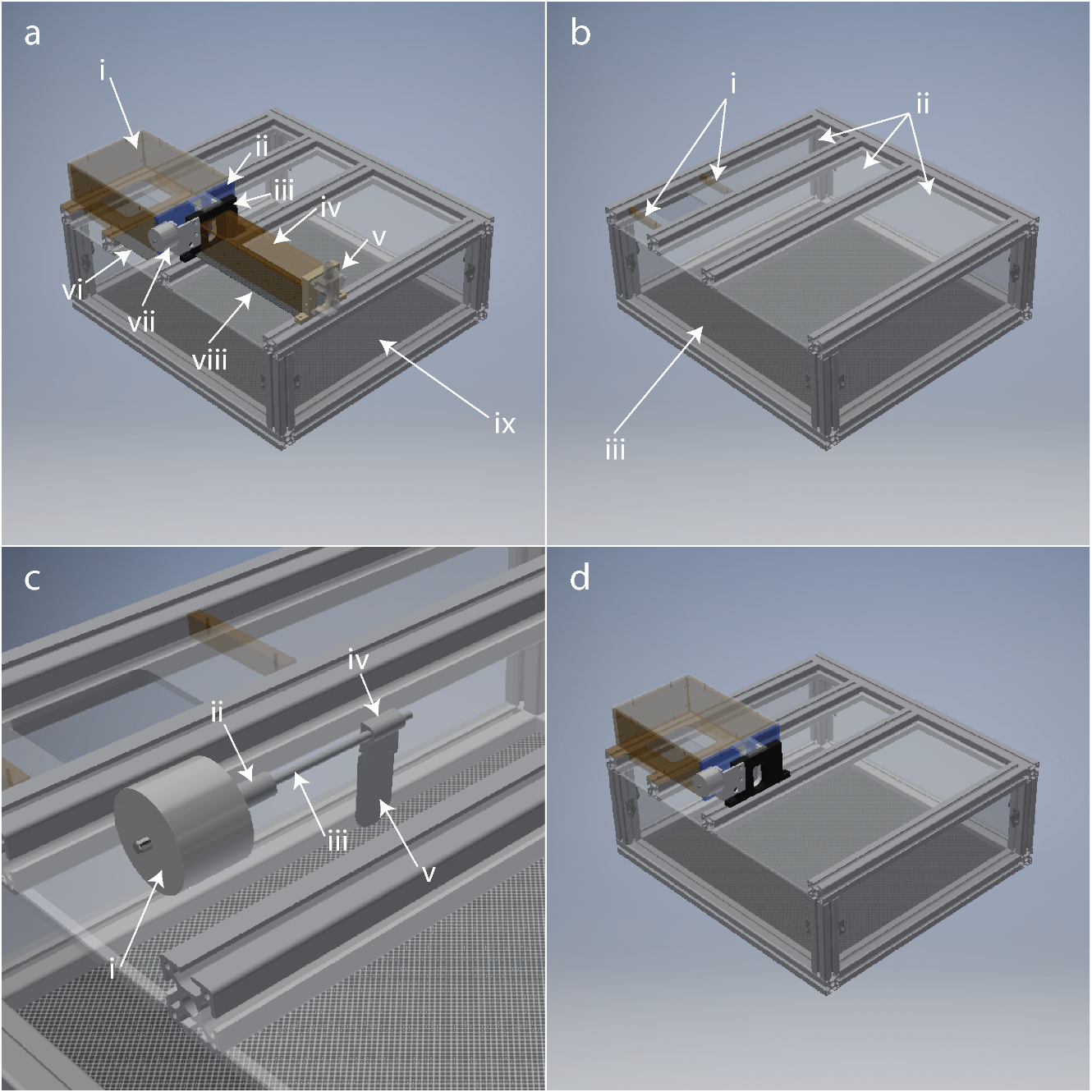


Figure G – Home cage. **(a)** View of complete home cage construction. **(i)** Pre-chamber linking lower home cage to upper behavioural area. **(ii)** Linker between pre-chamber and access tunnel to behaviour port. **(iii)** Assembly for housing access door. **(iv)** Access tunnel linking pre-chamber and behaviour port. **(v)** Behaviour port. **(vi)** Pre-chamber floor grating for removing waste. **(vii)** Assembly housing rotary magnet and door shaft for controlling access door. **(viii)** Access tunnel floor grating for removing waste. **(ix)** Home cage floor grating, allows for excess bedding to be removed. **(b)** View of access panels for home cage / behavioural area. **(i)** Spacers for attachment of pre-chamber. **(ii)** Removable access panels for top access between home cage and behavioural area. **(iii)** Front removable panel for mouse access. **(c)** Internal view of door mechanism. **(i)** Rotary magnet for changing door position. **(ii)** Adapter coupling between rotary magnet and door / door axel. **(iii)** Door axel. **(iv)** Adapter for connecting door panel to axel. **(v)** Door panel. **(d)** View of pre-chamber and door frame without occluding access tunnel.


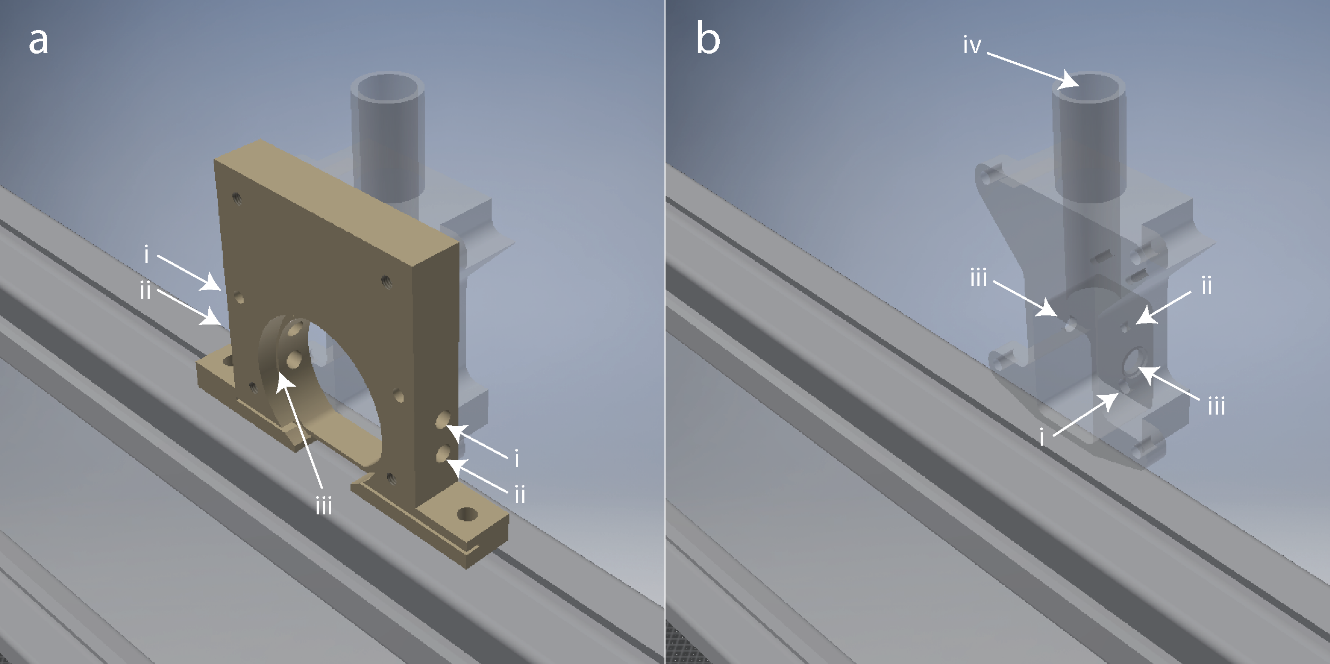


Figure H – Behaviour port. **(a)** View of complete behaviour port. **(i)** IR beam detector pair housing. **(ii)** Housing for 2^nd^ IR beam detector pair. **(iii)** Housing for RFID detection coil. **(b)** Reduced view of behaviour port showing just the main sensor housing. **(i)** Stimulus port housing for an odour tube. **(ii)** Lick spout housing. **(iii)** Housing for the main IR beam detector pair. **(iv)** Exhaust / vent for the behaviour port.


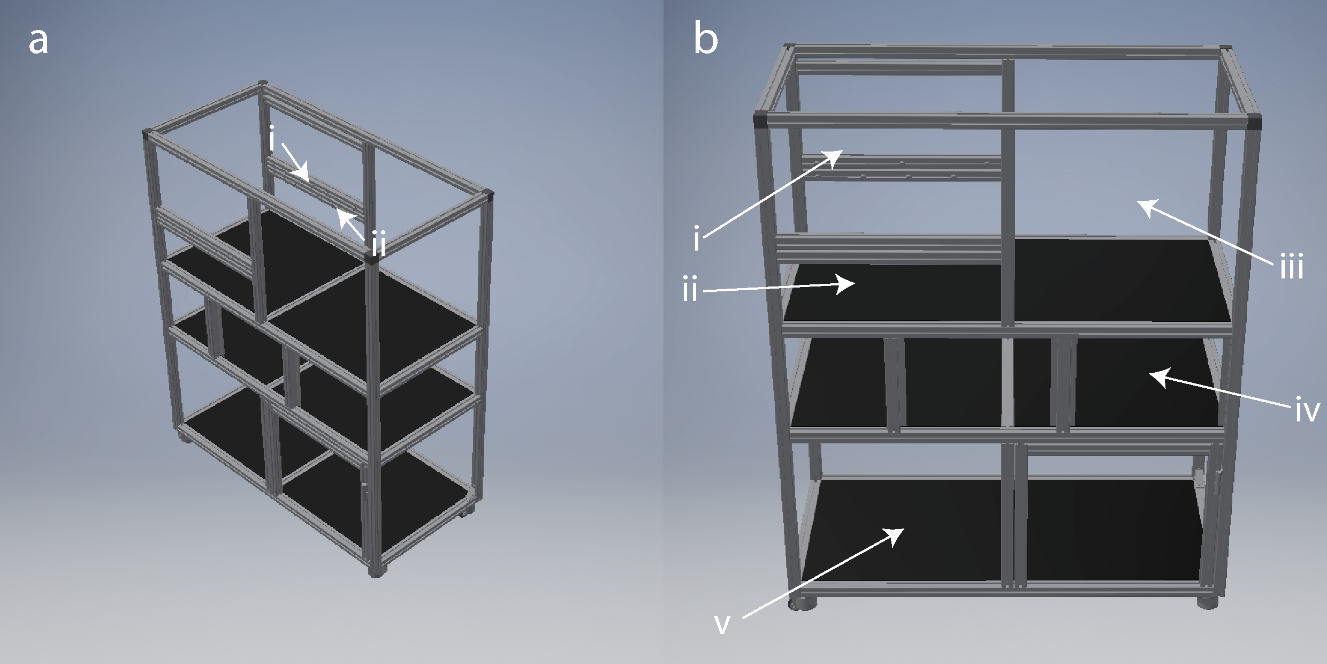


Figure I – System frame. **(a)** View of complete system frame without home cage / bedding chamber. **(i)** Guide rail for home cage insertion. **(ii)** Guide rail for bedding chamber insertion. **(b)** Front view of system frame. **(i)** Home cage area. **(ii)** Bedding chamber area. **(iii)** Area for installation of stimulus production, e.g. olfactometer, monitors. **(iv)** Middle chamber for housing electronics, power supplies, DAQ boards etc. **(v)** Lower chamber for housing other hardware, e.g. PCs.


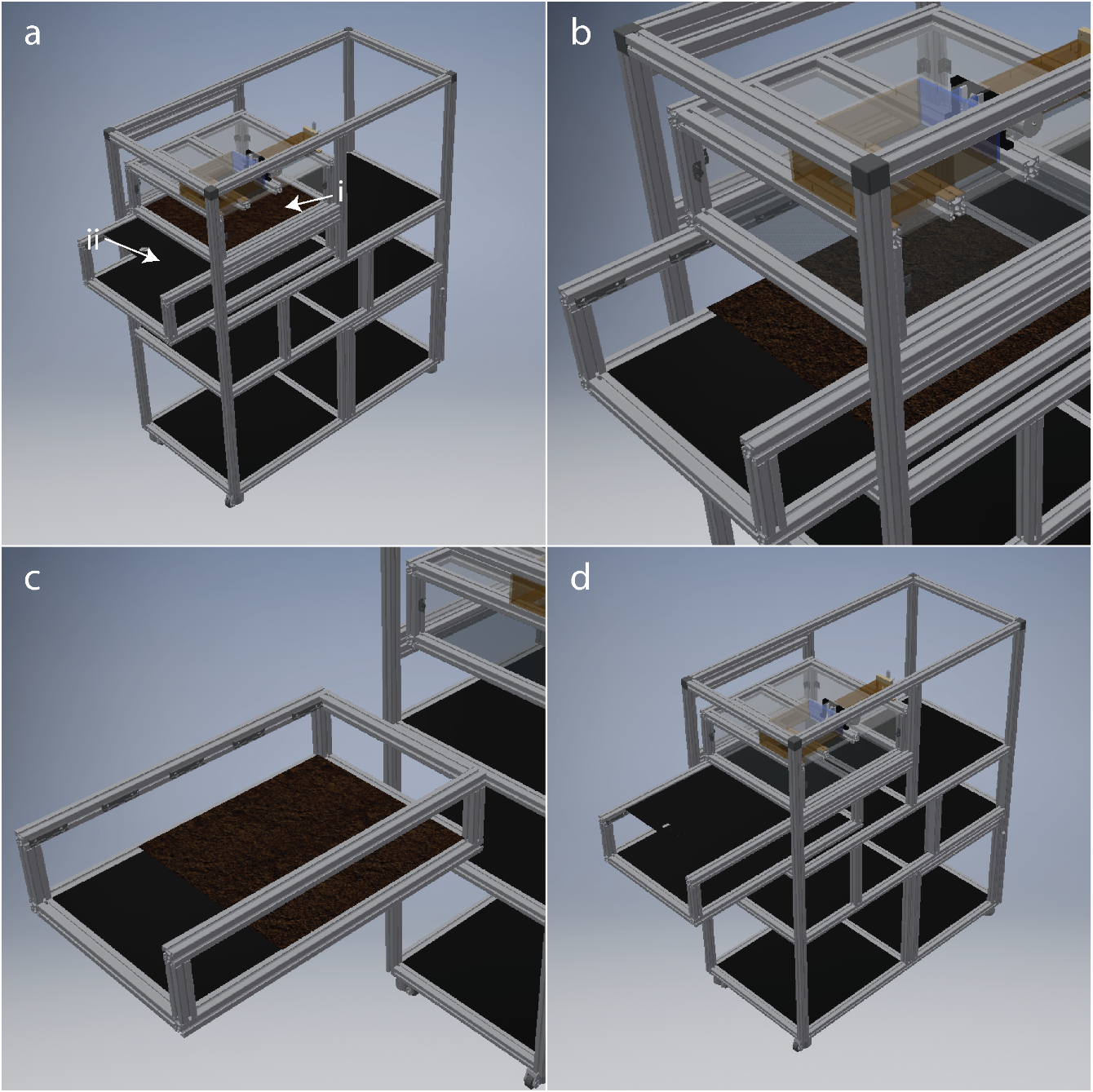


Figure J – Cage cleaning. **(a)** View of AutonoMouse system with **(i)** cage bedding in the home cage. **(ii)** The cleaning plate holds the bedding in place above the bedding removal chamber. **(b)** During an experiment, the bedding can be removed by sliding out the cleaning plate and brushing remaining bedding through the home cage floor mesh into the bedding removal chamber. **(c)** The entire bedding chamber can then be removed in order to dispose of the bedding easily. **(d)** The chamber and cleaning plate are reinstalled to allow fresh bedding to be added to the home cage. The home cage does not need to be moved during the process.

# Electronic schematics

Figure K – Lick detector circuit schematic. See lick-detector.sch in the ElectronicsModules section of the autonomouse-design repository

Figure L – IR beam logic circuit schematic. See ir-logic.sch in the ElectronicsModules section of the autonomouse-design repository

Figure M – Access door control circuit schematic. See door-close.sch in the ElectronicsModules section of the autonomouse-design repository

# Construction manual

## General notes

The frame for the entire AutonoMouse system is built primarily from MAYTEC aluminium profiles. Connection elements between profiles are not shown in CAD drawings or construction diagrams but are uniform throughout the construction. All profiles should be obtained with cross bushing bores at both ends of the profile, equal distances from each end to facilitate housing of connectors. For connections between profiles, the MAYTEC universal-connector (cross bushing and round-headed anchor) can be used throughout. For attachments of accessories to profile faces, standard MAYTEC M6 T-nuts can be used.

## Main frame construction

1. – 25. – Basic frame construction

26. – Adding wheels to frame

27. – Adding mounting brackets for floor plates

28. – 30. – Adding floor plates

31. – Adding hinged door panel (optional)

32. – Adding slot rollers

33. – Adding mounting profiles for screens, DAQ etc. (optional)


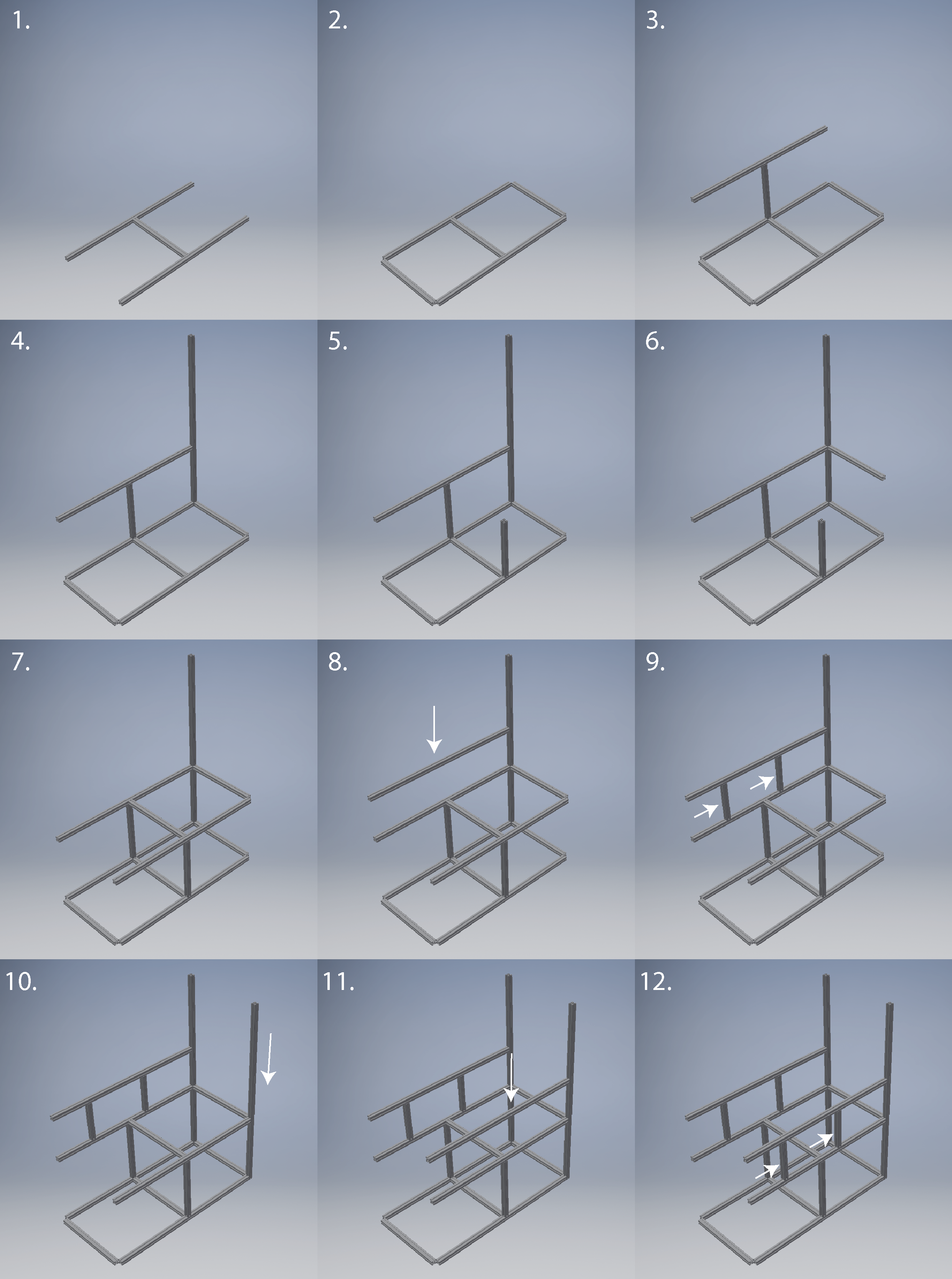


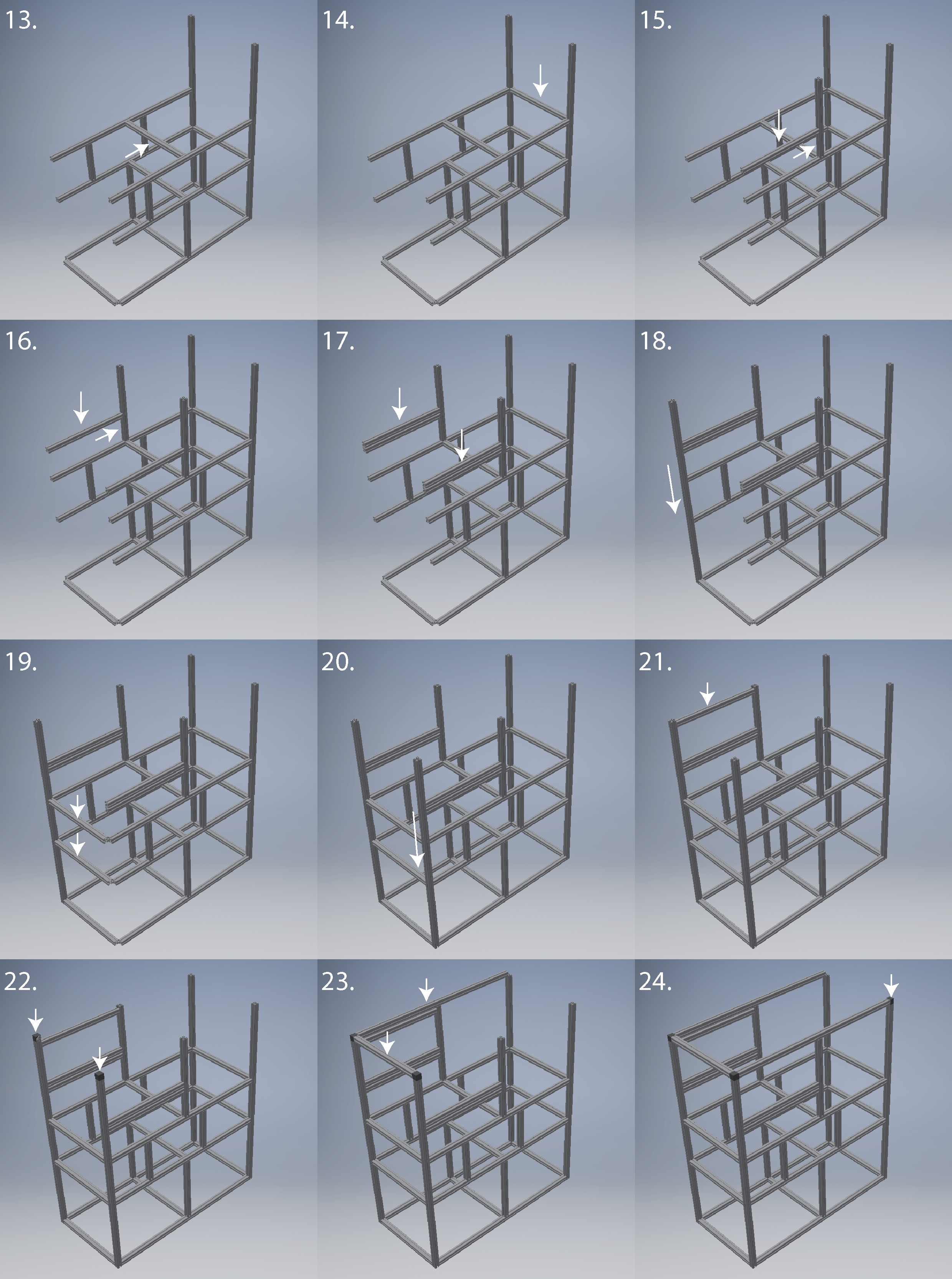


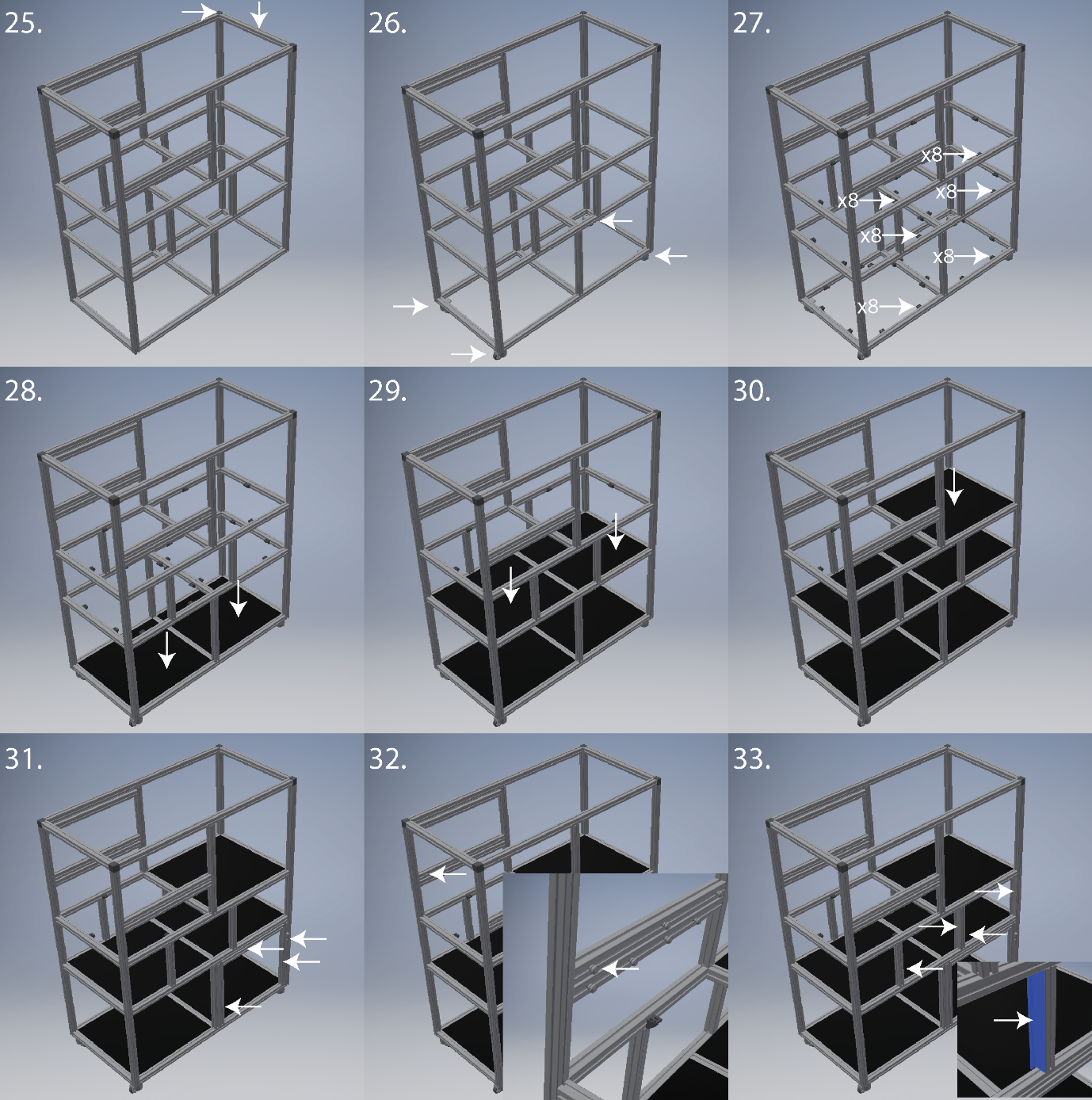


## Cleaning chamber construction

1. – 5. Basic frame construction

6. Adding floor plate

7. Adding guide rails

8. Adding top removable plate


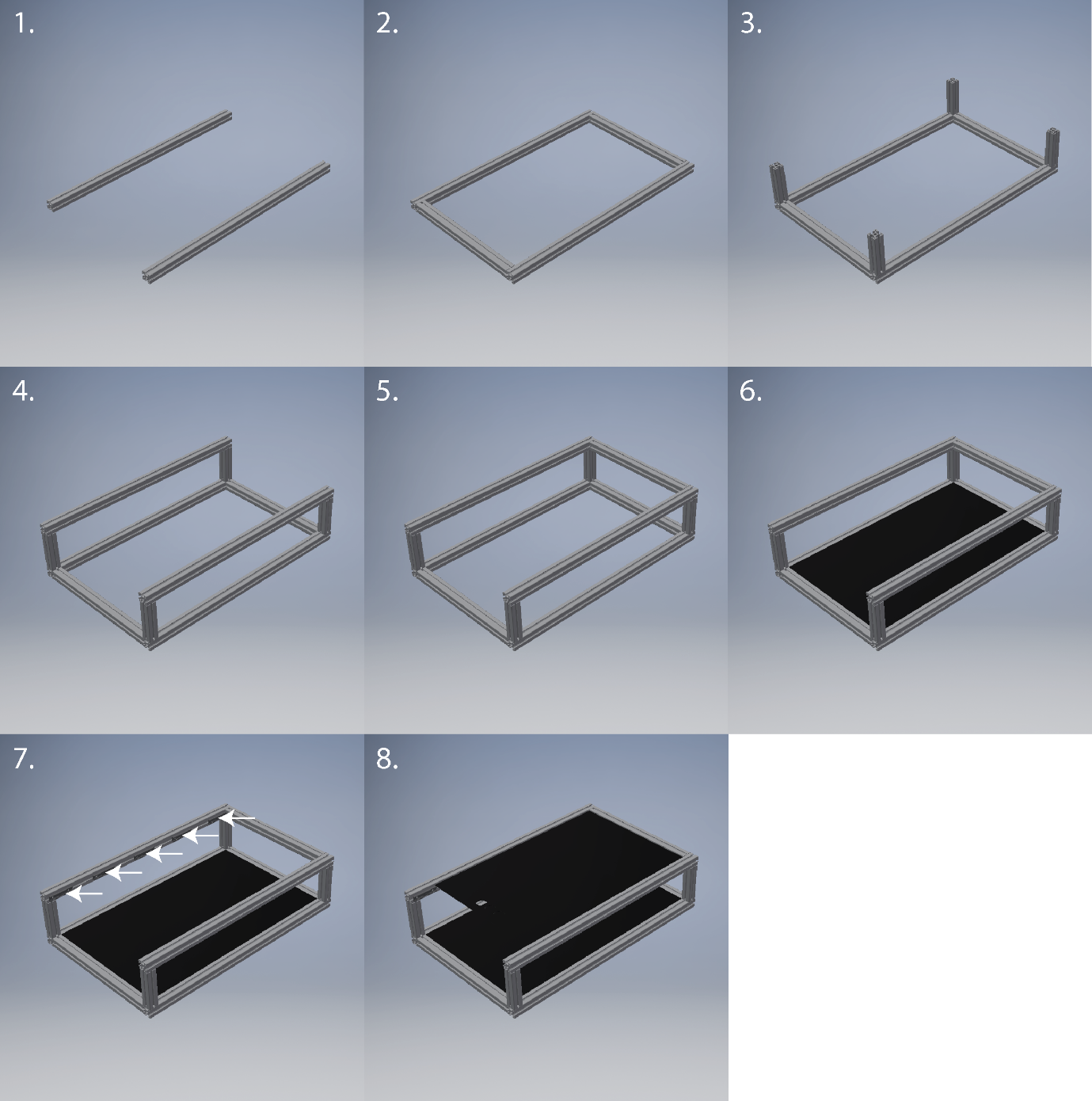


## Home cage construction

1. – 2. – Bottom frame construction

3. – Adding floor mesh

4. – 9. – Finishing frame construction

10. – Connector slots for wall panels

11. – Adding wall panels

12. – Adding removable floor plates


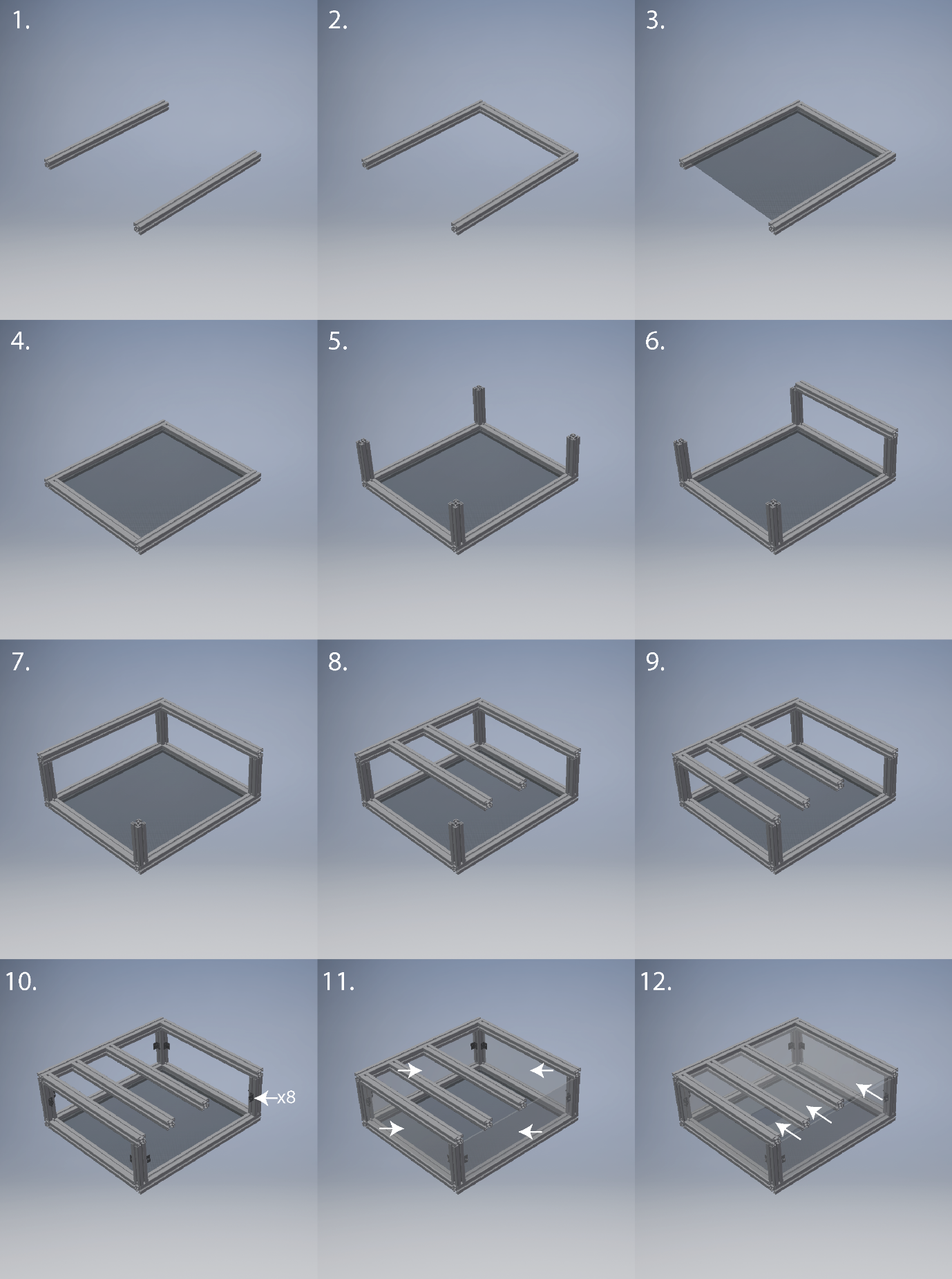


### Pre-chamber construction


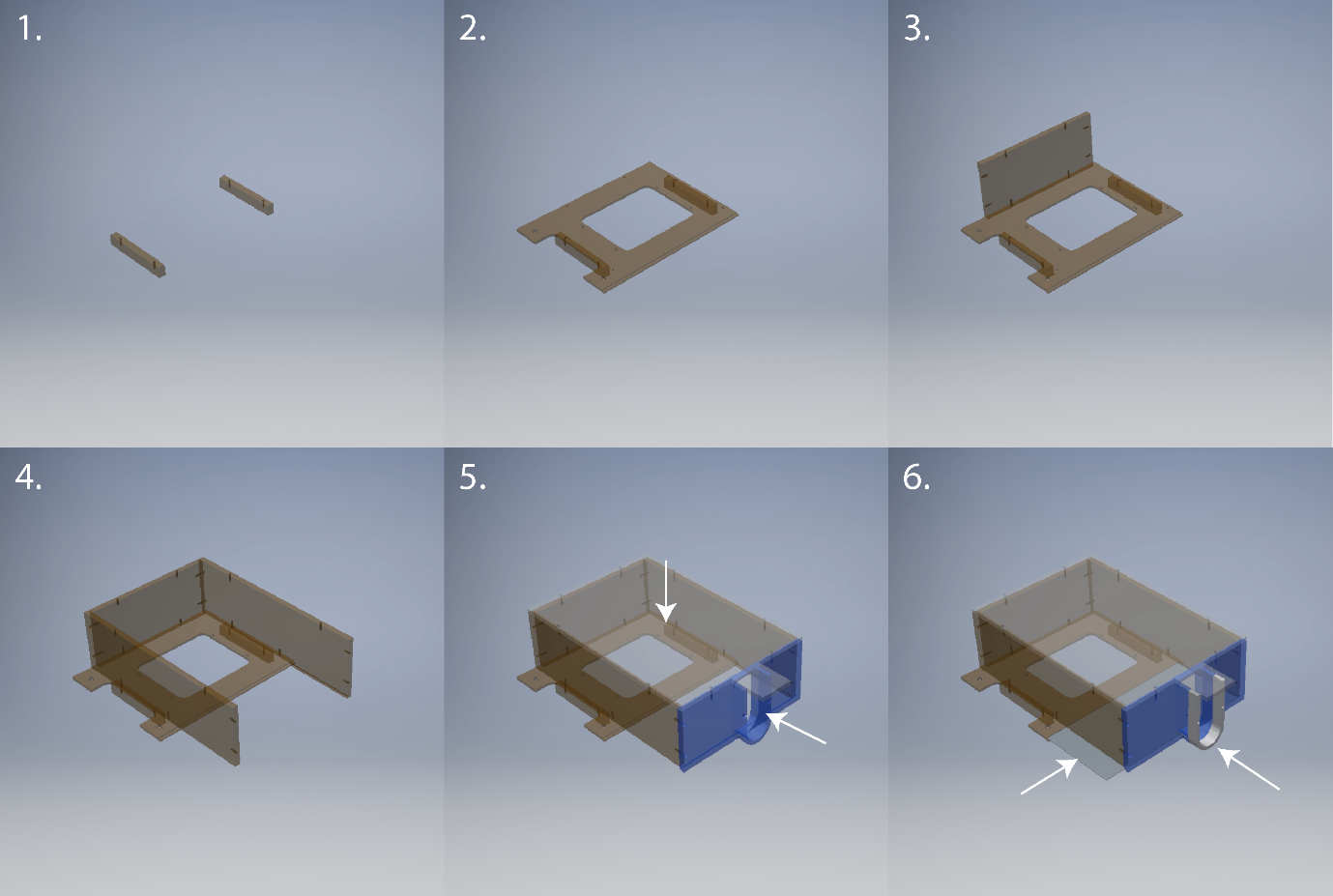


### Door mechanism construction


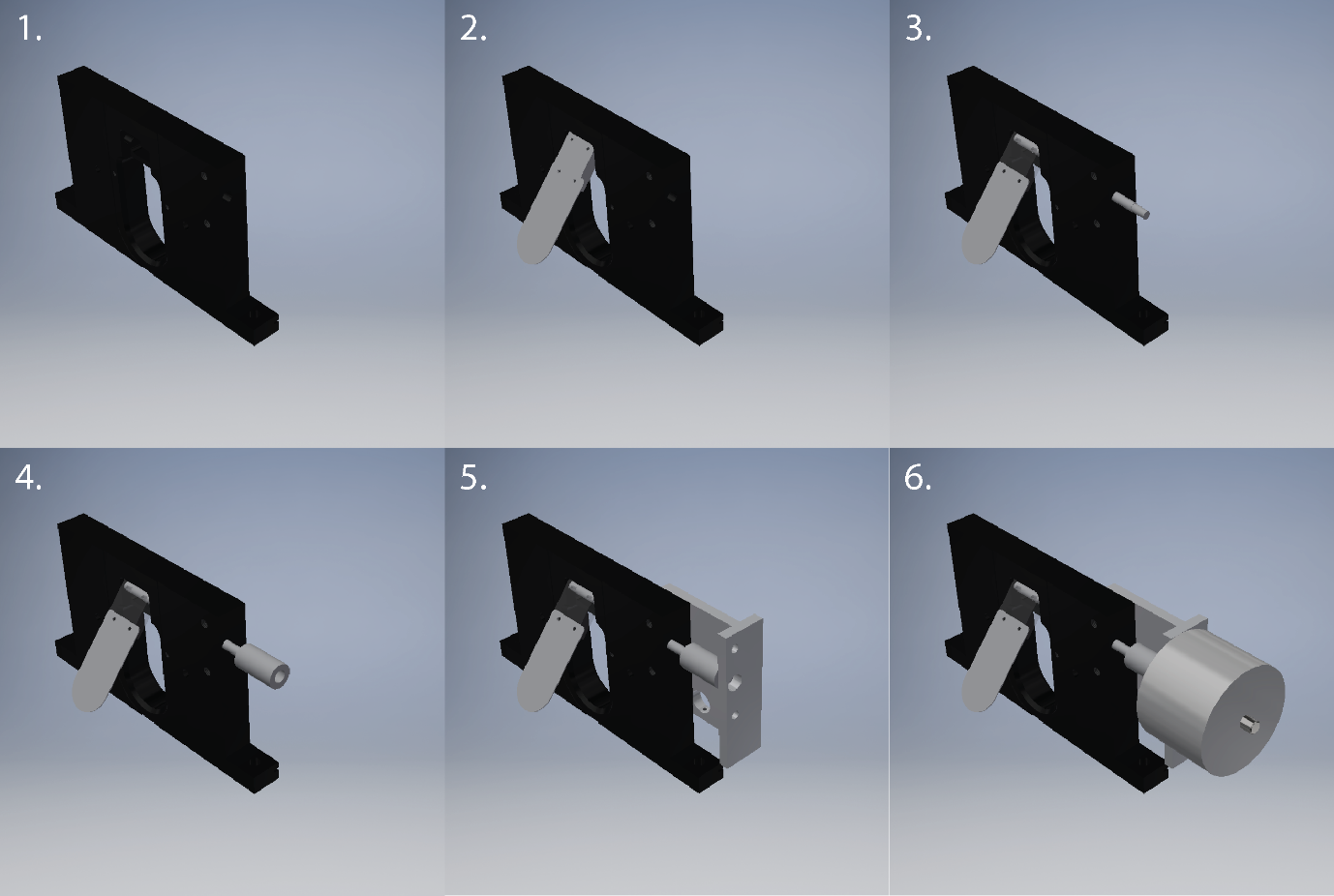


### Access tunnel and behaviour port construction


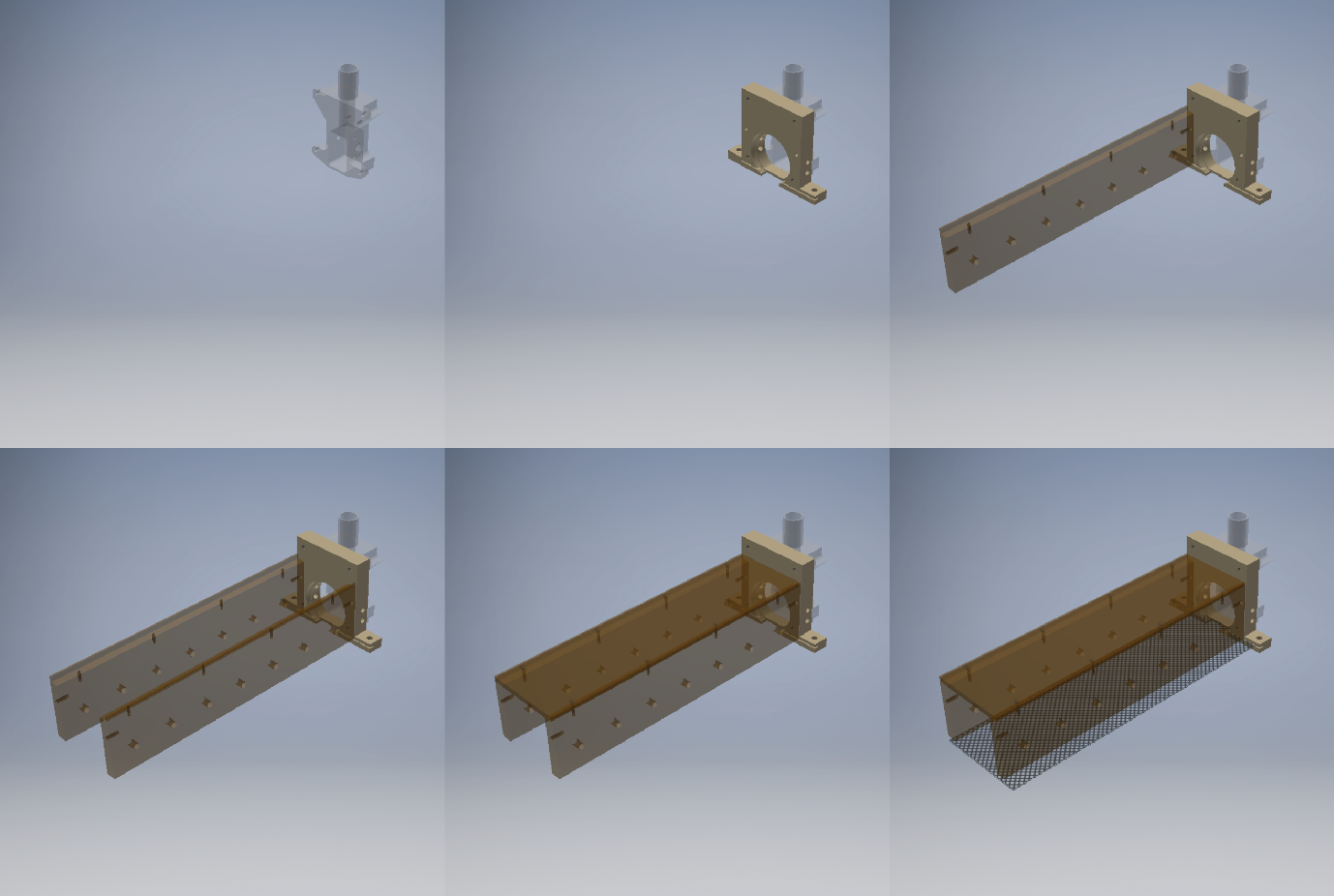


### Olfactometer


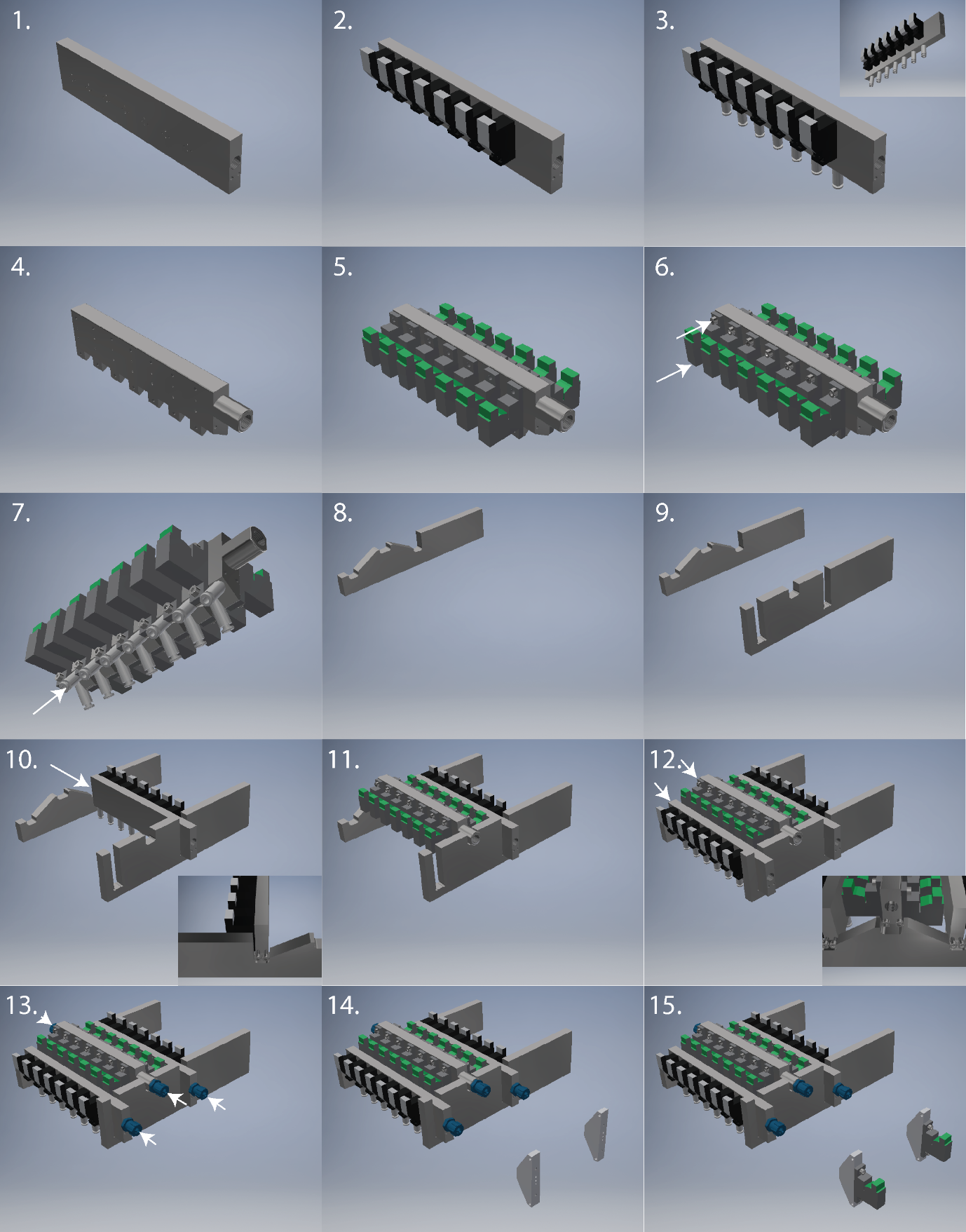


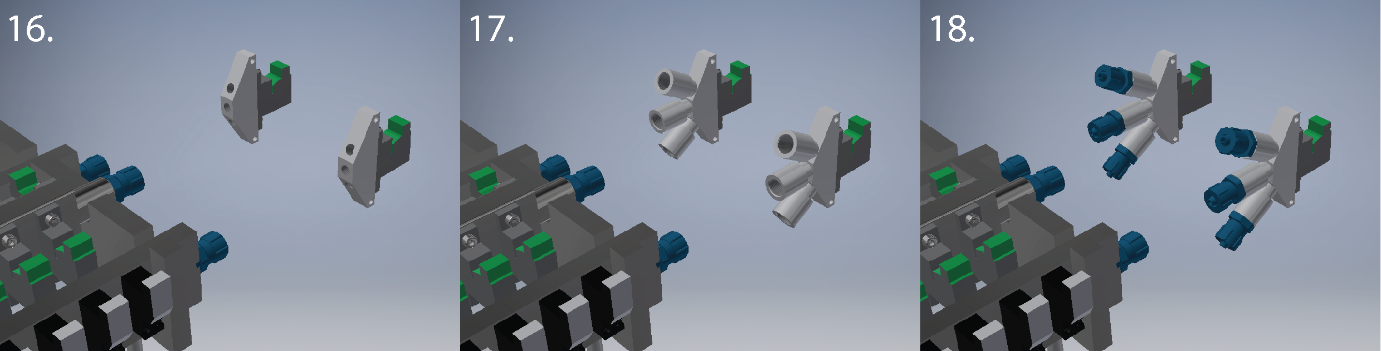


### Wiring / sensor connections

Once the system is constructed, certain modules and sensors must be connected for proper operation.

1. IR beam detection
   1. IR beam pairs must be installed facing each other in the housings of the access tunnel and behaviour port.
   2. Each IR beam pair is then connected to an input connection in the ir-logic module.
   3. For each IR beam pair required to trigger door closing, the corresponding signal out port should be connected to signal IN on the door-close module.
   4. Each IR beam that should trigger a trial start should be connected to a single analog input channel on the main DAQ board.
2. Door close mechanism
   1. Connect rotary magnet to rotary magnet connection in the door-close module.
   2. Connect any input triggers from the ir-logic module to the signal IN connection.
3. RFID decoder
   1. Install an RFID coil in the housing of the behaviour port.
   2. Connect the leads of the RFID coil to the decoder
   3. Connect the decoder via a USB-Serial interface to the main control PC
4. Lick detector
   1. Connect the lick detector to a 15V power supply.
   2. Connect the ground lead to the frame of the system
   3. Connect the signal lead to a conductive part of the tube which the animal will lick for water rewards
   4. Connect the signal OUT connection to an analog input channel on the main DAQ.
5. Stimulus production
   1. Connect stimulus production components (e.g. valves) to digital output channels on the main DAQ.
6. Water delivery
   1. Connect an analog output channel from the main DAQ to the analog input for the pump microcontroller

# Software manual

## Introduction

The AutonoMouse software is written in Python (and PyQt for graphical interface). There are two main components:

1. schedule-generator deals with generating sets of trials to be performed by animals in the system. Each trial is a set of digital commands which can be parsed into ADC output. This package is widget based, allowing users to define their own sets of parameters with which to generate trial sequences.
2. autonomouse-control keeps track of animals housed in the system and delivers behavioural trials (defined in schedule-generator) at appropriate times. This package is used to plan and execute large-scale behavioural experiments.

Currently, the software (autonomouse-control) is configured to work with national instruments DAQ hardware via NIDAQmx and the pydaqmx interface. All software is available from <https://github.com/RoboDoig/autonomouse-control> and <https://github.com/RoboDoig/schedule-generator>.

## schedule-generator

### Installation

Python version 3.5+ is required along with PyQt5. On Windows it is recommended to install a distribution such as WinPython as this includes the majority of dependencies.

Dependencies:

- numpy
- PyQt5
- pickle

Dependencies (available in repository):

- PyPulse

### Running the schedule generator

Open the schedule-generator main window by running ‘main.py’ in the home folder. The main window should load as shown below:


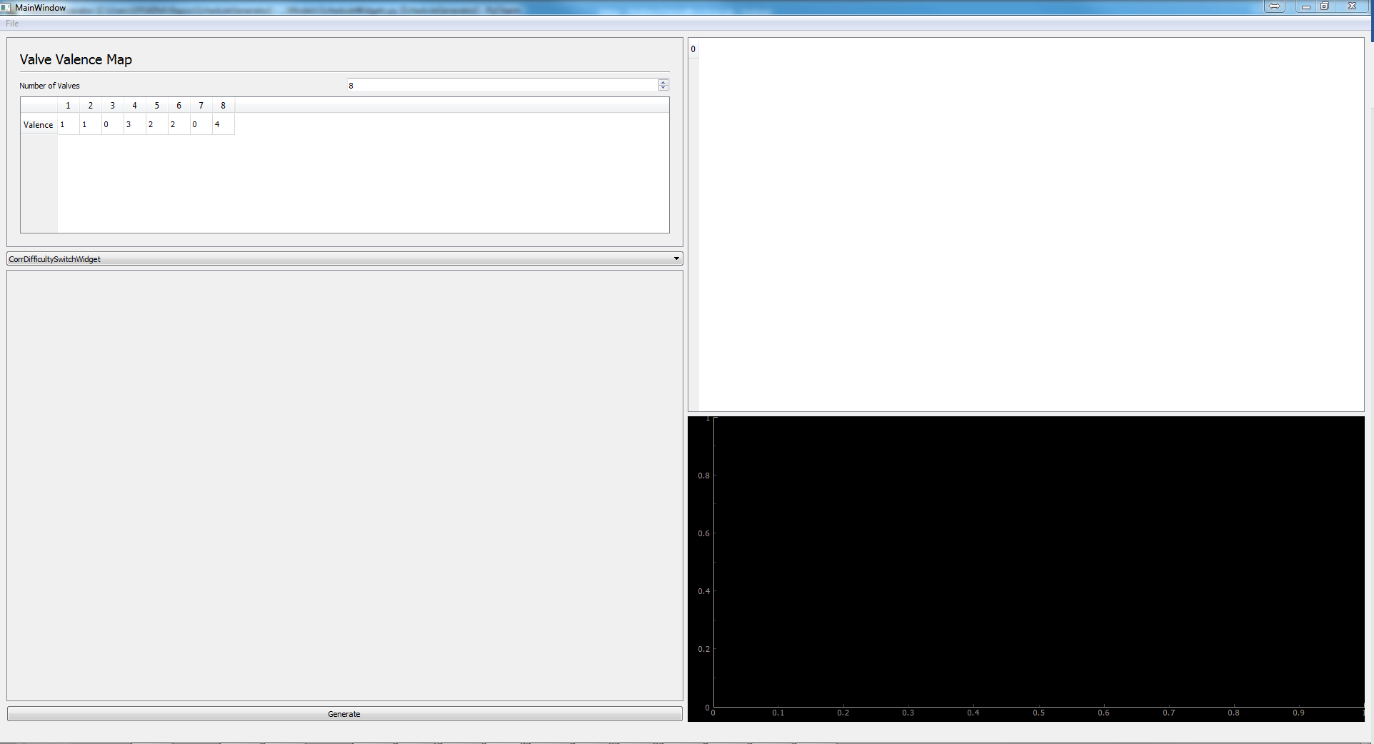


### The main window

The main window contains a number of control and display panels for generating and modifying schedules:


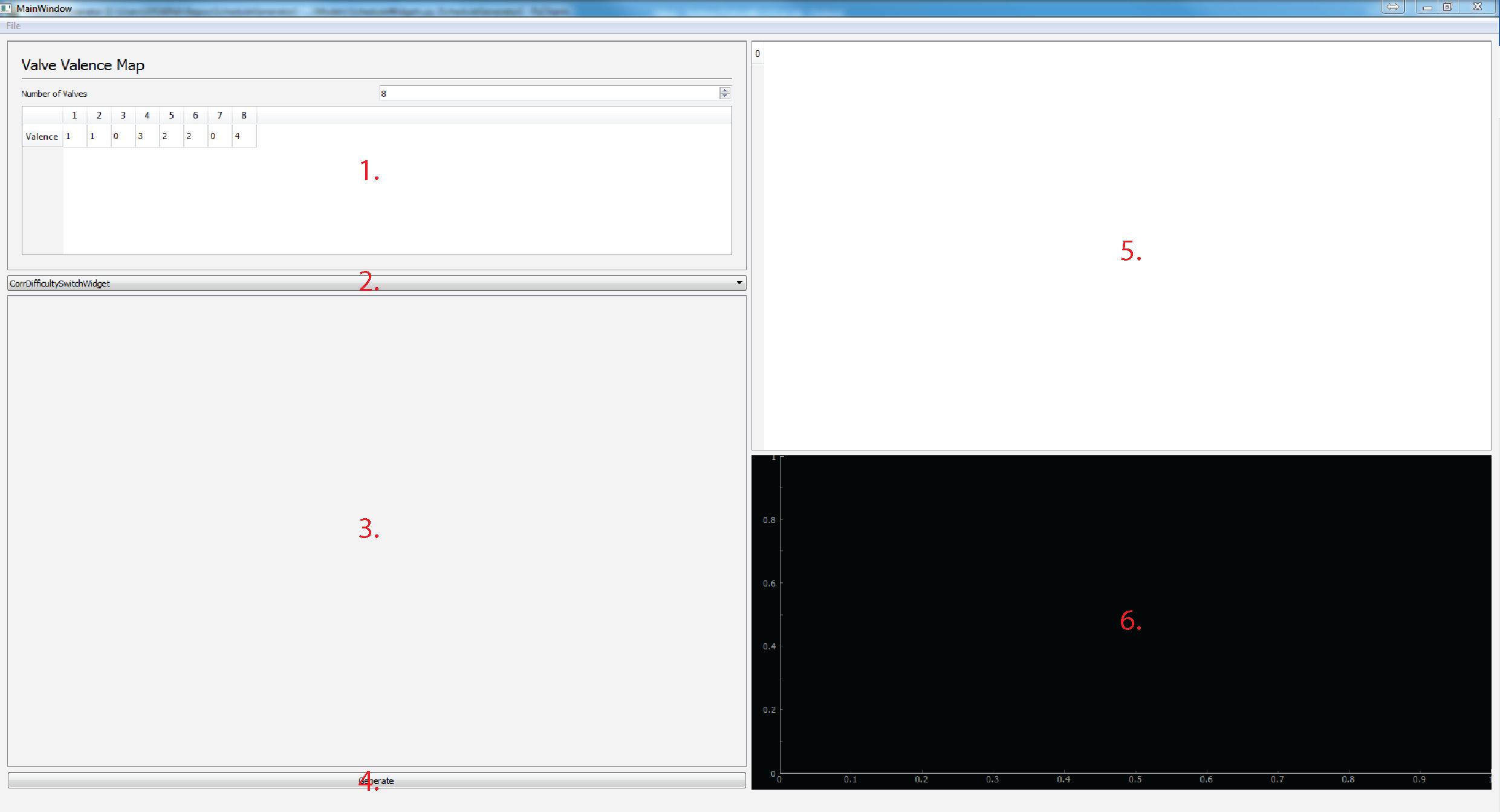


1. The valve valence map panel – this panel is used to specify digital channels to be used in a schedule, as well as any ID / valence they should be assigned.
2. Widget selector drop-down – this drop down allows the user to select from constructed schedule generation widgets
3. Widget display – this panel shows input parameters and additional information for the selected widget
4. Generate button – used to generate a schedule once input parameters have been defined
5. Schedule display – this panel displays the sequence of generated trials and allows the user to select individual trials for display
6. Trial display – this panel displays the constructed digital signal patterns of each selected trial

### Generating a schedule

To generate a schedule, first select an appropriate schedule widget from the drop down menu. Information about the chosen schedule type will then be displayed in the widget display panel, as well as any user defined input parameters, e.g. trial length. Once the desired parameters have been set, click ‘Generate’ and a sequence of trials will be generated based on the desired parameters. Use File >> Save to save this trial sequence for later loading in the main autonomouse-control program.

### Schedule generation example

In this example, we will generate a sequence of trials for a simple go/no-go (GNG) olfactory discrimination experiment. We will use the pre-defined SimpleGNGWidget which will take care of the majority of schedule and trial structure automatically. For our schedule, we will define 4 active digital channels (in this case corresponding to valves that release different odours).

First, set the number of valves to 4 to correspond to our active channels. Then assign valence to each of the channels to correspond to the odour controlled by each of the channels.
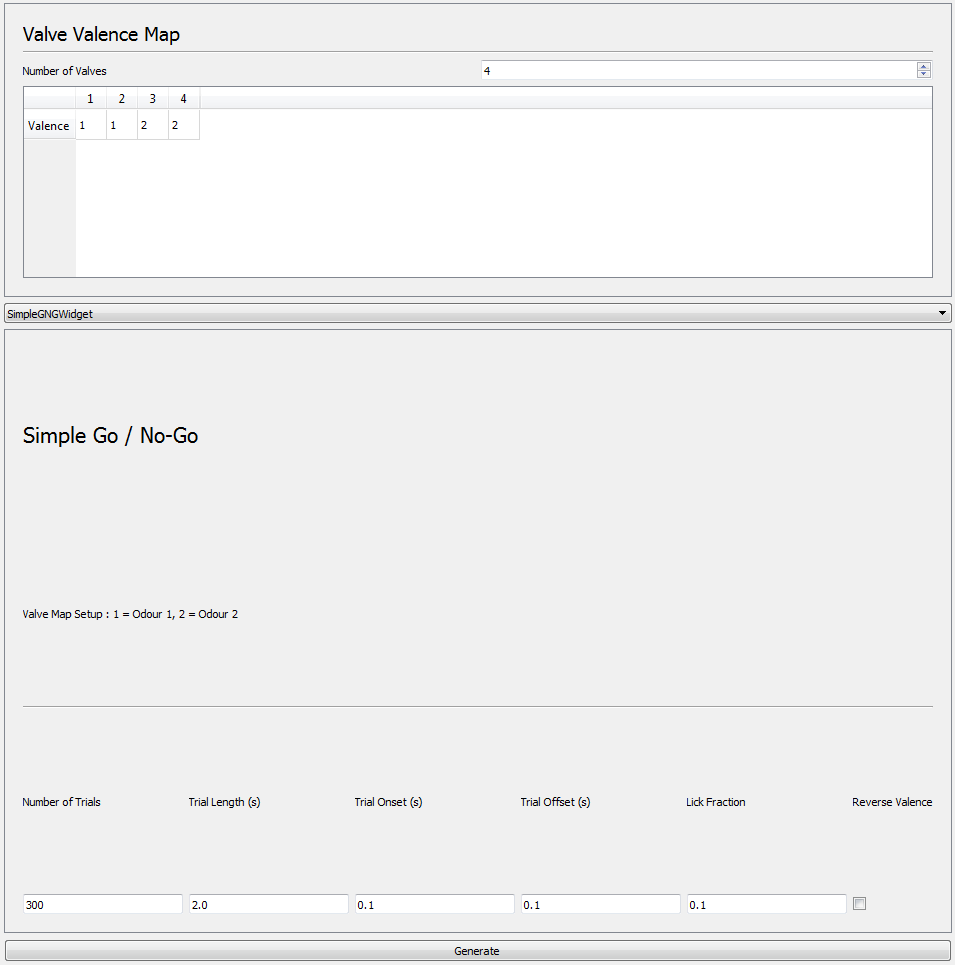


Next, load the SimpleGNGWidget using the drop-down menu. This will load the widget and display the input parameters.


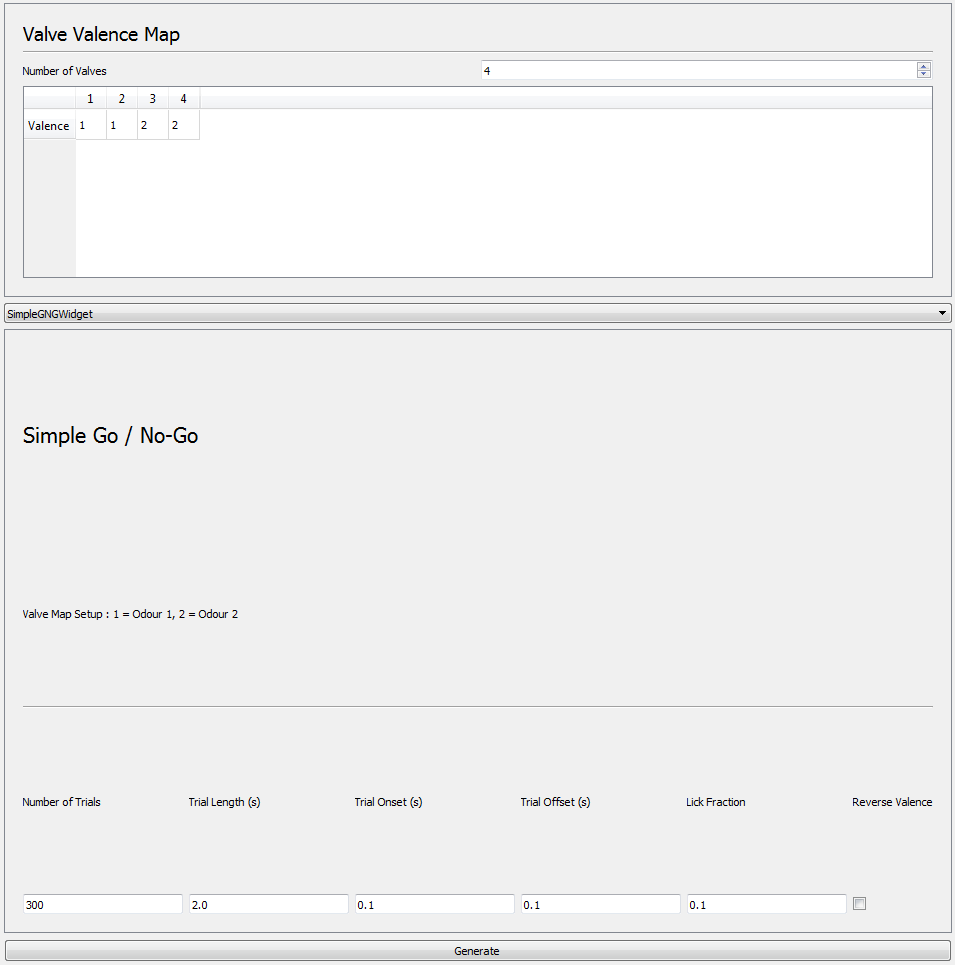


The SimpleGNGWidget has a number of static parameters (cannot be modified in the widget display) and another set of user-defined parameters. The static parameters for this widget take care of certain features of a GNG experiment that will essentially always be desired. For example, this widget randomises the sequence of rewarded and unrewarded trials, but ensures that there is only ever a maximum of 3 trials in a row of each condition. The widget will also randomise which channel is picked for the rewarded or unrewarded odour, depending on the defined valence in the valence map. In this case as we have odour 1 on channels 1 and 2, when odour 1 is required for a trial it will be randomly picked from one of these two channels.

We have a small choice of user-defined parameters for this widget:

- Number of trials: the total amount of trials to be generated for the schedule
- Trial length: the total stimulus time for each trial
- Trial onset / offset: the pre-/post-stimulus time
- Lick fraction: the fraction of total trial time an animal must lick for its response to be considered positive. In the above image, lick fraction is set to 0.1, so the animal must lick for at least 10% of the total trial time for its response to be considered positive.
- Reverse valence: by default, odour 1 will be the rewarded odour and odour 2 will be unrewarded. Checking this box reverses this reward contingency.

The widget also displays some basic information at the top, showing us that a value of 1 in the valence map will assign a channel to odour 1, while a value of 2 will assign a channel to odour 2. Therefore, in our current configuration, channels 1 and 2 will control odour 1, while channels 3 and 4 will control odour 2.

Generate the schedule by clicking the ‘Generate’ button. This will generate the schedule based on our parameters, and display the trial sequence in the schedule display panel.


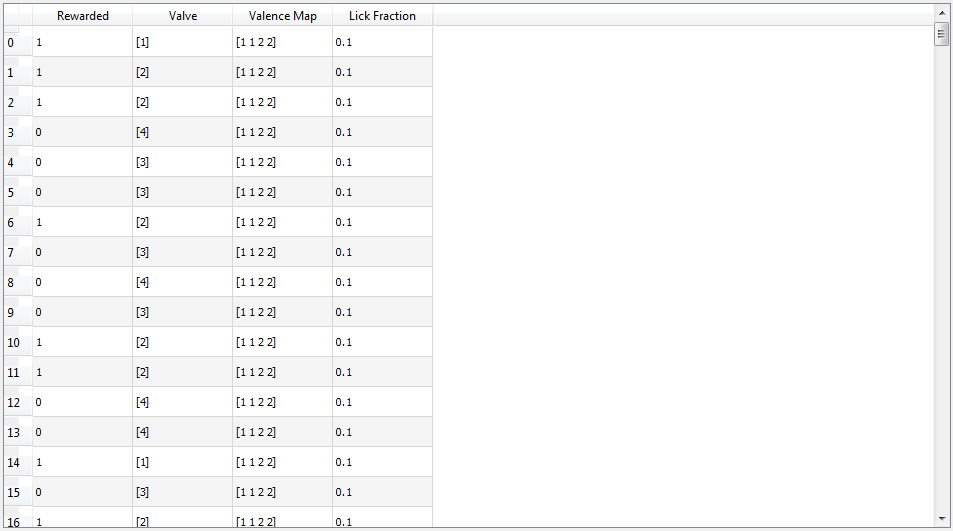


Here we can see the chosen values for each trial that will define its structure. We can see, for example, that all rewarded trials (indicated by a 1 in the corresponding column) use valves 1 or 2 (corresponding to odour 1 based on our valence map). Clicking on a trial will show its structure over time in the trial display panel.


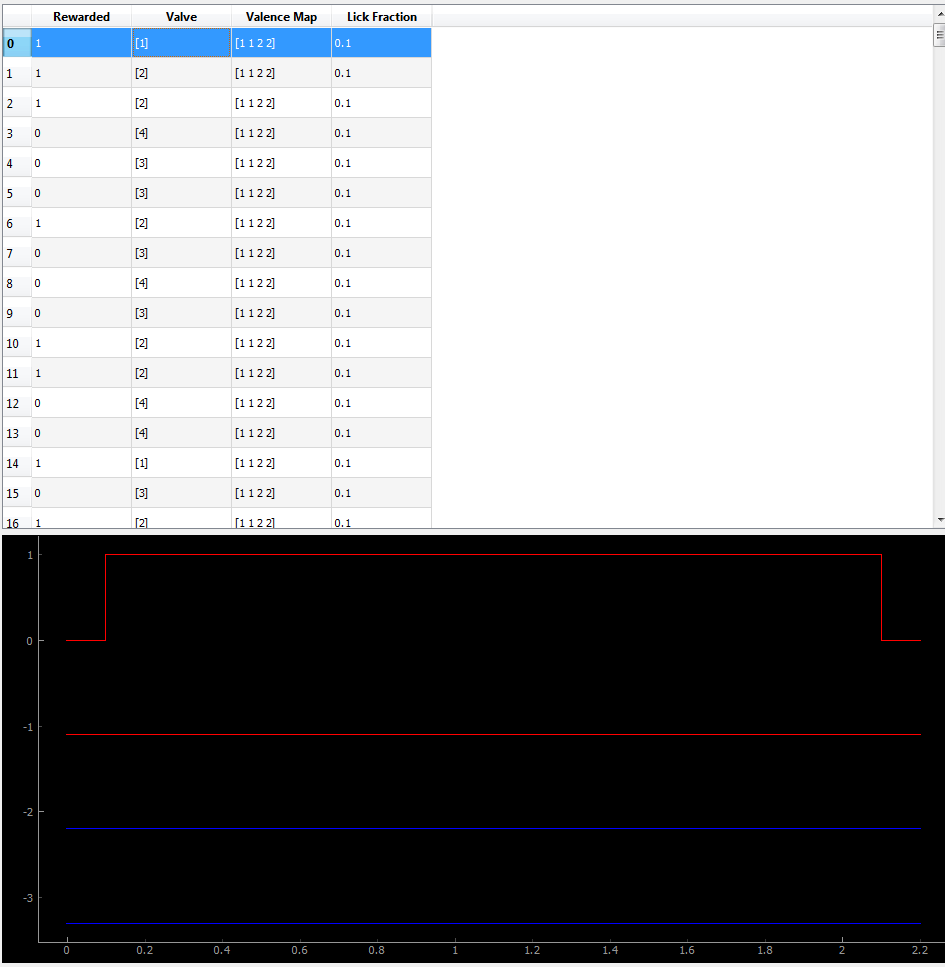


Here we have selected the first trial. In the trial display panel we can see all four active channels, colour coded by their assigned valence (red = odour 1, blue = odour 2). As this is a rewarded trial, we want to deliver odour 1. In this generation, channel 1 was selected to deliver this odour. In the trial display panel, we can therefore see that channel 1 is active for 2s, while all the other channels are inactive. Clicking on other trials will also show how they are structure, for example we can select an unrewarded trial which – as expected – utilises a channel assigned to odour 2.


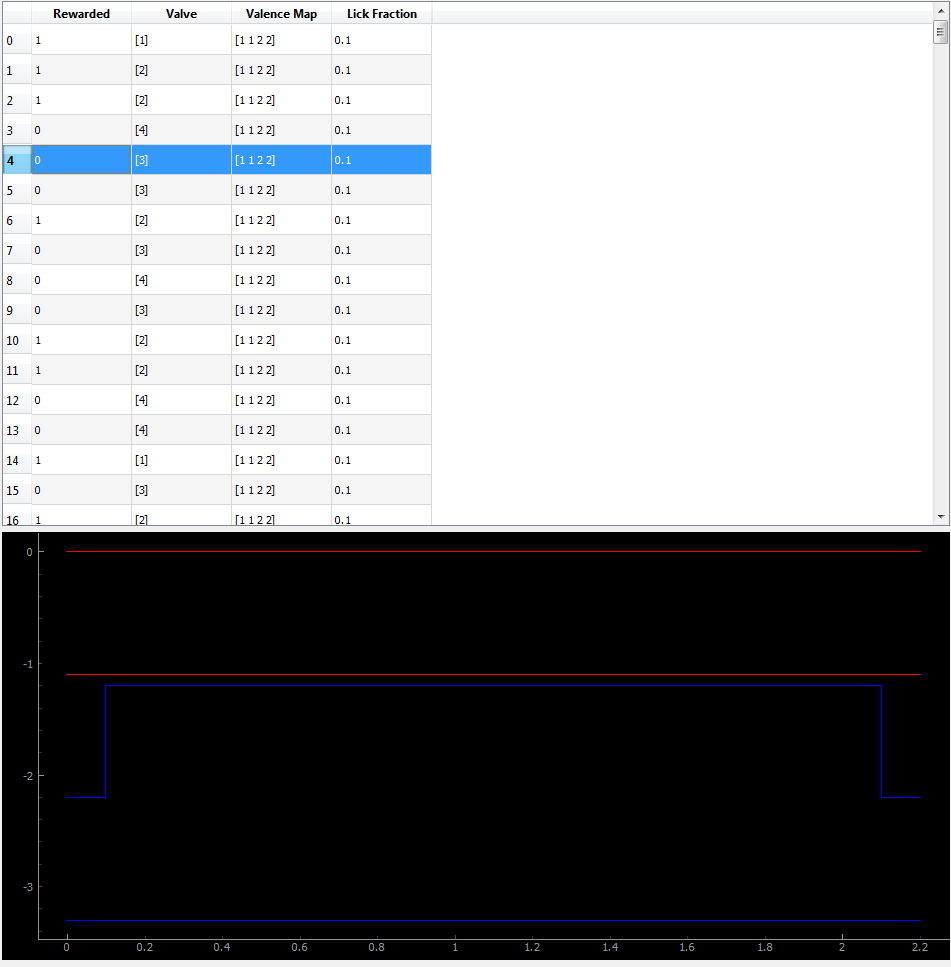


This schedule can now be saved for later utilisation in an AutonoMouse experiment.

## autonomouse-control

### Installation

Python version 3.5+ is required along with PyQt5. On Windows it is recommended to install a distribution such as WinPython as this includes the majority of dependencies.

Dependencies:

- numpy
- PyQt5
- pickle
- datetime
- time
- scipy

Dependencies (available in repository):

- PyPulse
- daqface

### Running autonomouse-control

Open the AutonoMouse control software by running the ‘main.py’ file in the home folder. The main window should appear:


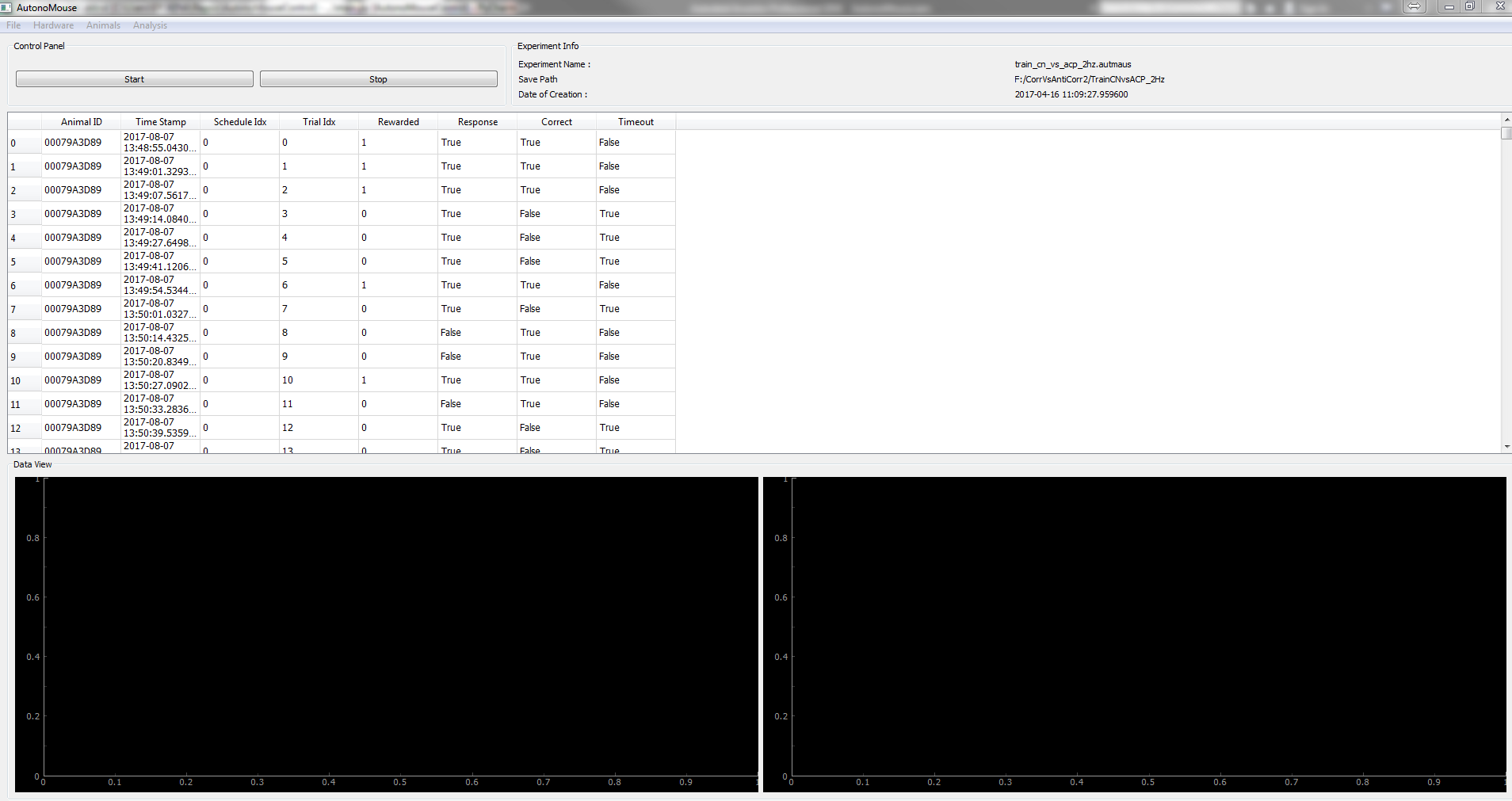


### The main window

The main window contains a number of display elements for monitoring experiment progress. The control panel provides user control for starting and stopping experiments. The experiment information panel displays name and save location of experimental data as well as the date of initiation of an experiment. The central display shows a list of all trials performed and information on the results of each. The bottom data view panel shows the digital signal pattern used to generate a trial stimulus and the lick response of mice in the system.

### Hardware control

The hardware control panel can be accessed by clicking through Hardware >> Hardware Preferences:


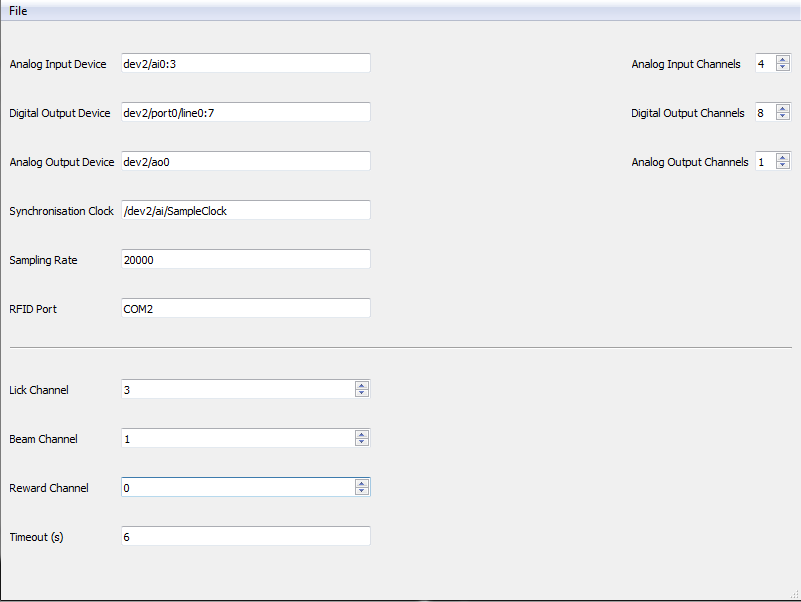


A number of general preferences and hardware routes can be specified here. N.B. that specification for hardware channels (National Instruments) used standard NIDAQmx syntax:

- Analog input device – specify the analog input channels used to read sensor data
- Analog input channels – the number of channels that the analog input device refers to
- Digital output device – specify the digital output channels used to produce trial stimuli
- Digital output channels – the number of channels that the digital output device refers to
- Analog output device – specify the analog output channels used to drive water delivery or other actuators
- Analog output channels – the number of channels that the analog output device refers to
- Synchronisation clock – the internal NI clock used to synchronise the analog input and digital output.
- Sampling rate – the sampling rate in Hz used to sample sensor data and output digital signals
- RFID port – the COM port used to read the RFID decoder
- Lick channel – the analog input channel that will read the lickometer data
- Beam channel – the channels that will read IR beam breaks to detect animal presence
- Reward channel – the analog output channel used to trigger water reward
- Timeout – the time in seconds an animal is in timeout following incorrect licks on S- trials

### Animal management

Clicking through Animals >> Animal List opens the animal management display window. This interface is used to add animals to an experimental cohort and track general indicators of their performance.


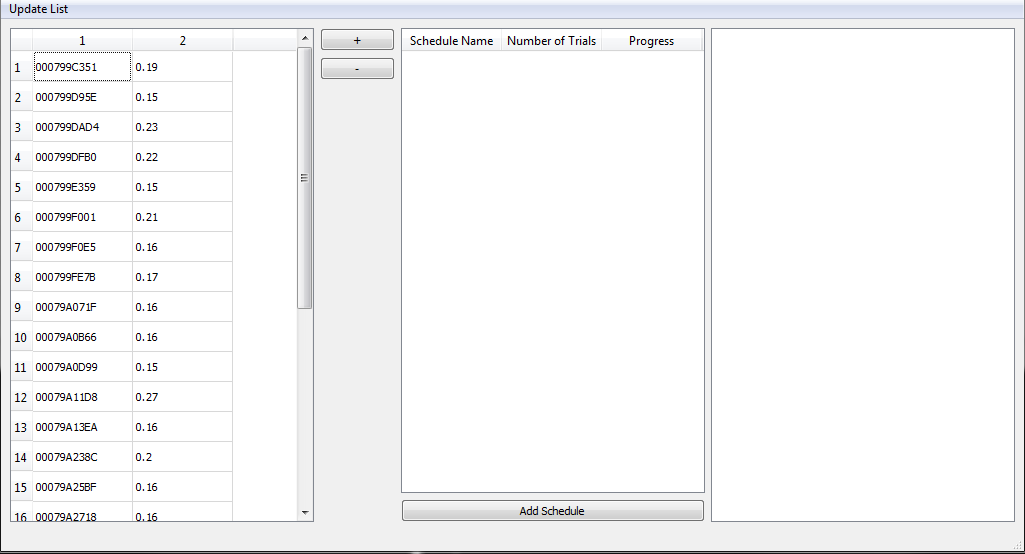


The leftmost panel shows a list of animals present in the experimental cohort. Every experiment must include a ‘default’ animal. The default animal schedule is triggered in the event that an animal is detected in the behaviour port but RFID decoding fails. Animals can be added or removed from the cohort with the ‘+’ and ‘-‘ buttons next to the display. On adding an animal, its RFID code should be specified in the left column, and the amount of water it should be delivered per trial in the right column. For each animal, schedules of trials can be added using the central window and the add schedule button. This window displays the list of names of added schedules, how many trials are present in each schedule and the animals’ progress through each. The rightmost panel displays detailed information for individual trials within each schedule.

### Analysis

Basic analysis of animal performance can be accessed through Analysis >> Analyse Experiment. This display window contains a table entry for each animal in the experimental cohort with the total number of trials it has performed in the current experiment, as well as the number of trials performed in the last 24 hours. Clicking on an animal entry calculates performance over time for this animal (according to bin size) and the average performance of the whole group.

**
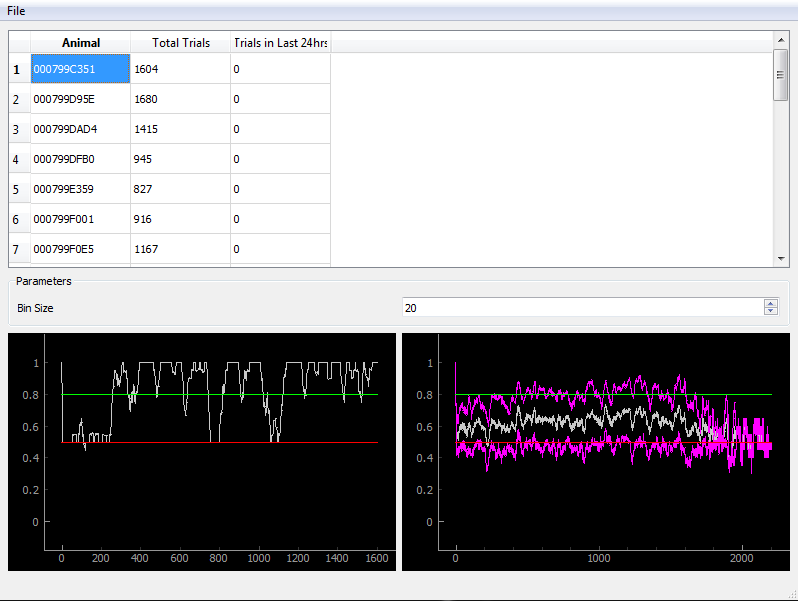
**

### Example experiment

In this example we will prepare a simple AutonoMouse experiment to demonstrate the steps required. First, we will prepare two schedules using schedule-generator. One will take the form of GNG odour discrimination with random concentrations, as produced in the schedule-generator chapter. The other will be a pretrain-type schedule to be assigned to the default animal (in the case RFID code cannot be read). It is always important to assign a default schedule in AutonoMouse experiments as if there is a fault with an animal’s RFID it should still be able to get water rewards.

First open schedule-generator and assign the valence map. We will here assume a 2-channel olfactometer with odour 1 in the first position, odour 2 in the second position, and blank (mineral oil) positions for the remaining channels. Then load the PretrainWidget and generate a pretrain schedule. For this example we will create a 300 trial pretrain schedule, with length of 2s, onset and offset of 0.1s and assign a lick fraction of 0.1 (so the animal still has to lick for water rewards even if its RFID is not detected.


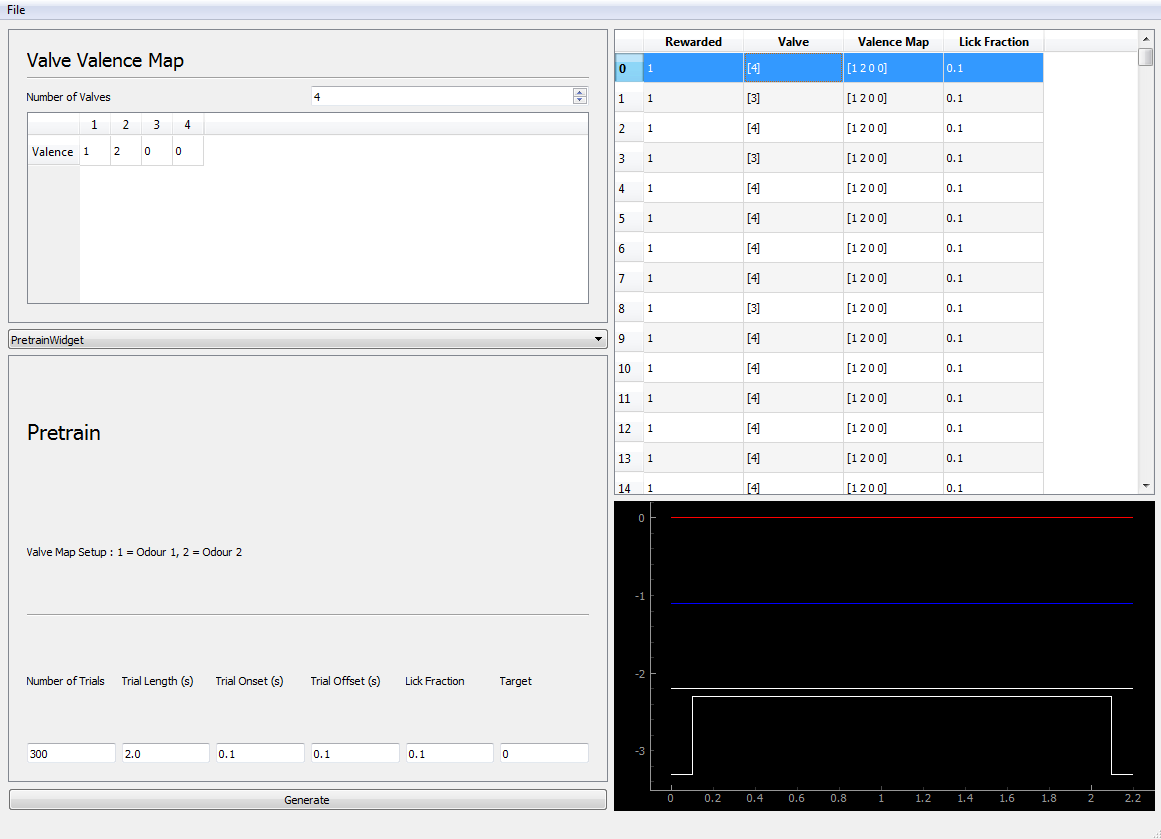


Save this schedule with a descriptive name (e.g. Pretrain). To generate the main behavioural schedule, load in the ConcGNGWidget and generate a schedule. We will use the same parameters as for the pretrain schedule and use the default parameters we set in the schedule-generation chapter.


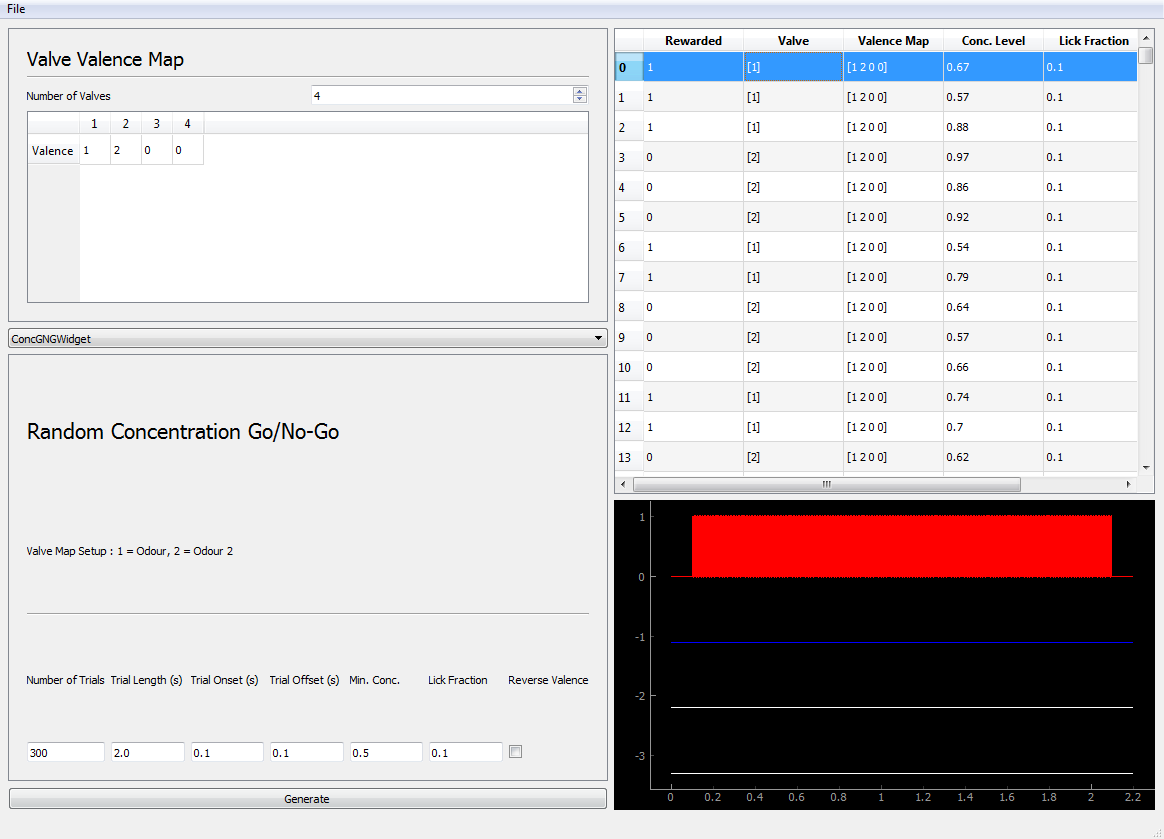


Save this schedule with a different name (e.g. concGNGSchedule). Close the schedule-generator and open autonomouse-control. First, we must update the hardware preferences to account for the DAQ hardware and digital channel numbers we are using. Open Hardware >> Hardware Preferences. Adjust the preferences according to the physical address of the utilised DAQ hardware and signal ports used for sensors and RFID decoding. Make sure that the digital output device and number of digital output channels reflects the schedules we are using (in this case 4 digital channels for each of our olfactometer positions).


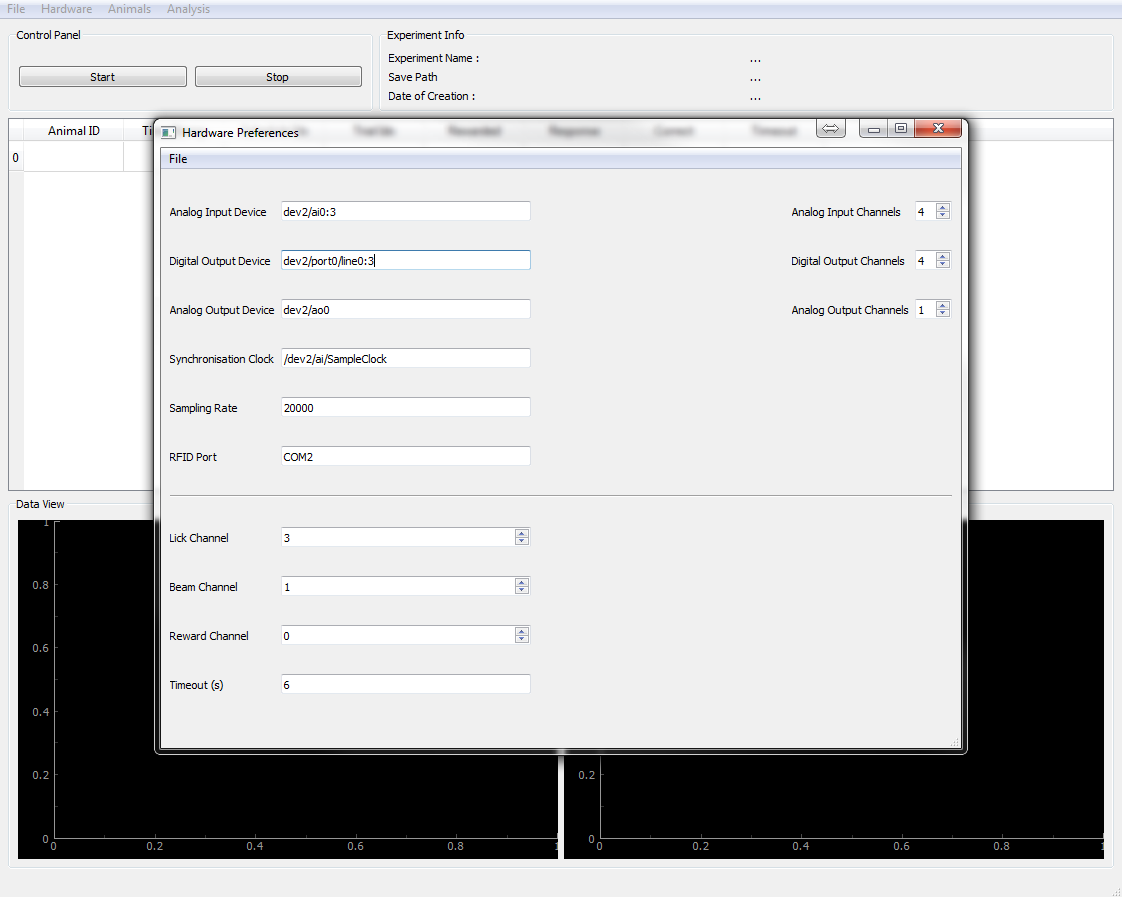


Save these preferences with File >> Save Preferences. Then close the preferences window and save the experiment with File >> Save Experiment.


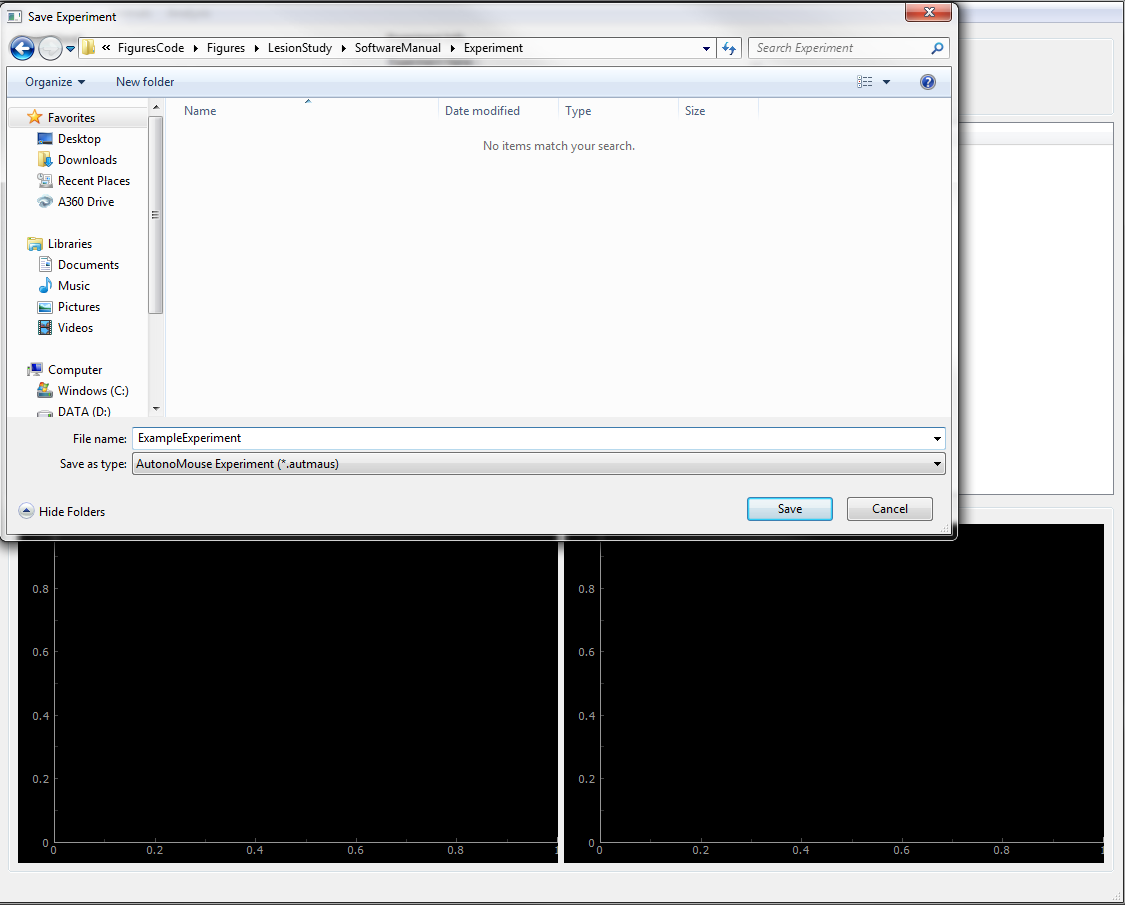


Next we will add experimental animals and assign schedules. Open the animal database with Animals >> Animal List. Add entries in the animal list according to the number of experimental animals and fill out their names (RFID code) and desired water reward amount per trial.


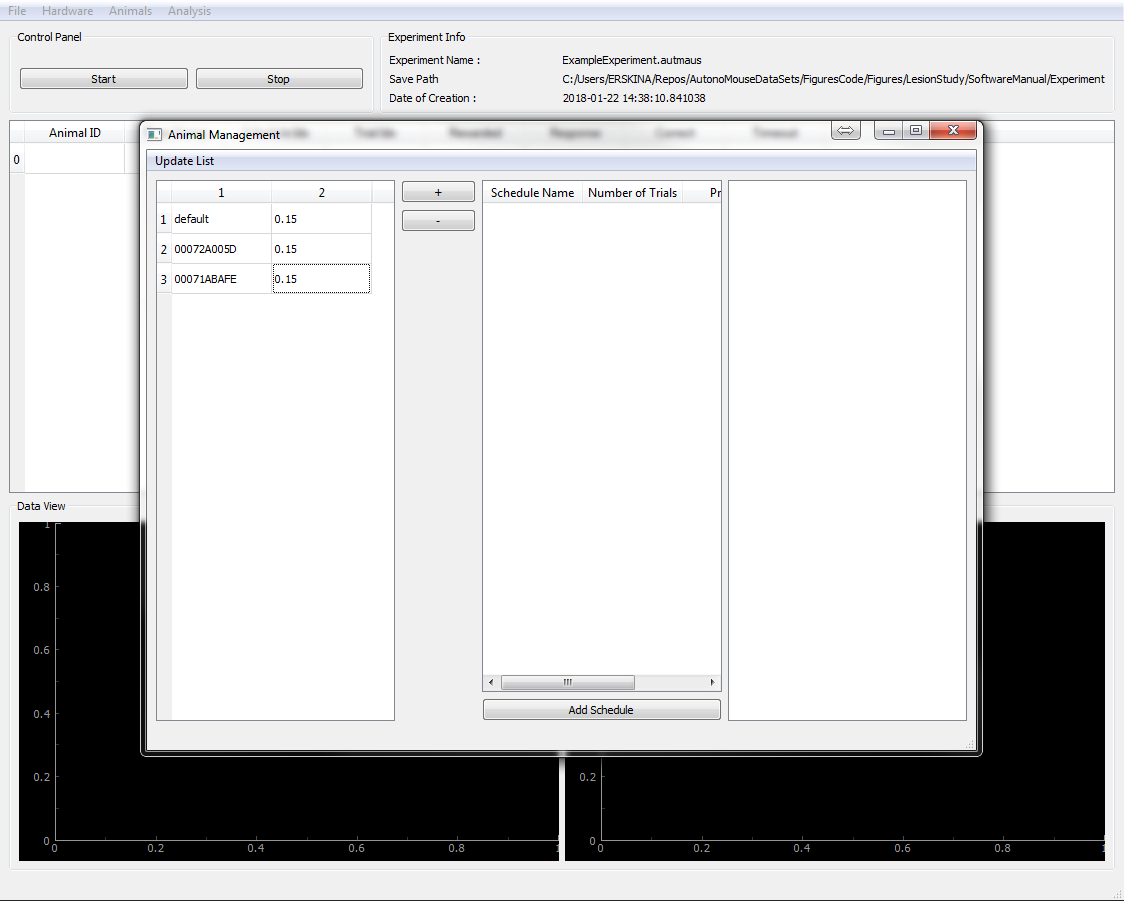


Now assign the pretrain schedule to the default animal by clicking on it, then on add schedule.


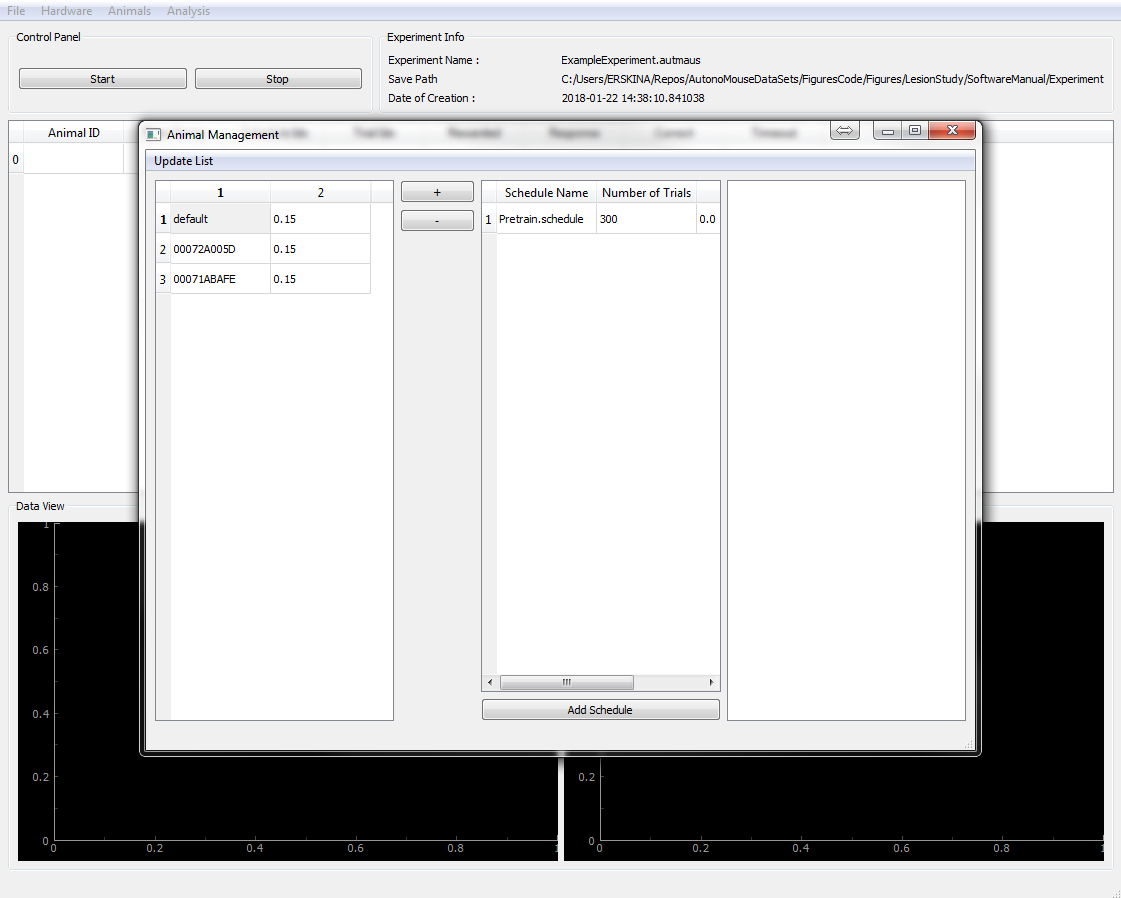


Note that we only need to add 1 schedule instance if we want these animals to perform this schedule for the duration of the experiment. Once the animal completes all 300 trials in the schedule, if no later schedule is queued then the current schedule will be automatically added and the animal will be able to continue to perform. Also note that the automatically added schedule will be an exact duplicate, and the assigned trials will be identical and not procedurally, randomly generated as in schedule-generator. A greater number of random trials can be achieved either by setting the trial number higher in schedule-generator, or by generating many different schedules and adding them all in the animal database.

Next we will add schedules for the behavioural animals. For this example we will assign our animals to perform 300 pretrain trials, and then ‘graduate’ them onto the GNG task. Click on the animals and assign them these schedules as before, now adding first the pretrain schedule and then the GNG schedule.


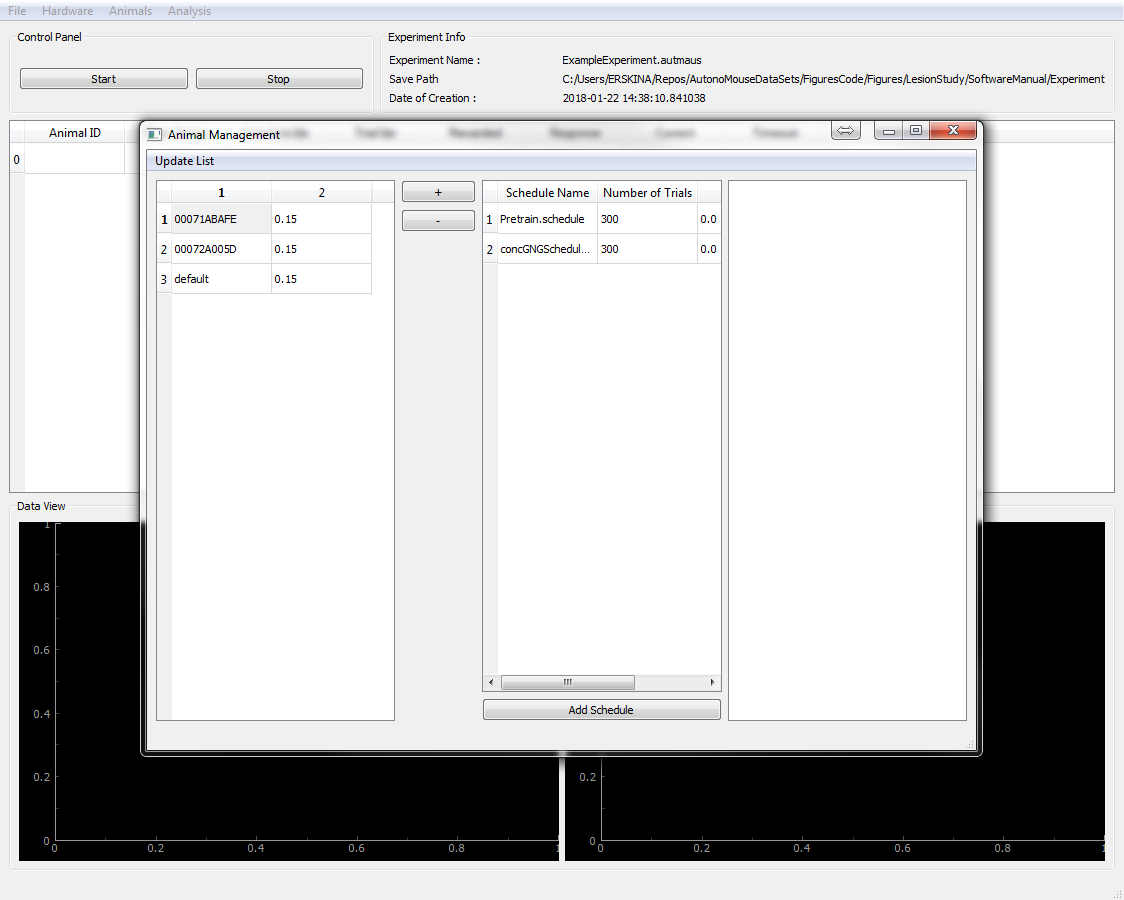


Update the animal database with Update List >> Confirm. Save the experiment again with File >> Save Experiment to lock in all changes. The experiment is now prepared and can be started with the Start button in the control panel.

The experiment can be stopped at any time with the Stop button in the control panel, though note that if any trials are being performed they will be completed before the experiment stops. The experiment will automatically save as trials are generated so no manual saving is generally required as the experiment progresses. In addition to saving the experiment, the sensor data for each trial will be saved in individual files for later analysis.

### Exporting data

Autonomouse-control contains a conversion script for converting the experiment structure into a more easily analysable MATLAB format. The first step in conversion is to generate a ‘schedule map’. This is a file that is used to automatically rename schedules in the experiment. This is desirable as in many cases of AutonoMouse experiments animals will be performing schedules with different names such that they can be differentiated in the software, but these schedules may have the same underlying structure. For example, all animals might be assigned to perform a GNG olfactory discrimination task, but schedules might be assigned to different groups such that some animals perform odour 1 as rewarded, and other animals as odour 2 rewarded, thus they are performing virtually the same task but their schedules are named differently (e.g. GNGOdour1, GNGOdour2). If it is desired to analyse these animals as a group all performing GNG, we may wish to rename these schedules collectively as GNG. The ScheduleMap is a .csv file, a ScheduleMap for the above example would take the form:


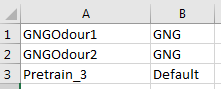


Column A is the names of the schedules within autonomouse-control. Column B is the desired renaming of each schedule. Make sure to save the ScheduleMap within the same folder as the AutonoMouse experiment for detection by the conversion script. The experiment data can then be converted with the Conversion script in AutonoMouse control, by adjusting the parameters of the ‘convert’ function. For our example experiment, conversion would be done as below:

batch_convert(['C:/Users/ERSKINA/Repos/AutonoMouseDataSets/FiguresCode/Figures/LesionStudy/SoftwareManual/Experiment'], 'C:/Users/ERSKINA/Repos/AutonoMouseDataSets/FiguresCode/Figures/LesionStudy/SoftwareManual/Experiment', 'convertedData', 4, save_licks=False)

The batch_convert function takes as its arguments:

1. A list of the experiment folder locations for the experimental data to be converted
2. Folder location for storage of the converted data
3. The desired name of the converted data file
4. The index of any additional trial parameters to be saves with the standard behavioural data
5. A Boolean specifying whether to also save lick times for each trial in the final data structure (will slow down conversion significantly if set to True).

## Advanced schedule-generator usage

### Schedule widget creation example

For more complex schedule structures we may wish to define new widgets with their own internal trial structure logic. To do this, we must generate a visual interface for adjusting user-defined parameters within schedule-generator. We must also create the logic for the trial structure of the schedule (how trial parameters will be generated, e.g. how many rewarded trials in a row) and how the parameters of each trial are translated into digital signals.

To demonstrate this, we will extend the simple GNG task to randomise the concentration level of the odour on each trial. For this example, pulse-width modulation of the digital signal will be used to define the concentration level.

First, we will design the visual interface for the widget. Open QtDesigner and create a new empty Widget.


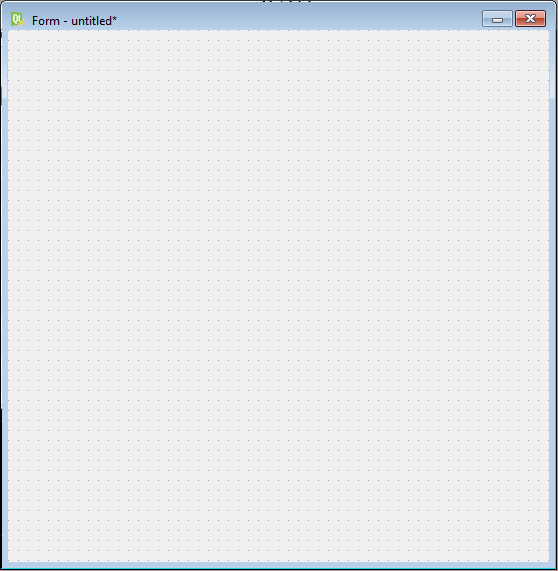


We will call the new schedule widget ‘ConcGNGWidget’. Drag in a label for the widget title and set the text to describe the widget. Right click on the widget body and select Layout >> Lay Out in a Grid to ensure all items are aligned. Save the widget in the schedule-generator’s UI folder as ‘ConcGNGUI.ui’


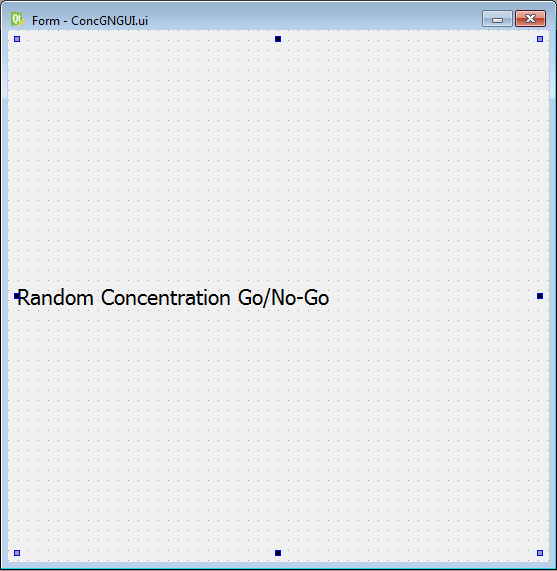


Add another label underneath for basic widget information, and a horizontal line spacer to separate text labels from user parameters which will be placed underneath.


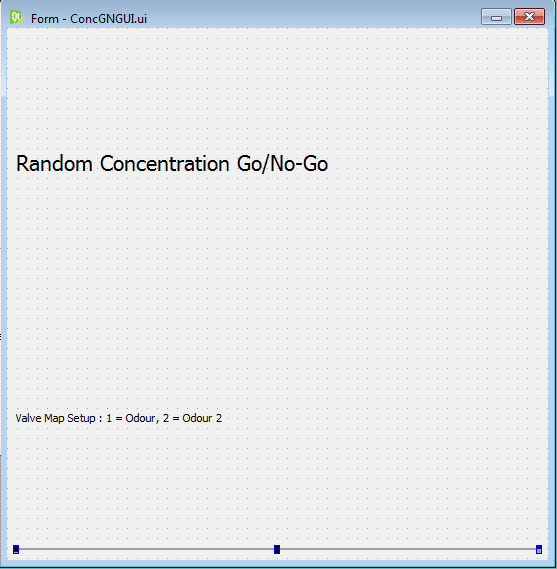


Next, define the user-defined parameters. We will use the same parameters as SimpleGNGWidget, but add a parameter for the lowest concentration allowed for each odour. Use LineEdit and CheckBox widgets for parameter controls, and labels for the titles of each. Set some default values for each parameter, and name each parameter control object for later in-code reference (e.g. Number of Trials will take the object name nTrialsEdit). Make sure to save all the changes to the widget.


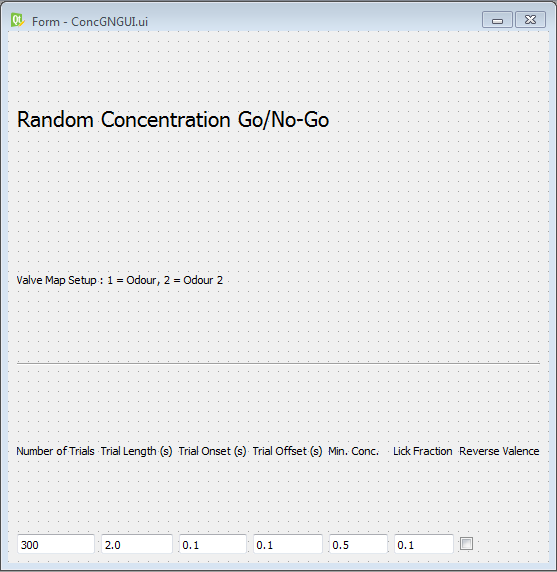


Next, we must convert the UI design into a format that we can interact with in code. Open a console window and navigate into the schedule-generator home folder e.g.:

>> C:\> cd <USER>

>> C:\<USER>> cd <SCHEDULE GENERATOR LOCATION>

>> C:\<USER>\<SCHEDULE GENERATOR LOCATION>>

Then, convert the UI into a .py file using PyQt’s pyuic function. Transfer the saved .ui file into schedule-generator’s Design folder, using the –o flag to overwrite any previous versions:

>> pyuic5 UI/ConcGNGUI.ui –o Designs/concGNGDesign.py

There should now be a file named ‘concGNGDesign.py’ in schedule-generator’s Design folder.

Now we will define the internal logic and data model for our widget. Open the ScheduleWidgets.py file and import the new widget design in the head of the file (along with any other existing imports):

from Designs import corrGNGDesign

Create a new class in the same file to define our new widget:

class ConcGNGWidget(QtWidgets.QWidget, concGNGDesign.Ui_Form):
 def __init__( self, parentUi=None):

 def generate_schedule(self, valence_map):

 def pulse_parameters(self, trial):

This class should inherit from the QWidget class as well as the Ui_Form of the design file we previously created. We also define 3 required methods, __init__ for class instantiation, generate_schedule for generation of the trial sequence and pulse_parameters for mapping of trial parameters onto digital signals. Instantiation will take the same form for almost all widgets:

def __init__(self, parentUi=None):
 super(self.__class__, self).__init__()
 self.setupUi(self)

 self.parentUi = parentUi

 self.valence_map = None

In generate_schedule, we want to return a sequence of trial parameters and their associated names for display in the schedule-generator GUI. This method takes valence_map as its input, which will be passed to the method on loading in schedule-generator. First, we assign variables that will be the same for each trial:

def generate_schedule(self, valence_map):
 lick_fraction = float(self.lickFractionEdit.text())
 n_valves = len(valence_map)

 n_trials = int(self.nTrialsEdit.text())

and then generate a reward sequence according to the standard GNG rule:

reward_sequence = Gen.reward_sequence(n_trials)

Next, we will generate a valve index defining where each channel type is located according to the valence map:

valence_map = np.array(valence_map)
valve_index = (np.where(valence_map == 0)[0],

np.where(valence_map == 1)[0],
 np.where(valence_map == 2)[0])

We will also define the choice of rewarded odour according to the reward valence check box in our UI design:

if not bool(self.reverseValenceCheck.isChecked()):
 rewarded_choice = valve_index[1]
 unrewarded_choice = valve_index[2]
else:
 rewarded_choice = valve_index[2]
 unrewarded_choice = valve_index[1]

For each trial in the schedule we now want to define whether it is rewarded, which valve is used, the valence map used, the lick fraction required for a response, and the concentration level the odour should be delivered at. We will iterate over the number of trials required and define these parameters for each trial, storing each trial entry in a list:

schedule = []
for t in range(n_trials):
 rewarded = reward_sequence[t] == 1

 if rewarded:
 valve = np.random.choice(rewarded_choice, 1) + 1
 else:
 valve = np.random.choice(unrewarded_choice, 1) + 1

 schedule.append([reward_sequence[t], valve, valence_map, lick_fraction])

So far, we have not taken account of our odour concentration parameter that we added in the UI. Minimum allowed concentration will be the same for every trial, so we will define this at the top of the method with the other constant variables:

min_conc = float(self.minConcEdit.text())

Note here that the value will be added as text in the UI, so we must convert it to a float to use in this method. Now we must append our schedule loop to randomly pick a concentration level between our minimum concentration value and 1, as well as appending this value to the output parameters for each trial. We will pick this value randomly on each trial and round it to 2 decimal places:

conc_level = np.round(np.random.uniform(min_conc, 1.0), 2)

schedule.append([reward_sequence[t], valve, valence_map, conc_level, lick_fraction])

So our full generate_schedule function becomes:

def generate_schedule(self, valence_map):
 lick_fraction = float(self.lickFractionEdit.text())
 n_valves = len(valence_map)
 min_conc = float(self.minConcEdit.text())

 n_trials = int(self.nTrialsEdit.text())
 reward_sequence = Gen.reward_sequence(n_trials)

 valence_map = np.array(valence_map)
 valve_index = (np.where(valence_map == 0)[0],
 np.where(valence_map == 1)[0],
 np.where(valence_map == 2)[0])

 if not bool(self.reverseValenceCheck.isChecked()):
 rewarded_choice = valve_index[1]
 unrewarded_choice = valve_index[2]
 else:
 rewarded_choice = valve_index[2]
 unrewarded_choice = valve_index[1]

 schedule = []
 for t in range(n_trials):
 rewarded = reward_sequence[t] == 1

 if rewarded:
 valve = np.random.choice(rewarded_choice, 1) + 1
 else:
 valve = np.random.choice(unrewarded_choice, 1) + 1

 conc_level = np.round(np.random.uniform(min_conc, 1.0), 2)

 schedule.append([reward_sequence[t], valve, valence_map, conc_level, lick_fraction])

 return schedule, ['Rewarded', 'Valve', 'Valence Map', 'Conc. Level', 'Lick Fraction']

Next, we will define how the parameters for each trial are converted into digital signals in the pulse_parameters method. First, we will define parameters that will be the same regardless of the particular trial being parsed:

def pulse_parameters(self, trial):
 params = list()

 onset = float(self.onsetEdit.text())
 offset = float(self.offsetEdit.text())
 length = float(self.trialLengthEdit.text())

The params variable here is a list that will store the particular signal parameters for each active channel. We will now assign variables based on the individual trial passed to the method:

valve = trial[1]
valence_map = trial[2]

conc_level = trial[3]

Now we will loop through each active channel and set its base parameters:

for p in range(len(valence_map)):
 param = {'type': 'Simple',
 'fromDuty': False,
 'fromValues': True,
 'pulse_width': length,
 'pulse_delay': 0.0,
 'fromLength': False,
 'fromRepeats': True,
 'repeats': 0,
 'length': 0.0,
 'isClean': False,
 'isShatter': True,
 'shatter_frequency': 500.0,
 'shatter_duty': conc_level,
 'onset': onset,
 'offset': offset,
 'lick_fraction': trial[3]}

Finally, within the loop if the current index is equal to an active valve, we will activate the signal output by setting the number of pulse repeats to 1, and then append our parameter space to the list of parameters (see Pulse parameters for a full list of available signal definition parameters):

for p in range(len(valence_map)):
 param = {'type': 'Simple',
 'fromDuty': False,
 'fromValues': True,
 'pulse_width': length,
 'pulse_delay': 0.0,
 'fromLength': False,
 'fromRepeats': True,
 'repeats': 0,
 'length': 0.0,
 'isClean': False,
 'isShatter': True,
 'shatter_frequency': 500.0,
 'shatter_duty': conc_level,
 'onset': onset,
 'offset': offset,
 'lick_fraction': trial[3]}

 if p + 1 in valve:
 param['repeats'] = 1

 params.append(param)

We will then output this list of parameters. The final pulse_parameters function will therefore be:

def pulse_parameters(self, trial):
 params = list()

 onset = float(self.onsetEdit.text())
 offset = float(self.offsetEdit.text())
 length = float(self.trialLengthEdit.text())

 valve = trial[1]
 valence_map = trial[2]
 conc_level = trial[3]

 for p in range(len(valence_map)):
 param = {'type': 'Simple',
 'fromDuty': False,
 'fromValues': True,
 'pulse_width': length,
 'pulse_delay': 0.0,
 'fromLength': False,
 'fromRepeats': True,
 'repeats': 0,
 'length': 0.0,
 'isClean': False,
 'isShatter': True,
 'shatter_frequency': 500.0,
 'shatter_duty': conc_level,
 'onset': onset,
 'offset': offset,
 'lick_fraction': trial[3]}

 if p + 1 in valve:
 param['repeats'] = 1

 params.append(param)

 return params

And the full final class definition will be:

class ConcGNGWidget(QtWidgets.QWidget, concGNGDesign.Ui_Form):
 def __init__(self, parentUi=None):
 super(self.__class__, self).__init__()
 self.setupUi(self)

 self.parentUi = parentUi

 self.valence_map = None

 def generate_schedule(self, valence_map):
 lick_fraction = float(self.lickFractionEdit.text())
 n_valves = len(valence_map)
 min_conc = float(self.minConcEdit.text())

 n_trials = int(self.nTrialsEdit.text())
 reward_sequence = Gen.reward_sequence(n_trials)

 valence_map = np.array(valence_map)
 valve_index = (np.where(valence_map == 0)[0],
 np.where(valence_map == 1)[0],
 np.where(valence_map == 2)[0])

 if not bool(self.reverseValenceCheck.isChecked()):
 rewarded_choice = valve_index[1]
 unrewarded_choice = valve_index[2]
 else:
 rewarded_choice = valve_index[2]
 unrewarded_choice = valve_index[1]

 schedule = []
 for t in range(n_trials):
 rewarded = reward_sequence[t] == 1

 if rewarded:
 valve = np.random.choice(rewarded_choice, 1) + 1
 else:
 valve = np.random.choice(unrewarded_choice, 1) + 1

 conc_level = np.round(np.random.uniform(min_conc, 1.0), 2)

 schedule.append([reward_sequence[t], valve, valence_map, conc_level, lick_fraction])

 return schedule, ['Rewarded', 'Valve', 'Valence Map', 'Conc. Level', 'Lick Fraction']

 def pulse_parameters(self, trial):
 params = list()

 onset = float(self.onsetEdit.text())
 offset = float(self.offsetEdit.text())
 length = float(self.trialLengthEdit.text())

 valve = trial[1]
 valence_map = trial[2]
 conc_level = trial[3]

 for p in range(len(valence_map)):
 param = {'type': 'Simple',
 'fromDuty': False,
 'fromValues': True,
 'pulse_width': length,
 'pulse_delay': 0.0,
 'fromLength': False,
 'fromRepeats': True,
 'repeats': 0,
 'length': 0.0,
 'isClean': False,
 'isShatter': True,
 'shatter_frequency': 500.0,
 'shatter_duty': conc_level,
 'onset': onset,
 'offset': offset,
 'lick_fraction': trial[3]}

 if p + 1 in valve:
 param['repeats'] = 1

 params.append(param)

 return params

schedule-generator will automatically detect this new class definition (by reflection), so we can now load the full widget into the main GUI and generate our schedule:


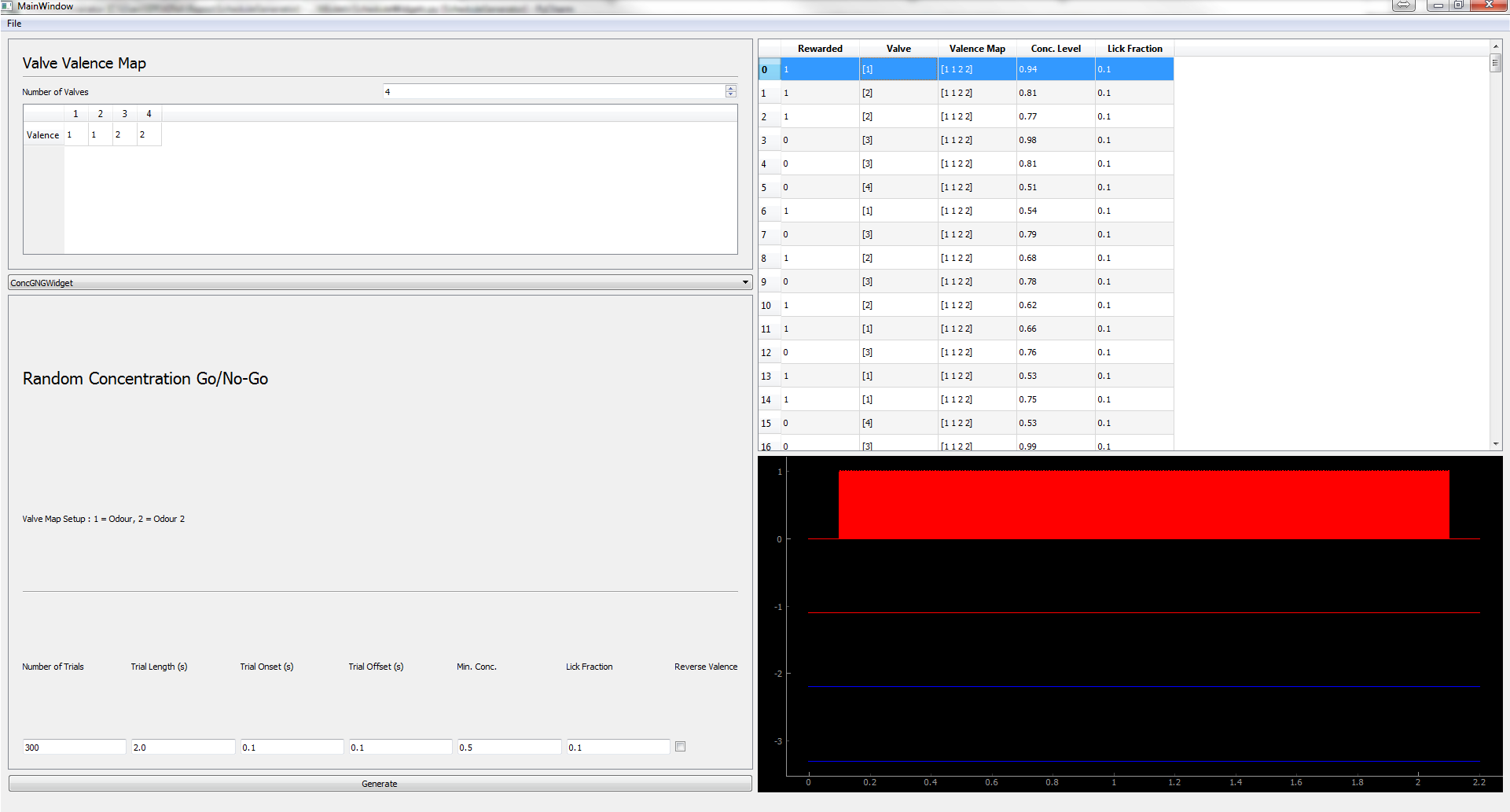


### Pulse parameters

Digital signal shapes are controlled and generated by passing parameters in schedule-generator to the associated PyPulse module. Parameters are structured as a Python dictionary with a global flag (‘type’) that defines the general class of signal to be created, and a list of more specific parameters. These type parameters, their associated specific parameters and the features of the signal they control are listed below.

- Simple – a standard pulse pattern with regular repeating on/off cycles
  - fromDuty: set to True if pulse is to be generated based on duty values
  - frequency: pulse frequency in Hz
  - duty: duty cycle of pulses as a fraction /1
  - fromValues: set to True if pulse is to be generated based on time in seconds of each pulse
  - pulse_width: length in seconds of pulse on time
  - pulse_delay: length of pulse off time
  - fromLength: set to True if total pulse train length is generated based on total time in seconds
  - length: the time of the stimulus in seconds
  - fromRepeats: set to True if total pulse train length is generated based on a desired number of pulse repeats
  - repeats: the number of pulse repeats
  - isClean: set to True if no pulse-width modulation of individual pulses is required
  - isShatter: set to True to pulse-width modulate each pulse
  - shatter_frequency: pulse-width modulation frequency in Hz
  - shatter_duty: pulse-width modulation duty as a fraction /1
  - onset: total amount of off time in seconds before start of pulse train
  - offset: total amount of off time in seconds after end of pulse train
- Noise – generate pulse-width modulated sequence in which PWM duty changes randomly at a given frequency
  - fromLength: set to True if total pulse train length is generated based on total time in seconds
  - length: the length of the stimulus in seconds
  - fromRepeats: set to True if total pulse train length is generated based on a desired number of PWM changes
  - repeats: the number of PWM changes
  - seed: the initialisation seed for the random sequence generating the PWM changes
  - amp_min: the lowest allowed PWM duty as a fraction /1
  - amp_max: the highest allowed PWM duty as a fraction /1
  - shatter_frequency: pulse-width modulation frequency in Hz
  - onset: total amount of off time in seconds before start of pulse train
  - offset: total amount of off time in seconds after end of pulse train
- RandomNoise – a standard pulse pattern with regular repeating on/off cycles in which each pulse can be pulse-width modulated with random, noise-like duty cycles
  - fromDuty: set to True if pulse is to be generated based on duty values
  - frequency: pulse frequency in Hz
  - duty: duty cycle of pulses as a fraction /1
  - fromValues: set to True if pulse is to be generated based on time in seconds of each pulse
  - pulse_width: length in seconds of pulse on time
  - pulse_delay: length of pulse off time
  - fromLength: set to True if total pulse train length is generated based on total time in seconds
  - length: the time of the stimulus in seconds
  - fromRepeats: set to True if total pulse train length is generated based on a desired number of pulse repeats
  - repeats: the number of pulse repeats
  - shatter_frequency: pulse-width modulation frequency in Hz
  - target_duty: pulse-width modulation duty as a fraction /1 around which the true PWM duty value will randomly fluctuate
  - extend: set to True is pulse train is to be offset in time
  - amp_min: the lowest allowed PWM duty as a fraction /1
  - amp_max: the highest allowed PWM duty as a fraction /1
  - onset: total amount of off time in seconds before start of pulse train
  - offset: total amount of off time in seconds after end of pulse train
  - shadow: set to True if the pulse should compensate the duty of another pulse
